# The Impact of Hotspot-Targeted Interventions on Malaria Transmission in Rachuonyo South District in the Western Kenyan Highlands: A Cluster-Randomized Controlled Trial

**DOI:** 10.1371/journal.pmed.1001993

**Published:** 2016-04-12

**Authors:** Teun Bousema, Gillian Stresman, Amrish Y. Baidjoe, John Bradley, Philip Knight, William Stone, Victor Osoti, Euniah Makori, Chrispin Owaga, Wycliffe Odongo, Pauline China, Shehu Shagari, Ogobara K. Doumbo, Robert W. Sauerwein, Simon Kariuki, Chris Drakeley, Jennifer Stevenson, Jonathan Cox

**Affiliations:** 1 Radboud Institute for Health Sciences, Department of Medical Microbiology, Radboud University Medical Center, Nijmegen, the Netherlands; 2 Department of Infectious & Tropical Diseases, London School of Hygiene & Tropical Medicine, London, United Kingdom; 3 MRC Tropical Epidemiology Group, London School of Hygiene & Tropical Medicine, London, United Kingdom; 4 Department of Mathematical Sciences, University of Bath, Bath, United Kingdom; 5 Kenya Medical Research Institute/Centre for Global Health Research, Kisumu, Kenya; 6 Malaria Research and Training Center, University of Sciences, Techniques and Technologies of Bamako, Bamako, Mali; 7 Johns Hopkins Malaria Research Institute, Johns Hopkins Bloomberg School of Public Health, Baltimore, Maryland, United States of America; University of Melbourne, AUSTRALIA

## Abstract

**Background:**

Malaria transmission is highly heterogeneous, generating malaria hotspots that can fuel malaria transmission across a wider area. Targeting hotspots may represent an efficacious strategy for reducing malaria transmission. We determined the impact of interventions targeted to serologically defined malaria hotspots on malaria transmission both inside hotspots and in surrounding communities.

**Methods and Findings:**

Twenty-seven serologically defined malaria hotspots were detected in a survey conducted from 24 June to 31 July 2011 that included 17,503 individuals from 3,213 compounds in a 100-km^2^ area in Rachuonyo South District, Kenya. In a cluster-randomized trial from 22 March to 15 April 2012, we randomly allocated five clusters to hotspot-targeted interventions with larviciding, distribution of long-lasting insecticide-treated nets, indoor residual spraying, and focal mass drug administration (2,082 individuals in 432 compounds); five control clusters received malaria control following Kenyan national policy (2,468 individuals in 512 compounds). Our primary outcome measure was parasite prevalence in evaluation zones up to 500 m outside hotspots, determined by nested PCR (nPCR) at baseline and 8 wk (16 June–6 July 2012) and 16 wk (21 August–10 September 2012) post-intervention by technicians blinded to the intervention arm. Secondary outcome measures were parasite prevalence inside hotpots, parasite prevalence in the evaluation zone as a function of distance from the hotspot boundary, *Anopheles* mosquito density, mosquito breeding site productivity, malaria incidence by passive case detection, and the safety and acceptability of the interventions. Intervention coverage exceeded 87% for all interventions. Hotspot-targeted interventions did not result in a change in nPCR parasite prevalence outside hotspot boundaries (*p* ≥ 0.187). We observed an average reduction in nPCR parasite prevalence of 10.2% (95% CI −1.3 to 21.7%) inside hotspots 8 wk post-intervention that was statistically significant after adjustment for covariates (*p =* 0.024), but not 16 wk post-intervention (*p =* 0.265). We observed no statistically significant trend in the effect of the intervention on nPCR parasite prevalence in the evaluation zone in relation to distance from the hotspot boundary 8 wk (*p =* 0.27) or 16 wk post-intervention (*p =* 0.75). Thirty-six patients with clinical malaria confirmed by rapid diagnostic test could be located to intervention or control clusters, with no apparent difference between the study arms. In intervention clusters we caught an average of 1.14 female anophelines inside hotspots and 0.47 in evaluation zones; in control clusters we caught an average of 0.90 female anophelines inside hotspots and 0.50 in evaluation zones, with no apparent difference between study arms. Our trial was not powered to detect subtle effects of hotspot-targeted interventions nor designed to detect effects of interventions over multiple transmission seasons.

**Conclusions:**

Despite high coverage, the impact of interventions targeting malaria vectors and human infections on nPCR parasite prevalence was modest, transient, and restricted to the targeted hotspot areas. Our findings suggest that transmission may not primarily occur from hotspots to the surrounding areas and that areas with highly heterogeneous but widespread malaria transmission may currently benefit most from an untargeted community-wide approach. Hotspot-targeted approaches may have more validity in settings where human settlement is more nuclear.

**Trial registration:**

ClinicalTrials.gov NCT01575613

## Abstract 2

In a cluster-randomized controlled trial, Teun Bousema and colleagues investigate whether interventions targeted to malaria hotspots in the western Kenyan highlands can reduce transmission in surrounding areas.

## Editors' Summary

**Background:**

In 2015, there were about 200 million cases of malaria (a mosquito-borne parasitic disease) worldwide and about 438,000 malaria-related deaths. Most of these deaths were caused by *Plasmodium falciparum*, which is transmitted to people by night-flying *Anopheles* mosquitoes. When infected mosquitoes bite people, they inject “sporozoites,” a parasitic form that replicates in the liver. After a few days, the liver releases “merozoites,” which invade red blood cells, where they replicate before bursting out and infecting more red blood cells. This increase in parasitic burden causes recurring flu-like symptoms and can cause organ damage and death if not treated promptly with antimalarial drugs. Infected red blood cells also release “gametocytes,” which infect mosquitoes when they take a blood meal. In the mosquito, gametocytes multiply and develop into sporozoites, which completes the parasite’s life cycle. Malaria can be prevented by controlling *Anopheles* mosquitoes and by avoiding mosquito bites. Methods for controlling mosquitoes include treating stagnant water with “larvicides” to kill immature mosquitoes and spraying houses with insecticides (indoor residual spraying); sleeping under insecticide-treated bednets is an effective way to avoid mosquito bites. Treatment with antimalarial drugs also decreases malaria transmission.

**Why Was This Study Done?:**

Improved malaria prevention has reduced malaria-related deaths over recent years, and experts are now trying to devise strategies to eradicate malaria. Targeting malaria transmission hotspots—regions where the intensity of transmission is higher than in surrounding areas—is one potential strategy for reducing malaria transmission. Mosquito densities are highest in hotspots, and people living in hotspots may transmit malaria parasites to a large number of mosquitoes that can then spread malaria transmission to wider areas. Targeting malaria hotspots might, therefore, benefit communities in both the targeted region and the surrounding area. Here, the researchers look for malaria hotspots in Rachuonyo South District in the western Kenyan highlands, an area where malaria transmission intensity is generally low, and undertake a cluster-randomized controlled trial to determine the impact of hotspot-targeted malaria control interventions on malaria transmission inside hotspots and in surrounding communities. A cluster-randomized trial compares outcomes in groups (clusters) of people (here, people living in different malaria hotspots) randomly assigned to receive different interventions.

**What Did the Researchers Do and Find?:**

Using survey results and spatial scanning techniques, the researchers detected 27 serologically defined malaria hotpots (clusters of people with antibodies to the malaria parasite) in Rachuonyo South District. Of these, ten hotspots were at least 1.5 kilometers from the nearest neighboring hotspot (allowing space for evaluation zones and a buffer between hotspots). During the following peak malaria transmission period, they randomly allocated five of these hotspots to targeted interventions (larvicide treatment of stagnant water, community distribution of insecticide-treated bednets, indoor residual spraying, and mass drug administration) and five hotspots to the control intervention (the Kenyan national policy of annual indoor residual spraying, routine case management at clinics, and bednet distribution at antenatal clinics). Intervention coverage exceeded 87% for all the components of the targeted intervention. However, the intervention did not change the prevalence of parasites (the proportion of the population carrying parasites) at 8 or 16 weeks post-intervention in the evaluation zones—the areas 1–500 meters from the border of the hotspots (the study’s primary outcome). There was a statistically significant reduction in parasite prevalence inside intervention hotspots at 8 but not 16 weeks post-intervention (a statistically significant change is one unlikely to have happened by chance). Finally, similar numbers of individuals from the intervention and control clusters attended local health facilities and were found to have confirmed clinical malaria, and there was no significant difference between intervention and control clusters in the number of mosquitoes caught within hotspots or evaluation zones.

**What Do These Findings Mean?:**

These findings show that, despite high coverage, the impact of interventions targeting malaria hotspots in Rachuonyo South District on parasite prevalence was modest, transient, and restricted to the targeted hotspot. It may be that the number of clusters included in this study was too small to detect any subtle effects of hotspot-targeted interventions. Moreover, the effect of the targeted interventions may have been masked by other ongoing interventions in the region. However, these findings suggest that malaria transmission in Rachuonyo South District may not occur primarily from hotspots to the surrounding areas. Thus, areas where malaria transmission is widespread but includes hotspots might benefit most from an untargeted community-wide approach to malaria prevention.

**Additional Information:**

This list of resources contains links that can be accessed when viewing the PDF on a device or via the online version of the article at http://dx.doi.org/10.1371/journal.pmed.1001993.

- Information is available from the World Health Organization on malaria (in several languages); its *Global Technical Strategy for Malaria 2016–2030* provides a framework to guide countries towards malaria elimination; the *World Malaria Report 2015* describes the current global malaria situation and includes information on malaria in individual African countries and recommended policies and strategies for malaria control
- The US Centers for Disease Control and Prevention provides information on malaria (in English and Spanish), including information about malaria prevention and personal stories about malaria
- The UK National Health Service Choices website also provides information about malaria, including a personal story
- Information is available from the Roll Back Malaria Partnership on the global control of malaria (in English and French)
- MedlinePlus provides links to additional information on malaria (in English and Spanish)
- More information about this trial is available

## Introduction

The transmission of many infectious agents, including malaria, is highly heterogeneous in space and time. In the last decade, considerable efforts have been made to better estimate the global and local burden of malaria. At a micro-epidemiological scale in endemic areas, numerous factors influence malaria transmission dynamics, including distance to the nearest mosquito breeding site [1–4], wind direction [5], vegetation [6], house construction features [1,3,4], and human genetic [2,3,7] and behavioral factors [1–3,8]. Variations in these factors over a small area can result in spatially heterogeneous transmission and can result in malaria hotspots, where transmission intensity is higher than in the surrounding areas. These malaria hotspots may be present in all malaria endemic areas but are most readily identifiable in areas of low transmission intensity, where malaria incidence, parasite prevalence, and mosquito exposure may be elevated inside hotspots [5,6,9].

Malaria control efforts targeted to transmission hotspots may have benefits for both the targeted area and the wider community. Mosquito densities are highest in hotspots, and individuals in hotspots may amplify transmission by transmitting malaria parasites to a large number of mosquitoes that fuel transmission to wider areas. This amplified transmission can lead to 1.5- to 4-fold increases in the basic reproductive number of malaria parasites [9–11]. Successful targeting of malaria control efforts to hotspots may therefore be a highly efficient method to reduce malaria transmission in a wider area and achieve community protection by eliminating transmission in a relatively small geographical area [9,11,12]. Such targeted interventions are likely to become increasingly important tools in malaria elimination efforts once transmission in an area has decreased but is maintained in hotspots of malaria transmission [13]. We hypothesized that combined malaria control interventions targeted at hotspots could reduce malaria transmission not only inside these hotspots but also in adjacent areas. To test this, we identified hotspots of malaria transmission in a low endemic area in the western Kenyan highlands, and conducted a cluster-randomized controlled trial to measure the effect of hotspot-targeted interventions in evaluation zones surrounding malaria hotspots.

## Methods

The original protocol for the cluster-randomized trial (S1 Text) and the supporting CONSORT checklist (S2 Text) are provided. A detailed study protocol was previously published [14].

### Ethics Statement

This study was approved by the ethical committees of the London School of Hygiene & Tropical Medicine (LSHTM 5721) and the Kenya Medical Research Institute (KEMRI) (SSC 1802/2163/2495). Consenting procedures are described in detail elsewhere [14]. Prior to the community survey (2011) and randomization for the cluster-randomized trial (2012), community sensitization meetings were organized. Informed written consent was sought from all individuals participating in surveys or, where appropriate, their parents or guardians. Written assent was obtained from all children aged 13–17 y, accompanied by a consent form signed by the parent or guardian. Prior to indoor residual spraying (IRS) and the distribution of long-lasting insecticide-treated nets (LLINs), written informed consent was obtained from the head of compound. Prior to focal mass drug administration (MDA), individual written consent and/or assent was obtained. Larviciding commenced after written approval from the Division of Malaria Control and Kenyan Pest Control Products Board and with the oral approval of the district administration, fisheries, and persons responsible for any privately owned permanent water bodies such as fishponds.

### Study Area and Population

**Fig 1:**
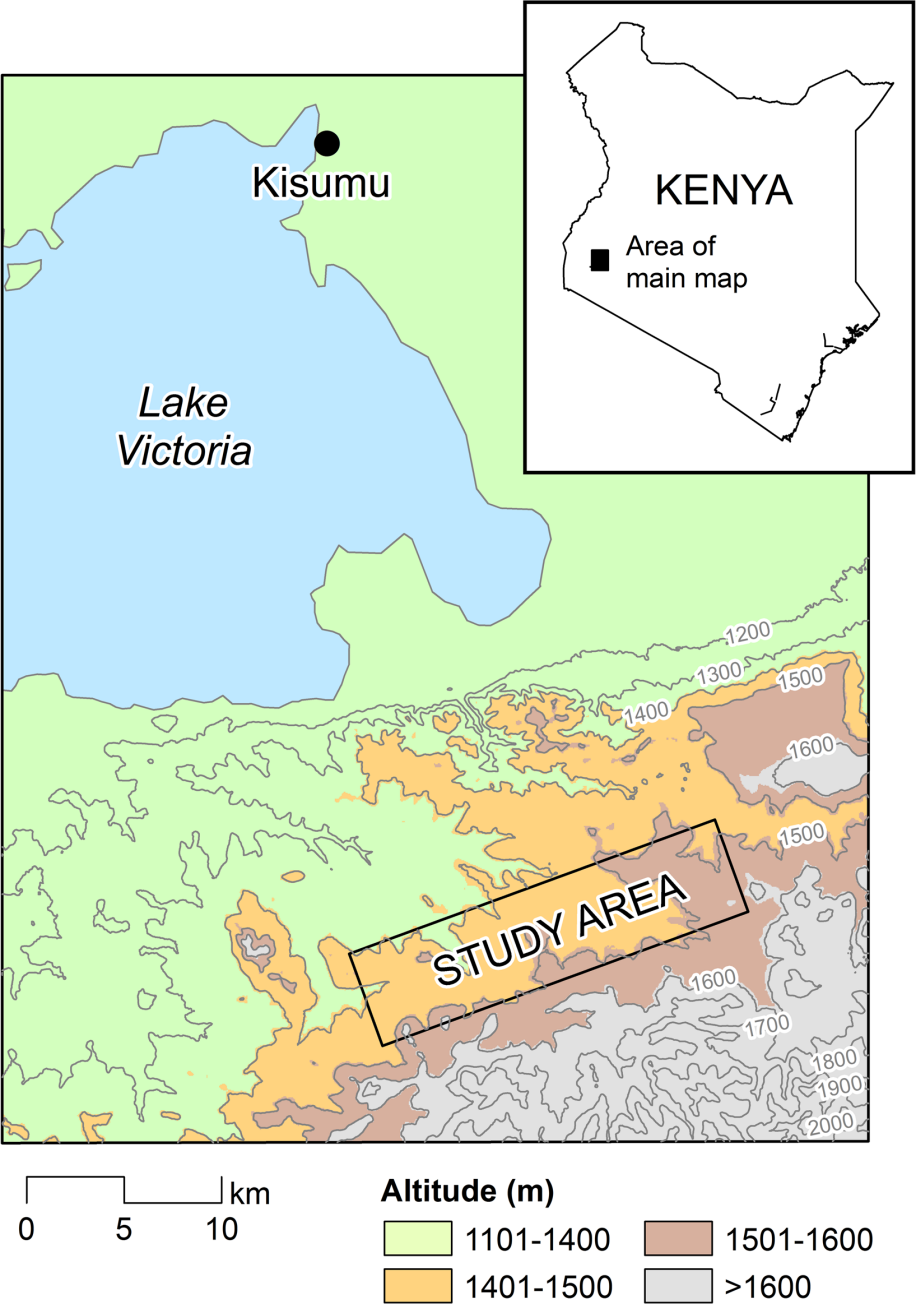
The study area in the highlands of western Kenya. The study area comprised a 5 × 20 km rectangle in Rachuonyo South District, Nyanza Province.

A 5 × 20 km study area located at 1,400 m to 1,650 m above sea level was selected in Rachuonyo South District, western Kenya (34.75–34.94^○^E, 0.41–0.53^○^S) and divided into 500 × 500 m cells (Fig 1). Compounds in the study area are distributed broadly across a rolling landscape intersected by streams and rivers; compounds typically comprised a median of two houses (interquartile range [IQR] 1 to 3) and 5 inhabitants (IQR 3 to 7). Malaria transmission in the area is seasonal and associated with seasonal rains that typically peak between March and June and between October and November. Transmission intensity in the study area is generally low; mean parasite prevalence in community surveys in 2010 by rapid diagnostic test (RDT) was 10%, with marked spatial heterogeneity [14,15]. The principal malaria vectors are *Anopheles gambiae* s.s., *A*. *arabiensis*, and *A*. *funestus* [16]. The Ministry of Public Health and Sanitation has been involved in the distribution of insecticide-treated nets (ITNs) in the area for many years; ITN ownership in children below 5 y of age was 82.7% in 2010 [15]. Since 2009, the Division of Malaria Control has carried out annual IRS campaigns with pyrethroids in structures that serve as sleeping spaces, reaching ≥70% of all compounds [14].

### Study Design

#### Community survey and hotspot detection

The procedures for hotspot detection and the cluster-randomized trial are described in detail elsewhere [14]. We imported geo-location data for all structures in the study area, collected from high-resolution satellite data (QuickBird; DigitalGlobe) into a geographical information system (ArcGIS 9.2; Esri). Manually digitizing structures yielded 8,632 structures, with a median of 45 structures (IQR 35–52) per 500 × 500 m cell. For hotspot identification, we carried out a community survey in 2011 to obtain measurements from ≥50 individuals per cell, sampling in equal ratios from predefined age strata (≤5 y, 6–10 y, 11–15 y, 16–25 y and >25 y). Twenty field teams consisting of one enumerator, one field worker, and one person trained for blood sample collection were equipped with high-resolution maps and a handheld GPS receiver (GPSMAP 62s; Garmin International) with preloaded waypoints for 16 randomly selected compounds and cell boundaries. Compounds were eligible for sampling if at least one adult and one child (<15 y) were permanent residents (defined as sleeping regularly in the structure) and written informed consent was obtained. If a selected compound did not satisfy these criteria, the nearest non-selected inhabited compound was selected as a replacement. Participating individuals in the community survey were screened by axillary thermometer for fever, and those with fever were tested by RDT (HRP-2, Paracheck, Orchid Biomedical Systems) for on-site malaria diagnosis and treatment with artemether-lumefantrine (AL) (Coartem, Novartis) if RDT positive. Febrile individuals who were RDT negative, pregnant, or below 6 mo of age were accompanied to a local health facility for a full clinical assessment and treatment.

Finger prick blood was collected from all participants on filter paper (3MM Whatman). Filter papers were stored with desiccant at −20°C until used to detect antibodies against *Plasmodium falciparum* apical membrane antigen-1 (AMA-1) and merozoite surface protein-1 (MSP-1_19_) using established ELISA methodologies [17,18] and to detect parasite prevalence by nested PCR (nPCR) targeting the 18S rRNA gene [18]. All molecular and serological assays for the 2011 community survey were conducted at the KEMRI/Centers for Disease Control and Prevention (CDC) laboratories in Kisumu, Kenya. Because of the labor intensiveness and resource intensiveness of DNA extraction and nPCR and the necessity of obtaining results on nPCR parasite prevalence prior to hotspot selection, nPCR was only performed on only a random selection of samples (12,912/16,381). Data on demography, travel behavior, sleeping times, history of malaria treatment, and use of protective measures were collected on a personal digital assistant (HP Ipaq 210, Windows Mobile 6.1) with a precoded questionnaire programmed in Visual Basic (Visual CE v11.0).

#### Spatial scanning for hotspot detection

Using data from this community survey, we screened for local clustering of seroreactivity using SaTScan software [19]. Spatial scans were based on the prevalence of antibodies to either AMA-1 or MSP-1_19_ (Bernoulli model) and log_10_-transformed optical density values for AMA-1 and MSP-1_19_, where the highest age-adjusted optical density value for either antigen was used in a normal probability model. Circular and elliptic windows were used to systematically scan segments of the study area using a 2 × 4 km rolling window, allowing for a hotspot size with <1 km radius and <25% of the population of each window scanned. This rolling window was chosen since malaria transmission was expected to show considerable variation with altitude, as well as micro-epidemiological variation within altitude bands. We thus scanned sections of the study area for variations in antibody prevalence and density compared to local average values. A hotspot was defined as an area for which there was strong evidence (*p <* 0.05) that the observed prevalence and density of combined AMA-1 and MSP-1_19_ antibodies were higher than expected as calculated from the prevalence within the rolling window [14]. nPCR data were not used for screening for local clustering or hotspot detection but were used to detect current infections in serologically defined hotspots and thereby confirm ongoing malaria transmission in these localities.

### Cluster-Randomized Trial on the Effect of Hotspot-Targeted Interventions

#### Sample size

In the absence of published studies that quantified the impact of hotspot-targeted interventions, we estimated the predicted impact of our interventions using an individual-based simulation model [20]. Simulations of hotspot-targeted LLIN distribution and IRS implementation indicated the possible interruption of transmission, both inside and outside malaria hotspots, reducing overall parasite prevalence to <5%, in a manner that is apparent in the first season and sustainable in the following years [14]. A previous study on the community benefits of ITNs indicated that the indirect beneficial effect on malaria transmission is most pronounced within 500 m of the intervention area [21]. We used these findings to design our trial and define evaluation zones that were dichotomized into two categories based on distance from the hotspot boundary (1–249 m and 250–500 m). Assuming a sample of 200 randomly selected individuals in the evaluation zone of each cluster, a coefficient of variation of true proportions between clusters within each group (*k*) of 0.4, and a mean nPCR parasite prevalence of 15% and 5% in the control and intervention clusters, respectively, the study would require five clusters per study arm for 80% power at the 5% significance level.

#### Randomization

Clusters were serologically defined hotspots with a surrounding 500-m evaluation zone. Hotspots were eligible for the intervention trial if no other hotspots were detected within 1.0 km of the border of the evaluation zone. Ten selected clusters were allocated to the intervention or control arm by a trial statistician using computer-generated random tables.

#### Intervention

In the intervention arm, four interventions were concurrently rolled out inside hotspots, starting in March 2012 before the long rainy season. (i) All stagnant water bodies (permanent and temporary) were treated on a weekly basis with water-dispersible granule formulations of the commercial strains of *Bacillus thuringiensis* var. *israelensis* (VectoBac W, Valent BioSciences) [22] by a team that was permanently present in the hotspot with one supervisor per cluster. Larviciding continued from March to August 2012. (ii) One LLIN (PermaNet W 3.0; Vestergaard Frandsen) was provided for every two compound members by a team of two field workers per cluster. Leaflets with instructions in the local dialect and verbal explanations about correct usage were provided. The use of LLINs was confirmed in a follow-up visit 6 to 8 wk after initial distribution during which the presence and quality of study LLINs were observed and replacement LLINs issued if required. Any adverse events following net use were recorded by the study team during the LLIN follow-up visit and care given as needed. (iii) IRS with the pyrethroid insecticide lambda-cyhalothrin (ICON, Syngenta) was performed by district teams in all eligible compound structures, supervised by two study staff per intervention cluster. Sensitization visits were undertaken prior to the visits of IRS teams to maximize the presence of at least one compound member on the days of spraying, to maximize compound participation. Adverse events associated with the insecticides used in intervention compounds were reported to the study team during the LLIN follow-up visit and care given as needed. (iv) All compounds were visited to determine whether they were eligible for focal MDA as described in detail elsewhere [23]; MDA was implemented by seven teams that each consisted of two fieldworkers, one local guide, and one supervisor who was shared between two teams. A sentinel population of all febrile individuals (tympanic temperature > 37.5°C) and all individuals aged 6 mo–15 y was screened for malaria infection by RDT (HRP-2- and pLDH-based First Response W, Premier Medical Corporation). If one or more compound members were RDT positive, all compound members received a curative dose of AL [14]. Half of the doses (doses 1, 3, and 5, from a total of six) were directly observed and given with fatty food (>1.5 g fat); the other doses were taken without supervision. AL blister packs were collected for adherence monitoring. We used three measures of coverage for MDA: the proportion of compounds participating in screening of the sentinel population, the proportion of compounds eligible for MDA based on ≥1 RDT-positive inhabitant, and the proportion of inhabitants of eligible compounds who received all six doses of AL. Adverse effects monitoring was conducted in conjunction with the daily drug supervision visits. Any symptoms reported on the final day of treatment were recorded and followed up clinically.

Control clusters and evaluation zones received malaria control following Kenyan national policy: annual IRS (ICON, lambda-cyhalothrin), which began in late April 2012 and was completed in June/July 2012, routine case management at health facilities (i.e., all suspected cases of malaria were treated with AL after confirmation of infection by microscopy or RDT), and distribution of LLINs at antenatal clinics. No community distribution of LLINs was carried out in control clusters during the study period.

#### Primary and secondary outcome measures

The primary outcome measure was parasite prevalence in the evaluation zone surrounding malaria hotspots, measured by nPCR. Secondary outcome measures were (i) parasite prevalence inside hotspots, (ii) parasite prevalence in the evaluation zone as a function of distance from the hotspot boundary, (iii) *Anopheles* mosquito density, (iv) mosquito breeding site productivity, (v) the number of malaria cases reported at health facilities by passive case detection (PCD), and (vi) the safety and acceptability of the interventions. As an exploratory endpoint, we determined the number of *P*. *falciparum* clones (complexity of malaria infections) inside and outside targeted hotspots.

#### Follow-up

Cross-sectional surveys were conducted in 22 March–15 April 2012 prior to the intervention (baseline) and then at 8 wk (16 June–6 July, corresponding to the peak of the rainy season) and 16 wk (21 August–10 September, corresponding to the end of the rainy season) after the intervention. In each survey, 25 compounds each were randomly selected from the following settings: (i) inside hotspots, (ii) 1–249 m from the hotspot boundary, and (iii) 250–500 m from the hotspot boundary. All occupants in selected compounds older than 6 mo of age were sampled. Follow-up surveys were conducted by a total of 15 teams that each comprised two fieldworkers, one local guide, and one supervisor who was shared between two teams. Teams were equipped with high-resolution maps and GPS receivers with preloaded waypoints, as described above. In intervention hotspots, data were available for all compounds at baseline and were therefore included in the analysis.

#### Laboratory methods

All finger prick blood samples collected at baseline (*n =* 3,808) and 8 wk (*n =* 3,817) and 16 wk after the intervention (*n =* 3,955) were tested for *P*. *falciparum* parasitemia by nPCR targeting the 18S rRNA gene [18]. All nPCR-positive samples were tested for the presence of multiple allelic forms using the polymorphic merozoite surface protein-2 gene [24].

### Passive Surveillance for Clinical Malaria Incidence

All study participants from intervention and control clusters (hotspots and surrounding evaluation zones) received compound identification cards that were distributed during the pre-intervention survey in 2012 [14]. PCD was carried out in government and mission health facilities that covered intervention and control clusters. All facilities with a working laboratory and full-time laboratory technician were eligible for inclusion, resulting in the inclusion of three facilities in the PCD and exclusion of nine facilities without functioning laboratories. PCD took place between 27 February and 3 September 2012 for a total of 27 wk [25] and relied on RDT-based diagnosis to ensure comparable sensitivity and specificity of malaria diagnosis at the participating facilities [26]. Any patient suspected of malaria who attended one of the three PCD facilities was asked for their compound identification card and examined for fever and symptoms of malaria. If an individual was febrile (temperature > 37.5°C), an RDT was administered. Clinic attendance, the number of clinical malaria cases (defined as fever in combination with a positive RDT), and compound number information were recorded. Spot checks of PCD facilities took place on a fortnightly basis to assess the completeness of records.

### Entomological Surveys

Mosquito exposure was monitored in three intervention and three control clusters; in each of the clusters, four compounds within hotspot boundaries and eight compounds located in the evaluation zone were randomly selected for monitoring. Standard CDC light traps with a 6.3 V incandescent light bulb (John W. Hock Company) were set indoors from 6 p.m. to 6 a.m. 1.5 m above the ground at the foot end of occupied bednets [27]. Adult female anopheline densities were recorded. Mosquito breeding site productivity was abandoned as a secondary outcome because of the labor intensiveness of the larviciding performed by the same teams. Instead, the presence or absence of anopheline larvae and pupae was determined in 15 permanent water bodies in intervention hotspots, using 250-ml dippers and aiming for five and ten dips in water bodies smaller than 5 m^2^ and larger than 5 m^2^, respectively.

### Data Analysis

Statistical analyses were performed using Stata (v. 13, StataCorp). All analyses were based on intention to treat, whereby all clusters were included in the analysis, regardless of the level of coverage. The main outcome measure was nPCR parasite prevalence in the evaluation zone surrounding hotspots. Each cluster was divided into two areas for the analysis: the hotspot and the evaluation zone. To examine parasite prevalence in the evaluation zone as a function of distance from the hotspot boundary (secondary outcome), evaluation zones were stratified into compounds located 1–249 m and 250–500 m from the hotspot boundary. For each survey, comparisons between control and intervention clusters were made between hotspots as well as each stratum of the evaluation zone. Each comparison was made using a *t*-test on the cluster-level nPCR parasite prevalence. Measures of complexity of infection and prevalence were also compared separately for each of the strata by a *t*-test of cluster-level means. Allelic richness, a metric for allelic diversity [28], was calculated using the FSTAT software (v 2.9.3.2). Analyses for nPCR parasite prevalence were carried out adjusting for baseline prevalence and known malaria risk factors in the area: age group (≤5, 6–15, 16–25, >25 y), sex, altitude (<1,450, 1,450–1,500, >1,500 m), and living in a house with open eaves [15]. These covariates, which were not part of the intervention, were defined a priori and were included simultaneously in a single prespecified adjusted analysis. This was done by first performing an individual-level logistic regression with only the factors we wished to adjust for as the predictors while excluding the intervention status of the cluster. This regression was then used to predict the prevalence in each cluster. We then calculated cluster-level residuals as the difference between the observed and expected prevalence and compared the residuals of control and intervention clusters using a *t*-test [29]. Since complexity of infection is count data with an extra-Poisson variation, negative binomial regression was used to estimate cluster-level means. To investigate whether the intervention effect varied by distance from the hotspot, a linear regression model of the cluster-level means was used. A model with distance from hotspot (categorized as 0 m, 1–249 m, and 250–500 m) and intervention but no interaction term for these factors was compared to a model with an interaction between intervention and distance.

Data for this study were deposited in the Dryad Digital Repository [30].

## Results

### Detection of Malaria Hotspots

**Fig 2:**
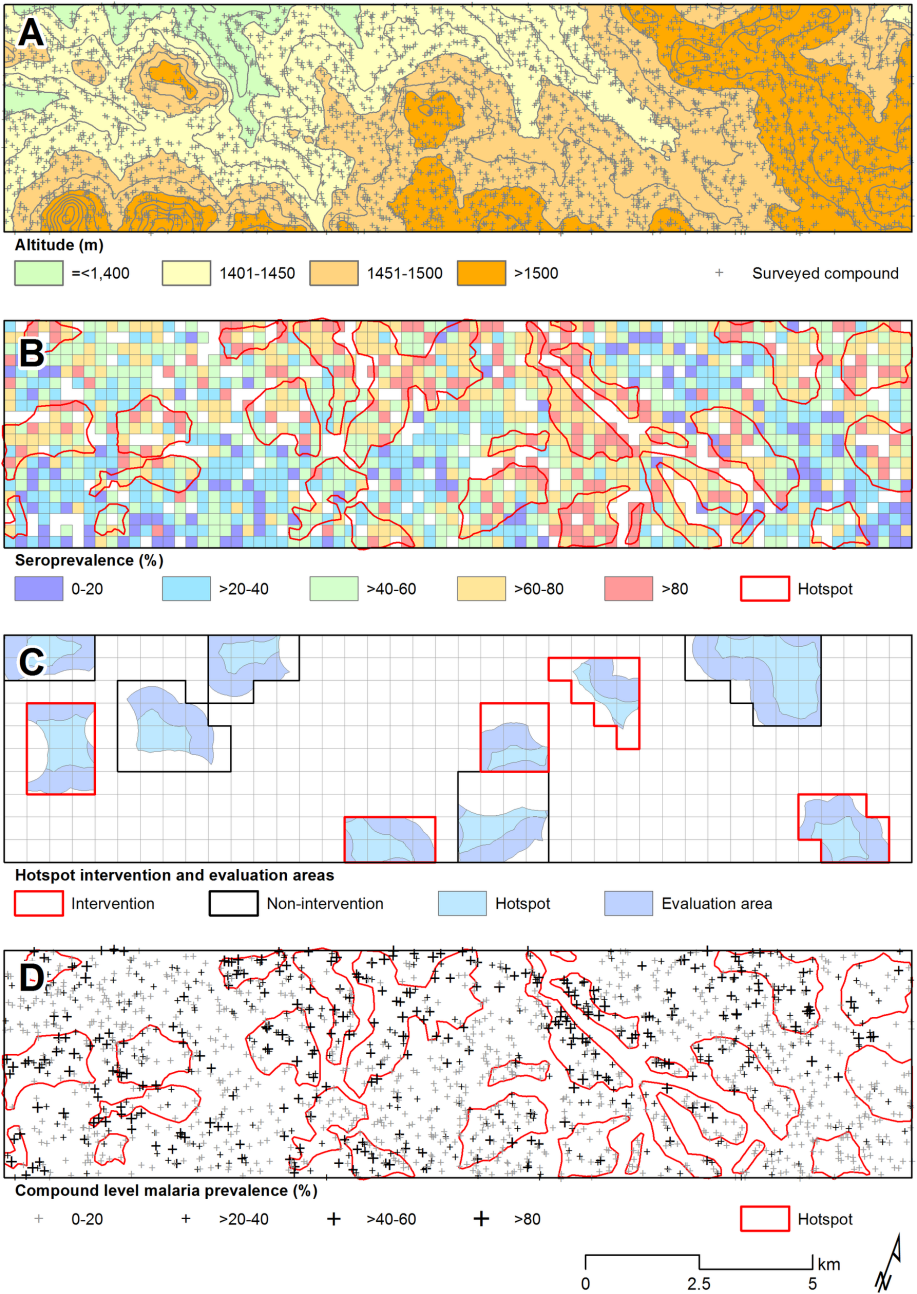
Spatial variation in malaria antibody prevalence and nPCR parasite prevalence in the study area in Rachuonyo South District during a community survey conducted in June–July 2011. (A) Distribution of sampled compounds and variations in altitude across the study site (contour interval = 25 m). (B) Combined seroprevalence (for AMA-1 or MSP-1_19_) for individual 250 × 250 m sub-cells and the location of 27 significant hotspots derived from spatial scan analysis of compound-level data. (C) The ten hotspots that were selected for the cluster-randomized trail, presented with evaluation zones. (D) The 27 serological hotspot locations are overlaid on a map of nPCR-detected malaria parasite prevalence for compounds consisting of >3 individuals.

**Fig 3:**
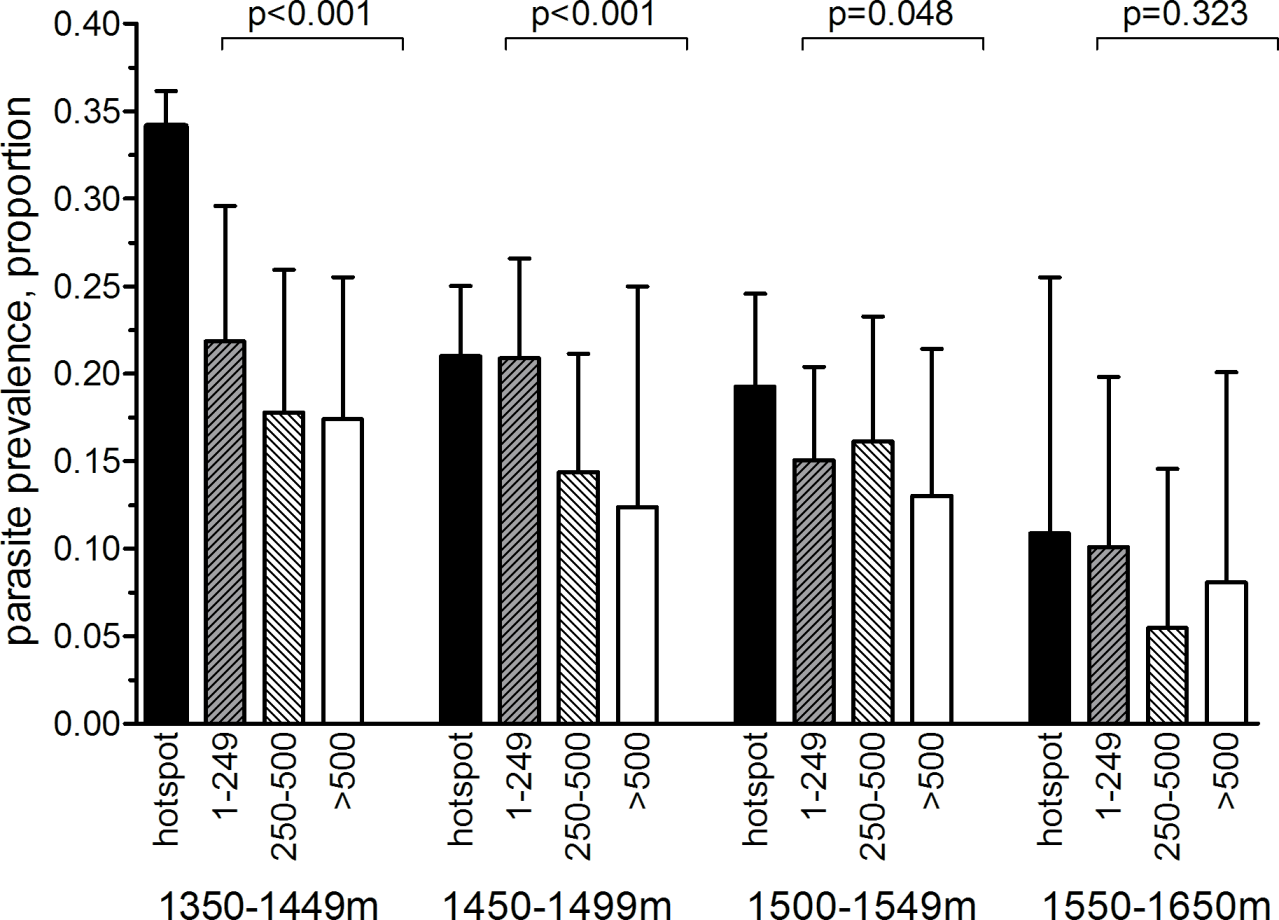
Malaria parasite prevalence by nPCR inside and outside serologically defined hotspots in the study area in Rachuonyo South District during a community survey conducted in June–July 2011. nPCR-based parasite prevalence is plotted for individuals residing inside 27 serologically defined hotspots (black bars), 1–249 meters from the hotspot boundary (grey hatched bars), 250–500 m from the hotspot boundary (open hatched bars), and >500 meters from the hotspot boundary (open bars). Parasite prevalence by nPCR is shown per altitude band. Error bars indicate the upper limit of the 95% confidence interval; the *p*-value for the trend test is given, adjusting for correlations between observations from individuals living in the same compound. The number of individuals for whom samples were available for nPCR inside hotspot boundaries was 2,222 individuals (1,350–1,449 m), 2,494 (1,450–1,499 m), 1,348 (1,500–1,549 m), and 118 (1,550–1,650 m). The number of individuals for 1–249 m from hotspot boundaries was 698 (1,350–1,449 m), 1,248 (1,450–1,499 m), 1,113 (1,500–1,549 m), and 246 (1,550–1,650 m). The number of individuals for 250–500 m from hotspot boundaries was 544 (1,350–1,449 m), 681 (1,450–1,499 m), 661 (1,500–1,549 m), and 164 (1,550–1,650 m). The number of individuals for >500 m from hotspot boundaries was 544 (1,350–1,449 m), 176 (1,450–1,499 m), 405 (1,500–1,549 m), and 135 (1,550–1,650 m).

In the community survey conducted between 24 June and 31 July 2011, a total of 17,503 individuals were sampled, residing in 3,213 compounds across the 100-km^2^ study area (Fig 2A). nPCR-detected parasite prevalence was 20.6% (2,663/12,912 observations from 2,802 compounds) and was negatively associated with age (Table 1). The prevalence of antibodies to *P*. *falciparum* AMA-1 or MSP-1_19_ was 55.5% (9,092/16,381 observations from 3,099 compounds) and was positively associated with age (*p <* 0.001; Table 1). Analysis of spatial clusters based on combined antibody prevalence for AMA-1 and MSP-1_19_ and age-adjusted antibody density identified 27 statistically significant hotspots of variable size (0.035–4.5 km^2^; Fig 2B). These hotspots covered 34.7% of the total field area and 44.4% (7,780/17,503) of all sampled individuals. From the identified 27 hotspots, we selected ten based on the inclusion criteria of the presence of an evaluation zone (1–500 m from the hotspot boundary) that was ≥1 km away from neighboring hotspots, as shown in Fig 2C. Malaria antibody prevalence for AMA-1 or MSP-1_19_ was higher in nPCR parasite-positive compared to -negative individuals (odds ratio 1.94, 95% CI 1.77–2.13, *p <* 0.001). Antibody prevalence and nPCR parasite prevalence showed similar geographical patterns (Fig 2D), with nPCR parasite prevalence being highest in serologically defined hotspots and declining with increasing distance from the hotspot boundary (Fig 3). In all, 5.1% (126/2,493) of compounds with >3 inhabitants had nPCR parasite prevalence ≥ 80%; 67.5% (85/126) of these compounds were located inside serologically defined hotspots. Even at an altitude > 1,500 m, where overall nPCR prevalence was 15.3% (646/4,212 individuals from 861 compounds), 3.8% (29/773) of compounds with >3 inhabitants had nPCR parasite prevalence ≥ 80%, and 55.2% (16/29) of these compounds were located inside serologically defined hotspots.

**Table 1:** Parasite prevalence and antibody prevalence in relation to age in the cross-sectional community survey in Rachuonyo South District in June–July 2011 that was conducted for hotspot detection.

Age	nPCR Parasite Prevalence, Percent (*n/N*)	Antibody Prevalence, Percent (*n/N*)
≤5 y	21.2% (738/3,476)	33.8% (1,501/4,437)
6–10 y,	26.1% (609/2,337)	49.5% (1,483/2,994)
11–15 y,	24.8% (462/1,403)	65.1% (1,559/2,396)
16–25 y	19.2% (374/1,574)	69.5% (1,685/2,424)
>25 y	14.6% (480/3,286)	69.4% (2,864/4,130)

### Intervention Coverage and Side Effects

**Fig 4:**
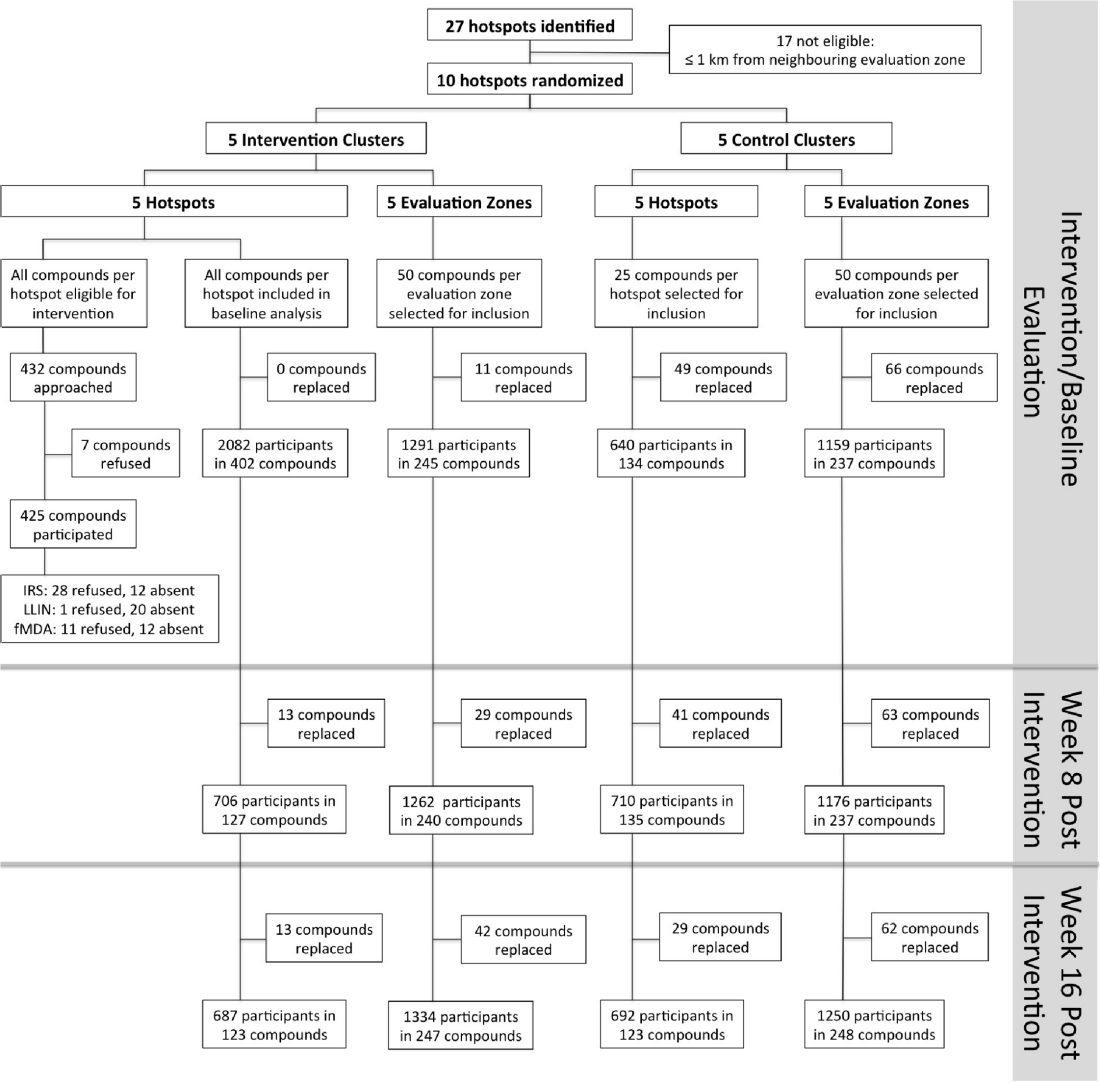
Overview of the cluster-randomized trial conducted in Rachuonyo South District in March–September 2012. Clusters were serologically defined hotspots with a surrounding 500-m evaluation zone and were randomly allocated to the intervention (*n =* 5) or control arm (*n =* 5). Cross-sectional surveys were conducted at baseline (22 March–15 April 2012) and at 8 wk (16 June–6 July 2012) and 16 wk after the intervention (21 August–10 September 2012). In each survey, 25 compounds were randomly selected from within hotspots and 50 from the surrounding evaluation zone (25 compounds 1–249 m from the hotspot boundary and 25 compounds 250–500 m from the hotspot boundary). In intervention hotspots, data were available for all compounds at baseline and were therefore included in the analysis. If selected compounds were not inhabited or compound members were absent, the nearest non-selected inhabited compound was selected as a replacement. Compounds were not revisited before replacements were sought. fMDA, focal MDA.

In intervention hotspots, seven of the 432 compounds approached were either unoccupied at the time of survey or declined participation and therefore did not participate in any of the interventions (Fig 4). Larviciding of all water bodies continued for 16 wk after the start of the intervention until the week after the final evaluation survey. LLIN distribution, IRS, and focal MDA were implemented during a period of 3 wk, 22 March–15 April 2012. The percentage of compounds that received PermaNet W 3.0 LLINs ranged from 94.5% (120/127) to 98.3% (58/59) of compounds across intervention clusters, and between 89.2% (379/425) and 97.5% (154/158) of sleeping spaces received IRS in the intervention clusters (Table 2). Of those compounds that received IRS under the normal Kenyan national policy campaign (i.e., in control clusters), 62.8% (59/94) to 81.4% (79/97) of sleeping spaces were sprayed, as reported during the 8-wk post-intervention survey (June–July 2012). In the intervention hotspots, 11.8% of compounds (cluster range 9.0% [11/120] to 16.2% [7/42]) required more nets due to missing or damaged LLINs, and there were no reported adverse effects associated with LLIN use. Ten individuals (age range 2–62 y) residing in three compounds in the intervention hotspots reported adverse effects to IRS. Reported symptoms included mild skin rashes and itching and one report of swollen and reddish mouth. All participants were provided treatment at Rachuonyo District Hospital; these were the only identified safety issues associated with the interventions. Of all compounds in the intervention hotspots, 94.0% (406/432 compounds) participated in screening of the sentinel population; on average 42.3% of the screened compounds (range 14.0% [8/57 compounds] to 66.1% [72/109 compounds]) had ≥1 RDT-positive member and were eligible for focal MDA. 94.9% (1,015/1,070) of inhabitants of these compounds completed treatment, with loss to follow-up accounting for the majority of non-compliance. There were no reports of side effects deemed likely to be related to treatment. Acceptability of interventions was not formally assessed in interviews or questionnaires; all households in intervention clusters were visited as part of the LLIN follow-up visits at 8-wk post-intervention to record any side effects or complaints. The high coverage levels achieved indicate a high degree of approval by the communities.

**Table 2:** Cluster-randomized trial baseline characteristics (March–April 2012) and intervention coverage within control and intervention hotspots in Rachuonyo South District.

Characteristic	Intervention Hotspots						Control Hotspots					
	Hotspot 4	Hotspot 6	Hotspot 7	Hotspot 8	Hotspot 10	Average	Hotspot 1	Hotspot 2	Hotspot 3	Hotspot 5	Hotspot 9	Average
**Number of compounds (inhabitants) sampled**	120 (631)	73 (365)	42 (233)	58 (282)	109 (571)	80 (416)	30 (135)	27 (133)	26 (119)	25 (122)	26 (131)	27 (128)
**Altitude range (meters)**	1,396–1,424	1,475–1,499	1,450–1,474	1,500+	1,425–1,449	1,450–1,474	1,350–1,449	1,350–1,449	1,500–1,549	1,450–1,499	1,500–1,549	1,450–1,499
**Average distance to water (meters)**	403	239	239	398	257	316	448	271	618	569	510	479
**Parasite prevalence by nPCR, percent (*n/N*)**	19.3 (122/630)	20.1 (73/364)	16.8 (39/232)	11.7 (33/ 281)	39.0 (222/570)	23.5 (489/2,077)	12.6 (17/135)	21.1 (28/133)	6.7 (8/119)	30.3 (37/122)	22.1 (29/131)	18.6 (119/640)
**Compounds that received LLINs as part of intervention, percent (*n/N*)**	94.5 (120/127)	96.2 (76/79)	97.7 (43/44)	98.3 (58/59)	96.6 (114/118)	96.3 (411/427)	—	—	—	—	—	—
**LLIN coverage**												
Use before intervention	73.1 (440/602)	71.8 (227/316)	69.3 (142/205)	53.4 (142/266)	77.4 (425/549)	71.5 (1,385/1,938)	77.1 (101/131)	75.4 (86/114)	73.0 (84/115)	82.8 (101/122)	59.4 (73/123)	73.6 (445/605)
Use after intervention	95.7 (135/141)	94.2 (129/137)	94.5 (120/127)	96.0 (119/124)	97.1 (168/173)	95.6 (671/702)	90.3 (121/134)	84.4 (108/128)	93.7 (163/174)	77.6 (97/125)	66.0 (95/144)	82.8 (584/705)
**IRS coverage (*n/N* sleeping spaces)**	90.7 (421/464)	89.8 (255/284)	97.5 (154/158)	93.4 (198/212)	89.2 (379/425)	91.2 (1,407/1,543)	74.1 (80/108)	81.4 (79/97)	67.0 (63/94)	70.0 (63/90)	62.8 (59/94)	71.2 (344/483)
**Focal MDA coverage**												
Compounds participating in screening, percent (*n/N*)	93.8 (121/129)	93.7 (74/79)	100 (44/44)	96.7 (58/60)	90.8 (109/120)	94.0 (406/432)	—	—	—	—	—	—
Compounds with ≥1 RDT-positive inhabitant, percent (*n/N*)	42.1 (51/121)	34.2 (25/73)	33.3 (14/42)	14.0 (8/57)	66.1 (72/109)	42.3 (170/402)	—	—	—	—	—	—
Members of eligible compounds who completed treatment, percent (*n/N*)	91.5 (301/329)	97.5 (155/159)	91.3 (94/103)	95.8 (46/48)	97.2 (419/431)	94.9 (1,015/1,070)	—	—	—	—	—	—

Coverage with LLINs was defined as percentage of respondents reporting sleeping under a LLIN the night before the survey. Coverage of IRS was defined as the proportion of successfully sprayed sleeping spaces and was assessed in all compounds in the intervention hotspots and a random selection of compounds in control hotspots during the June–July 2012 survey. Coverage with the focal MDA campaign was defined as the proportion of compounds that participated in the screening of sentinel populations by RDT that prompted treatment, the proportion of compounds with ≥1 RDT-positive inhabitant that were therefore eligible for MDA, and the proportion of the members of these eligible compounds who completed six doses of treatment, of which the morning doses (dose 1, 3, and 5) were observed and the afternoon doses were given without supervision but blisters were checked.

### Effect of Hotspot-Targeted Interventions on nPCR Parasite Prevalence in Evaluation Zones

Parasite prevalence in the malaria hotspots and in the evaluation zones surrounding the malaria hotspots was determined based on nPCR parasite detection in three surveys conducted in March–April (baseline), June–July (8 wk post-intervention), and August–September 2012 (16 wk post-intervention). Each survey took approximately 2 wk to complete. Mean baseline nPCR parasite prevalence was 20.1% (range 8.4% [8/95] to 38.4% [73/190]) in the intervention hotspots and 18.6% (range 6.7% [8/119] to 30.3% [37/122]) in the control hotspots (Table 3); all compounds in the intervention clusters were sampled at baseline to facilitate evaluation of the focal treatment campaigns, and all were included in the baseline survey [23]. At none of the time points of evaluation did we observe a statistically significant reduction in nPCR parasite prevalence in the evaluation zones surrounding targeted hotspots (Table 3; *p* ≥ 0.19). Hotspot-specific data for nPCR prevalence for each survey are presented in S1 Fig and S1 Table.

**Table 3:** The impact of the combined targeted interventions on nPCR malaria parasite prevalence, complexity of infection, and allelic richness in hotspots and evaluation zones.

Time Point	Group or Value	nPCR Parasite Prevalence (95% CI)			Mean Number of Clones (95% CI)*			Mean Complexity of Infection (95% CI)^¥^			Mean Allelic Richness		
		Hotspot	Evaluation Zone		Hotspot	Evaluation Zone		Hotspot	Evaluation Zone		Hotspot	Evaluation Zone	
			1–249 m	250–500 m		1–249 m	250–500 m		1–249 m	250–500 m		1–249 m	250–500 m
**Baseline(2012)**	Intervention	20.1 (6.3 to 33.9)	13.2 (7.1 to 19.4)	13.5 (7.6 to 19.4)	0.45 (0.04 to 0.85)	0.28 (0.14 to 0.42)	0.30 (0.15 to 0.44)	2.12 (1.65 to 2.60)	2.15 (1.67 to 2.63)	2.24 (1.14 to 3.33)	16.3	18.3	12.4
	Control	18.6 (7.2 to 29.9)	11.7 (6.0 to 17.4)	16.9 (2.3 to 31.5)	0.45 (0.22 to 0.68)	0.28 (0.16 to 0.39)	0.43 (0.08 to 0.78)	2.64 (1.75 to 3.52)	2.50 (2.08 to 2.93)	2.74 (1.72 to 3.76)	17.0	16.3	11.4
**8 wk post-intervention**	Intervention	9.2 (4.4 to 14.0)	12.3 (9.8 to 14.8)	9.1 (4.3 to 13.9)	0.24 (0.11 to 0.36)	0.30 (0.14 to 0.45)	0.29 (0.05 to 0.53)	2.70 (2.04 to 3.35)	2.46 (1.81 to 3.12)	2.98 (0.73 to 5.24)	15.4	18.0	6.1
	Control	19.4 (6.5 to 32.3)	15.9 (8.9 to 22.9)	12.9 (7.1 0 18.7)	0.41 (0.00 to 0.85)	0.35 (0.16 to 0.54)	0.30 (0.17 to 0.43)	2.08 (1.39 to 2.76)	2.45 (2.08 to 2.83)	2.34 (1.60 to 3.09)	18.6	20.2	6.8
	Difference	10.2 (−1.3 to 21.7)	3.6 (−2.6 to 9.7)	3.8 (−2.4 to 10.0)	0.17 (−0.19 to 0.55)	0.05 (−0.15 to 0.26)	0.01 (−0.21 to 0.23)	−0.62 (−1.41 to 0.17)	−0.01 (−0.64 to 0.62)	−0.64 (−2.61 to 1.33)			
	*p-*Value	0.075	0.219	0.199	0.307	0.560	0.923	0.110	0.968	0.475	0.230	0.202	0.239
	Adjusted *p*-value^§^	0.024	0.216	0.187	0.170	0.530	0.792	0.058	0.962	0.552			
**16 wk post-intervention**	Intervention	10.1 (3.2 to 17.1)	11.4 (2.6 to 20.1)	9.8 (2.6 to 17.1)	0.19 (0.02 to 0.35)	0.26 (0.04 to 0.49)	0.23 (0.13 to 0.33)	1.76 (1.12 to 2.40)	2.33 (1.78 to 2.88)	2.57 (1.48 to 3.76)	11.5	19.7	10.5
	Control	14.5 (5.4 to 23.5)	12.4 (8.2 to 16.6)	10.8 (2.2 to 19.5)	0.29 (0.13 to 0.46)	0.27 (0.16 to 0.37)	0.24 (0.00 to 0.49)	2.06 (1.67 to 2.45)	2.13 (1.92 to 2.34)	2.06 (1.59 to 2.52)	12.9	18.9	10.3
	Difference	4.2 (−5.1 to 13.8)	1.0 (−7.0 to 9.1)	1.0 (−8.3 to 10.4)	0.11 (−0.09 to 0.30)	0.00 (−0.20 to 0.21)	0.01 (−0.22 to 0.23)	0.30 (−0.32 to 0.92)	−0.20 (−0.69 to 0.29)	−0.52 (−1.51 to 0.47)			
	*p*-Value	0.326	0.775	0.804	0.248	0.980	0.933	0.274	0.380	0.261	0.217	0.679	0.844
	Adjusted *p*-value^§^	0.265	0.713	0.809	0.375	0.990	0.625	0.375	0.615	0.379			

At baseline (March 2012), the standard deviation of nPCR prevalence in hotspots was 9.6, and the observed coefficient of variation was therefore 0.50.

*Including all nPCR-positive and -negative results.

^¥^Including only nPCR-positive individuals.

^§^Adjusted for baseline malaria prevalence, age, sex, altitude, and living in a house with open eaves.

### Effect of Hotspot-Targeted Interventions on nPCR Parasite Prevalence in Hotspots and Parasite Complexity of Infection

Secondary parasitological outcomes were nPCR parasite prevalence inside hotspots and nPCR parasite prevalence in the evaluation zone as a function of distance from the hotspot boundary. An exploratory parasitological endpoint was the complexity of malaria infections inside and outside targeted hotspots. In the first post-intervention cross-sectional survey (at 8 wk), mean nPCR parasite prevalence was 9.2% in intervention hotspots (cluster range 5.1% [7/138] to 13.5% [17/126]) compared to 19.4% in control hotspots (cluster range 9.6% [12/125] to 33.3% [42/126]) (Table 3; *p =* 0.024 after adjustment for baseline prevalence and covariates). However, malaria transmission inside targeted hotspots was not completely interrupted. Although not originally defined as a study endpoint, we determined parasite carriage in individuals who were repeatedly sampled during the study period and were parasite-free by nPCR before the intervention. Of 97 individuals who resided in intervention hotspots, were parasite negative prior to the intervention, and were coincidentally sampled during the first evaluation survey, four (4.1%) became parasite positive. These individuals (aged 4, 6, 10, and 34 y) did not report spending any nights outside their compound between surveys. Sixteen weeks post-intervention, at the end of the transmission season, there was no longer a statistically significant difference in nPCR parasite prevalence between intervention and control hotspots (*p =* 0.27). We observed no statistically significant trend in the effect of the intervention on nPCR parasite prevalence in the evaluation zone in relation to distance from the hotspot boundary 8 wk post-intervention (*p =* 0.27) or 16 wk post-intervention (*p =* 0.75). nPCR parasite prevalence was not statistically significantly associated with reported travel in the preceding 3 mo in the survey prior to the intervention (*p =* 0.30) or the surveys 8 wk (*p =* 0.24) and 16 wk (*p =* 0.58) after the intervention. Only one nPCR-positive individual reported traveling to an area that is considered to experience higher malaria transmission. MSP-2 typing and fragment sizing was successful in 97.2% (1,517/1,561) of nPCR-positive individuals coming from 421 compounds. We observed no statistically significant reductions in allelic richness or in the number of detected parasite clones inside malaria hotspots or in evaluation zones following the intervention (Table 3). The average number of parasite clones, complexity of infection, and allelic richness in the different surveys is presented by hotspot in S1 Table.

### Effect of Hotspot-Targeted Interventions on Malaria Cases Reporting at Health Facilities

The number of malaria cases reporting at health facilities coming from intervention and control clusters was defined as a secondary endpoint. During PCD there were 561 RDT-confirmed clinical malaria cases in 1,175 febrile patients. The majority resided outside intervention or control clusters. Twenty-two patients with RDT-confirmed clinical malaria could be located to intervention clusters (incidence of 0.74 cases per 1,000 people per month based on an estimated population size of 4,918) and 14 to control clusters (incidence of 0.64 cases per 1,000 people per month based on an estimated population size of 3,660).

### Effect of Hotspot-Targeted Interventions on Number of Mosquitoes

**Fig 5:**
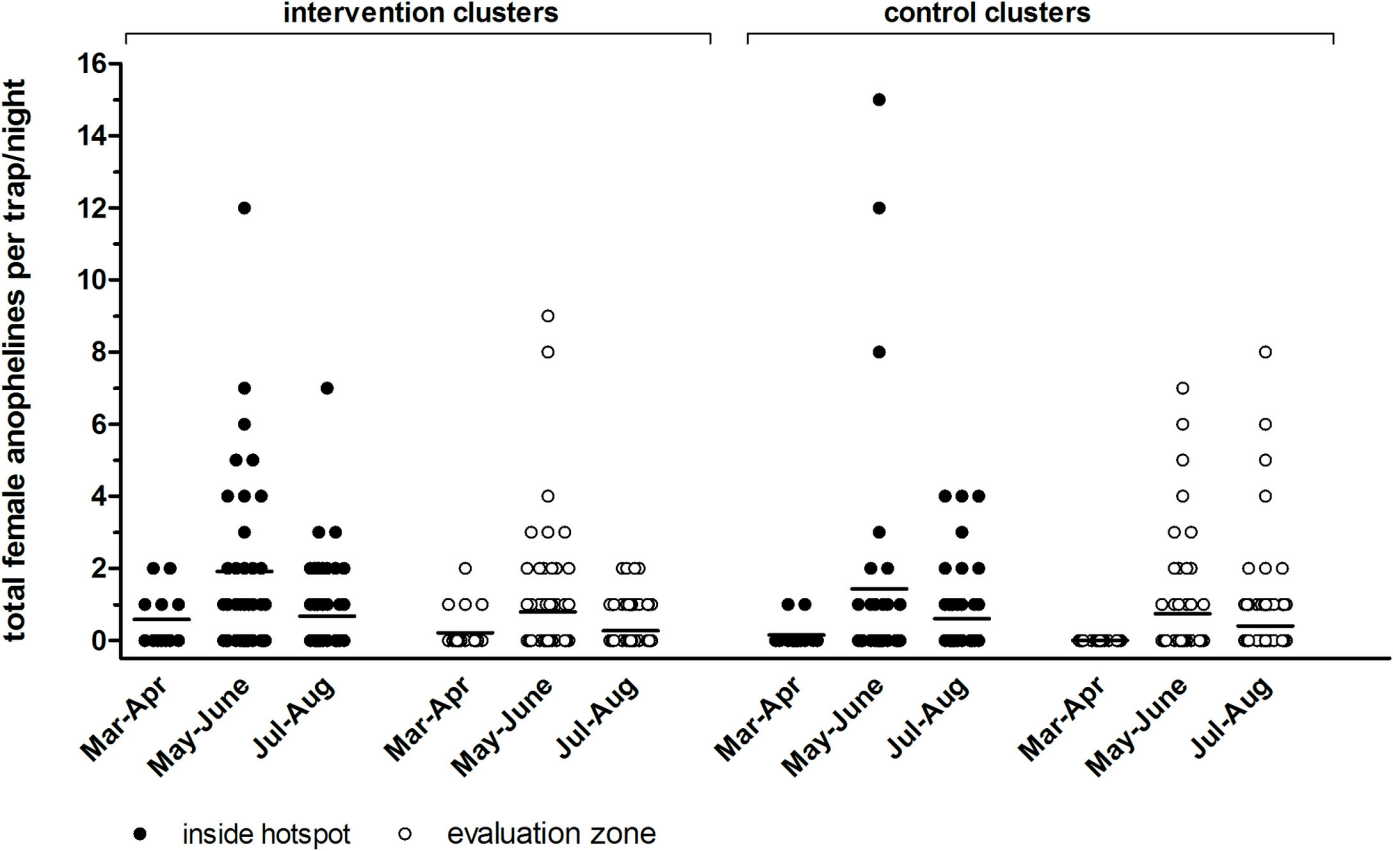
Indoor densities of female anophelines by light trap in intervention and control clusters in Rachuonyo South District in March–August 2012. Each symbol represents the number of female anophelines caught indoors by CDC light trap inside hotspots (filled circles) and in evaluation zones (open circles). Each trap night, four compounds were randomly selected within the hotspot and eight were selected in the evaluation zone per cluster. Findings are summarized for trapping rounds prior to roll-out of interventions in 22 March–30 April 2012 (one trapping night per compound) and post-intervention in 1 May–30 June 2012 (three trapping nights per compound) and 1 July–31 August 2012 (five trapping nights per compound). Findings are presented for three intervention clusters combined and for three control clusters combined.

*Anopheles* mosquito density and breeding site productivity were defined as secondary entomological objectives. We sampled a total of 395 female anophelines during 648 trapping nights. In the intervention clusters we caught an average of 1.14 female anophelines inside hotspots and 0.47 in evaluation zones; in control clusters we caught an average of 0.90 female anophelines inside hotspots and 0.50 in evaluation zones. We observed no apparent difference between intervention and control clusters (Fig 5) and considerable variation within and between clusters (S2 Fig). Mosquito breeding site productivity was assessed in 15 sites per intervention hotspot (*n =* 75 in total). Of the sites sampled prior to larviciding, 45% (34/75) were positive, of which 12 had late-stage larvae and/or pupae. After larviciding, the number of positive sites varied from 5.3% (4/75) to 28.0% (21/75), and no late-stage larvae or pupae were detected.

## Discussion

In this highland fringe setting in western Kenya, we identified numerous malaria hotspots with serological and parasitological evidence of elevated levels of malaria transmission relative to surrounding areas. Targeting these hotspots with a combination of four interventions, acting against the human parasitemic reservoir and vector populations, had no measureable impact on nPCR parasite prevalence in evaluation zones surrounding the hotspots and had only a short-lived effect on nPCR parasite prevalence within hotspots.

Hotspot-targeted interventions have been hypothesized to form a highly efficacious approach to reduce the burden of malaria in areas of heterogeneous malaria transmission [9,12]. Operationally feasible approaches to detect stable hotspots form a prerequisite for targeted interventions. Our detailed community survey, conducted in preparation for the intervention, revealed a large number of serologically defined hotspots of malaria transmission. These serological hotspots, indicative of longer-term exposure [17], showed a strong association with current parasite carriage at both the individual and geographical level: malaria-antibody-positive individuals were significantly more likely to be parasite positive, and nPCR parasite prevalence decreased with distance from the center of serologically defined hotspots. This suggests temporal stability of spatially heterogeneous transmission [6,9] and the suitability of targeting interventions at a fine spatial scale. We selected ten hotspots for a cluster-randomized trial on the impact of hotspot-targeted interventions with IRS, LLINs, weekly larviciding [31], and focal MDA to minimize the parasite reservoir in humans at the start of the transmission season. Despite achieving high coverage with all interventions, we observed no measurable impact on our primary endpoint, the proportion of nPCR parasite-positive individuals in the evaluation zone surrounding targeted hotspots. nPCR parasite prevalence was only transiently reduced inside targeted hotspots, and there was no impact on the complexity of malaria infections inside or outside hotspots. Whilst heterogeneous malaria transmission may influence the acquisition of malaria immunity and clinical manifestations [6,32], we hypothesized that our intervention would reduce not only the prevalence of asymptomatic infections but also the incidence of clinical malaria episodes in intervention compared to control clusters. PCD at health facilities identified 36 RDT-confirmed clinical malaria episodes in individuals who resided in intervention or control clusters, and no evidence that the intervention reduced the clinical burden of malaria. Our decision to monitor the incidence of malaria cases passively was based on the low efficiency of active monitoring of infections in low endemic settings [33], although we acknowledge that PCD leads to a considerable loss in power compared to active case detection [34]. As a consequence, our approach will have resulted in an unknown number of malaria episodes that were missed due to health care seeking behavior or other factors [34,35]. It is also conceivable that we missed further malaria cases because health facility attendees failed to present with compound identification cards, although we would expect this bias to be non-differential between the control and intervention arms. These are shortcomings of our PCD system. As a result, we cannot exclude an impact of our interventions on the incidence of clinical malaria episodes but can conclude that parasitologically confirmed clinical malaria episodes were passively reported from both intervention and control clusters at a similar rate.

Our intervention failed to sustainably reduce malaria transmission inside targeted hotspots. Approximately 4% of individuals in intervention hotspots became parasite positive within 8 wk of the intervention. This infection incidence is remarkable considering the low level of transmission intensity in the area; the baseline nPCR parasite prevalence of 18.6%–23.5% translates to an estimated microscopy parasite prevalence of 5.7%–7.9% [36] and indicates hypoendemic transmission [37]. There are several possible reasons for the apparent failure to completely eliminate malaria transmission inside hotspots and the undetectable impact in the evaluation zones surrounding targeted hotspots. First, it is possible that our interventions did not clear vector populations or prevent human–vector contact inside hotspots to the extent that is required to interrupt local transmission. Insecticide resistance is a key consideration for any vector-based malaria intervention. Resistance to deltamethrin and permethrin has been detected in the study area for *A*. *gambiae* s.l. and *A*. *funestus* [38,39] but has not reached saturation, as observed in other African settings [40,41], and there is currently no evidence that insecticide resistance affects control efforts in the region [40–42]. We purposefully selected interventions that have potency in areas of insecticide resistance: LLINs that include piperonyl butoxide as a syngergist to enhance the efficacy of deltamethrin in resistant vectors [43] and larviciding with *B*. *thuringiensis* var. *israelensis*, to which there is no reported vector resistance in the region [22] and which is effective against both indoor- and outdoor-feeding mosquitoes. Activity against outdoor-feeding mosquitoes is of relevance because of increased outdoor biting rates following the scaling up of IRS and LLIN coverage [44,45] and the presence of a previously unidentified malaria vector in the study area that exhibits a preference for outdoor biting [16]. Despite our intensive vector control efforts, we detected ongoing breeding site productivity and mosquito exposure in targeted hotspots. Second, there may have been residual transmission from parasite carriers who were unidentified and untargeted by our treatment campaign. Undetected low-density parasite carriage may have been responsible for sustained transmission following recent mass screening and treatment campaigns in Zanzibar [46]. We estimate that our approach identified approximately 77% of all nPCR-positive individuals in the area [23]. It is currently unknown whether the untargeted fraction of infections, which are mostly of low parasite density, is sufficient to sustain transmission; however, infectiousness from low-density infections has been demonstrated [47]. A third potential explanation for the apparent failure to completely eliminate malaria transmission is the importation of parasites by individuals traveling to areas of higher endemicity such as the nearby Asembo Bay area [48]. We failed to detect an association between reported travel and nPCR parasite prevalence during any of the surveys. Analysis of data from earlier surveys in this area indicates that most trips that involve an overnight stay occur within families or represent travel to urban areas where malaria risk is low [49]. In our surveys, only one nPCR-positive individual reported traveling to an area of known intense malaria transmission. A fourth possible explanation is the influx of infected mosquitoes from untargeted hotspots. We selected hotspots and evaluation zones for the intervention trial that were in relative isolation from neighboring hotspots, assuming that vector dispersion in a densely populated area would primarily occur over <1 km distances [50]. Importation of infected mosquitoes into the intervention hotspots is possible, most likely from areas that surrounded hotspots but were themselves not detected as transmission hotspots. The points above may all contribute to varying degrees and combine for the fifth possible explanation, which is that our hypothesis of how hotspots seed malaria transmission does not apply to the transmission dynamics in our study setting. Understanding host and mosquito movement in relation to fine-scale patterns of mixing parasite populations is of key importance for rationally deploying targeted interventions [51]. Our findings suggest that transmission may not primarily occur from hotspots to the surrounding areas. Our hotspot definition encapsulated 34.7% of the total area and 44.4% of all inhabitants, but 32.5% (41/126) of all compounds with ≥80% nPCR parasite prevalence were located outside statistically significant malaria hotspots. These high parasite prevalence compounds may have fueled transmission outside and into our serologically defined hotspots. The existence of isolated high parasite prevalence compounds has been described before [12,23] and suggests that our hypoendemic study area has not yet reached the phase where parasite carriage is confined to malaria hotspots or primarily occurs from hotspots to surrounding areas.

There are several limitations to this study. Our study was conducted in the presence of other ongoing interventions that may have made it difficult to attribute any specific, small changes in nPCR parasite prevalence to our intervention. The absence of detailed concurrent meteorological data makes it impossible to assess the impact of our interventions in terms of the exact timing of the local changes in rainfall and temperature that determine malaria transmission dynamics. Our trial included a total of ten clusters during a single season and was therefore not powered to detect subtle effects of hotspot-targeted interventions nor designed to detect effects of interventions that become apparent over multiple transmission seasons. Furthermore, we saw a higher than expected level of inter-cluster variation. Sample size calculations were based on an assumed coefficient of variation of 0.4. In fact, the baseline data indicate a coefficient of variation of 0.5, which would have also reduced the power of the trial to detect subtle effects of hotspot-targeted interventions. The limited number of clusters is a major limitation of the current study. Our sample size of five intervention and five control clusters was based on the assumption that our combination of four malaria interventions would (temporarily) eliminate malaria transmission inside targeted hotspots [9] and would result in near elimination of malaria from the surrounding community. Neither hypothesis was confirmed. It is unclear to what extent the results from our trial can be extrapolated to other settings. Our study was conducted in a highland fringe setting with low and heterogeneous malaria transmission. Whilst this setting is markedly different from nearby lowland areas of intense malaria transmission, we believe our study setting is representative of many other East African settings with continuous habitation and low-intensity malaria transmission that is spatially heterogeneous as a consequence of large- and small-scale determinants of malaria transmission [9,12]. Our observation that individual compounds with high levels of asymptomatic parasite carriage exist within areas where parasite carriage is generally much lower has also been reported in coastal Kenya [12]. We consider the existence of such single-compound hotspots an important hurdle for hotspot-targeted interventions since these compounds are logistically very challenging to identify and may require very intensive community surveys. In coastal Kenya, hotspots that are unstable in space and time [6] may further affect the operational attractiveness of hotspot-targeted interventions.

### Conclusion

Approximately one-third of our study area fell within identified hotspots of malaria transmission. Hotspot targeting of interventions failed to influence malaria transmission dynamics outside the targeted area and resulted in a modest and transient reduction in nPCR parasite prevalence inside targeted hotspots. Whilst our study may have been underpowered to detect subtle effects on malaria transmission, we consider it unlikely that hotspot-targeted interventions are cost-effective in our setting. The considerable resources required for hotspot detection at the local scale are unlikely to be offset by savings from a more rational deployment of interventions in the study area. As a result, areas like our study site in Rachuonyo South District, with highly heterogeneous but widespread malaria transmission, may currently benefit most from an untargeted community-wide approach that reaches all malaria-infected and malaria-exposed individuals. The hotspot-targeted approach may have validity and should be tested further in settings where human settlement is more nuclear.

## Supporting Information

S1 Fig Parasite prevalence before and after hotspot-targeted interventions in Rachuonyo South District in March–August 2012. nPCR prevalence (percent) in hotspots selected for the cluster-randomized trial, measured in July 2011 (community survey for hotspot detection), pre-intervention in March–April 2012 and post-intervention June–July 2012 and August–September 2012. Measured inside hotspot, 1–249 m from hotspot border and 250–500 m from hotspot border. Error bars indicate the upper limit of the 95% confidence interval.
(DOCX)

S2 Fig Indoor densities of female anophelines by light trap in intervention and control clusters in Rachuonyo South District in March–August 2012, presented for individual clusters. Each symbol represents the number of female anophelines caught indoors by CDC light trap inside hotspots (filled circles) and in evaluation zones (open circles). Each trap night, four compounds were randomly selected within the hotspot and eight were selected in the evaluation zone per cluster. Findings are summarized for trapping rounds prior to roll-out of interventions in March–April (one trapping night per compound) and post-intervention in May–June (three trapping nights per compound) and July–August (five trapping nights per compound).
(DOCX)

S1 Table The impact of the combined targeted interventions on malaria prevalence and complexity of infection inside hotspots of malaria transmission in Rachuonyo South District in March–September 2012, presented per hotspot. (DOCX)

S1 Text Trial protocol. (DOCX)

S2 Text CONSORT statement. (DOC)

## Abbreviations

AL: artemether-lumefantrine
CDC: Centers for Disease Control and Prevention
IQR: interquartile range
IRS: indoor residual spraying
ITN: insecticide-treated net
KEMRI: Kenya Medical Research Institute
LLIN: long-lasting insecticide-treated net
MDA: mass drug administration
nPCR: nested PCR
PCD: passive case detection
RDT: rapid diagnostic test
