# Supplementary material for: The Impact of Hotspot-Targeted Interventions on Malaria Transmission in Rachuonyo South District in the Western Kenyan Highlands: A Cluster-Randomized Controlled Trial
Source: PLoS Med. 2016 Apr 12;13(4):e1001993. doi: 10.1371/journal.pmed.1001993 (PMC4829260; doi:10.1371/journal.pmed.1001993)
Supplement: S1 Fig — nPCR prevalence (percent) in hotspots selected for the cluster-randomized trial, measured in July 2011 (community survey for hotspot detection), pre-intervention in March–April 2012 and post-intervention June–July 2012 and August–September 2012. Measured inside hotspot, 1–249 m from hotspot border and 250–500 m from hotspot border. Error bars indicate the upper limit of the 95% confidence interval. (DOCX) [file pmed.1001993.s001.docx]

**S1 Fig. Parasite prevalence following before and after hotspot-targeted interventions in Rachuonyo South District in March-August 2012.** nPCR prevalence (%) in hotspots selected for the cluster randomized trial; measured in July 2011 (community survey for hotspot detection), March-April 2012 (pre-intervention) and post intervention June 2012, August 2012. Measured inside hotspot, 1-249 m from hotspot border and 250-500 m from hotspot border. Error bars indicate the upper limit of the 95% confidence interval.


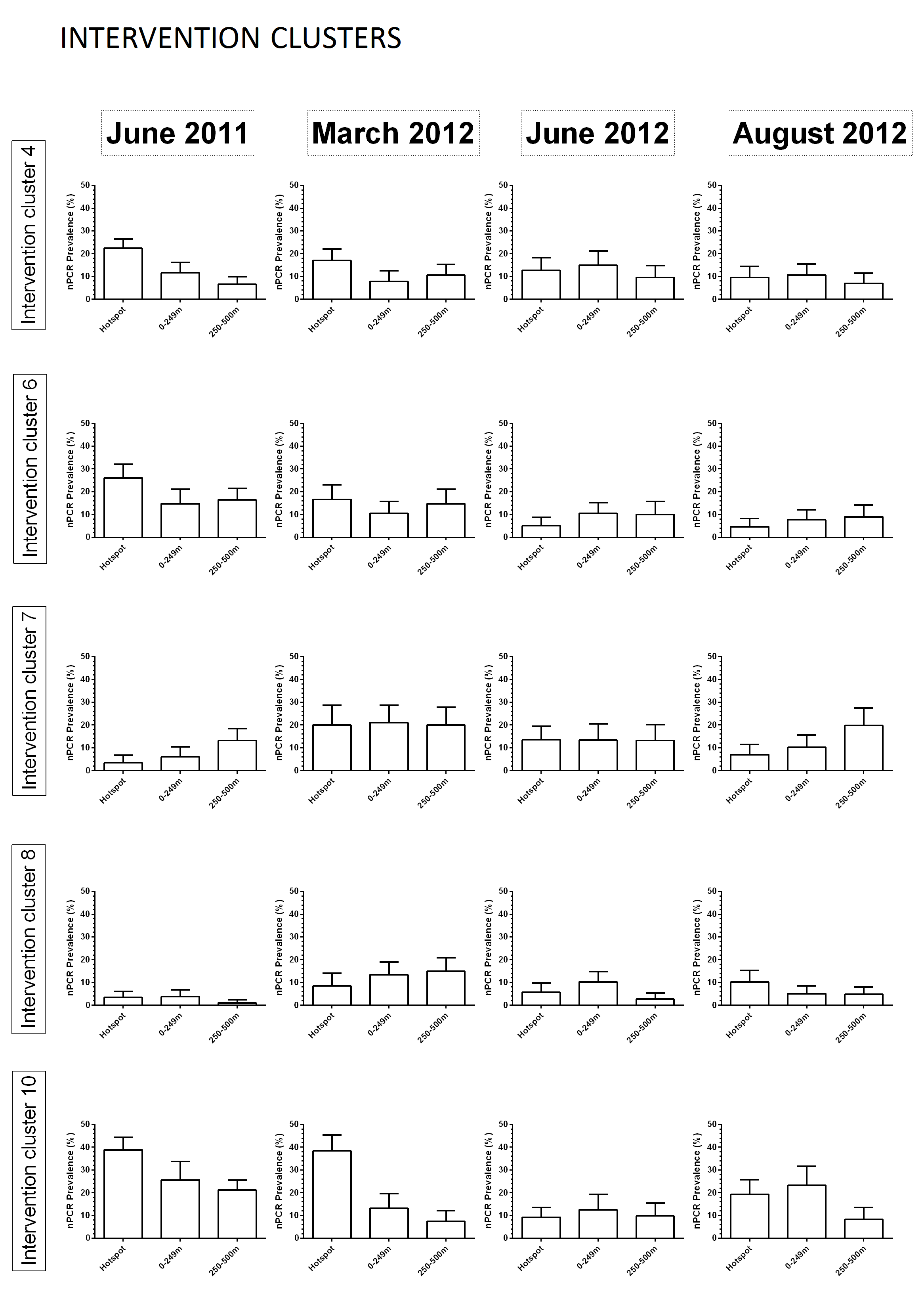


**
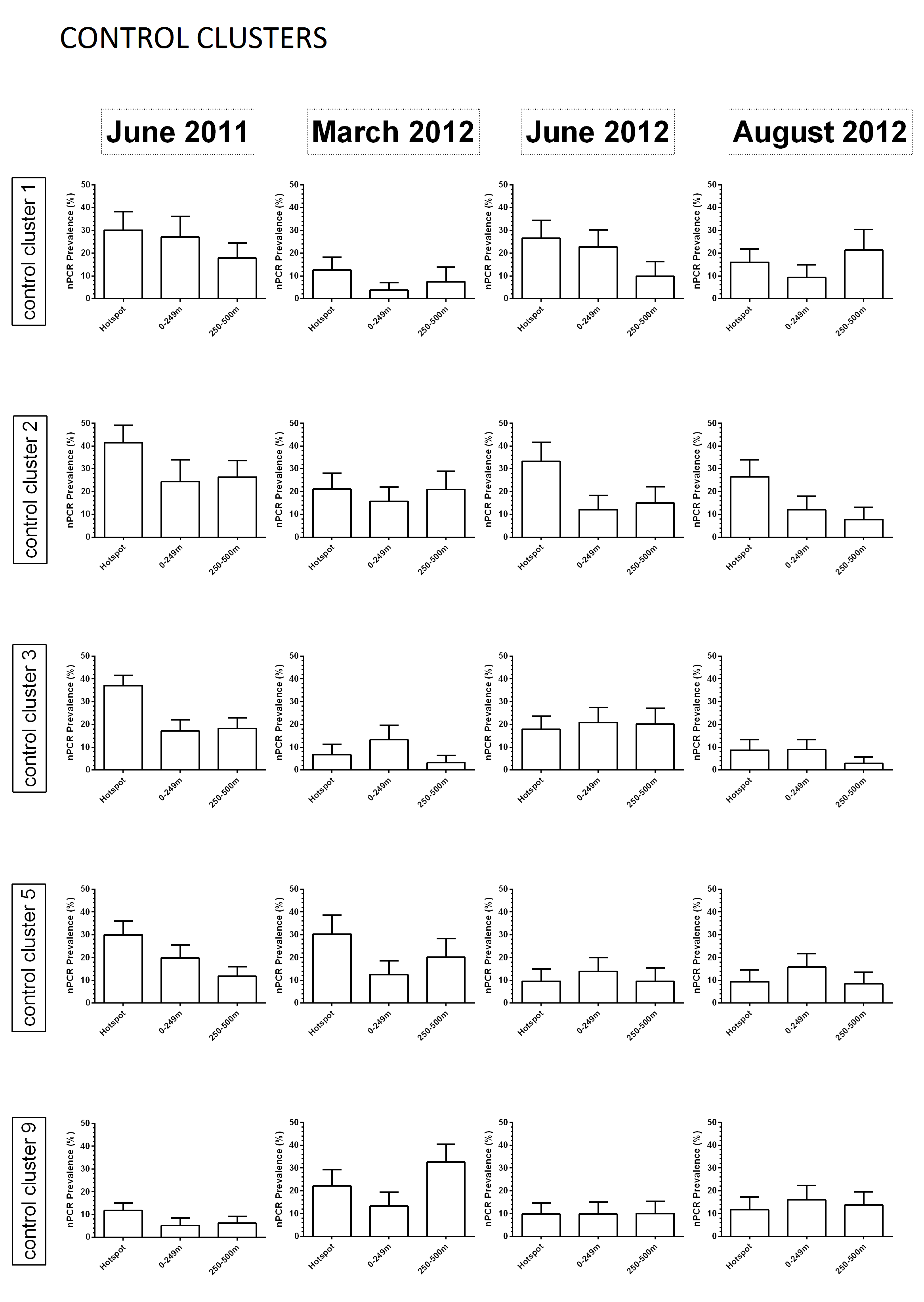
**
