# Supplementary material for: The Impact of Hotspot-Targeted Interventions on Malaria Transmission in Rachuonyo South District in the Western Kenyan Highlands: A Cluster-Randomized Controlled Trial
Source: PLoS Med. 2016 Apr 12;13(4):e1001993. doi: 10.1371/journal.pmed.1001993 (PMC4829260; doi:10.1371/journal.pmed.1001993)
Supplement: S2 Fig — Each symbol represents the number of female anophelines caught indoors by CDC light trap inside hotspots (filled circles) and in evaluation zones (open circles). Each trap night, four compounds were randomly selected within the hotspot and eight were selected in the evaluation zone per cluster. Findings are summarized for trapping rounds prior to roll-out of interventions in March–April (one trapping night per compound) and post-intervention in May–June (three trapping nights per compound) and July–August (five trapping nights per compound). (DOCX) [file pmed.1001993.s002.docx]

**S2 Fig. Indoor densities of female anophelines by light trap in intervention and control clusters** **in Rachuonyo South District in March-August 2012, presented for individual clusters**. Each symbol represents the number of female anophelines caught indoors by CDC light trap inside hotspots (filled circles) and in evaluation zones (open circles). Each trap night 4 compounds were randomly selected within the hotspot and 8 were selected in the evaluation zone per cluster. Findings are summarized for trapping rounds prior to roll-out of interventions in March-April (1 trapping night per compound), and post-intervention in May-June (3 trapping nights per compound) and July-August (5 trapping nights per compound).

**
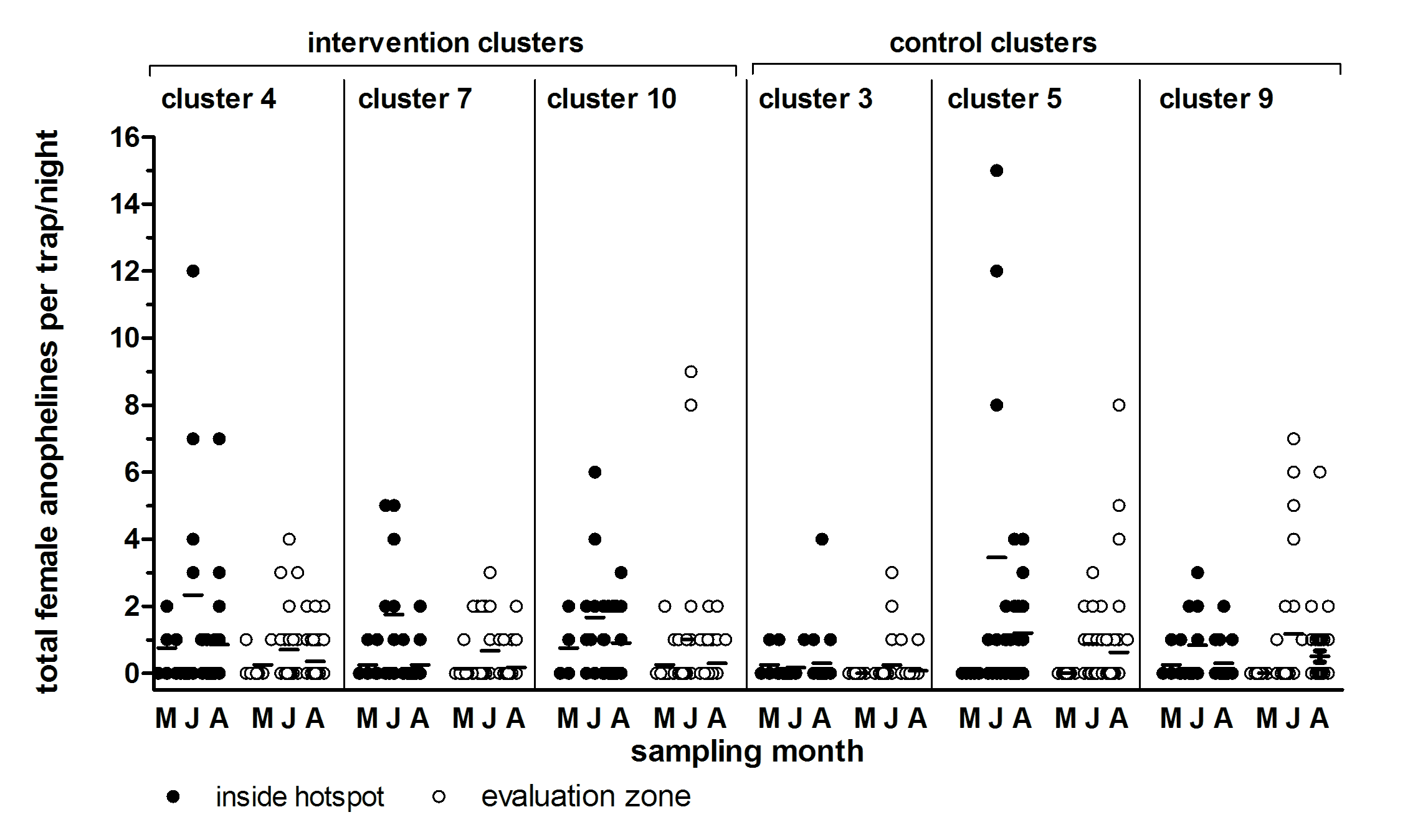
**
