# Supplementary material for: The Impact of Hotspot-Targeted Interventions on Malaria Transmission in Rachuonyo South District in the Western Kenyan Highlands: A Cluster-Randomized Controlled Trial
Source: PLoS Med. 2016 Apr 12;13(4):e1001993. doi: 10.1371/journal.pmed.1001993 (PMC4829260; doi:10.1371/journal.pmed.1001993)
Supplement: S1 Table — (DOCX) [file pmed.1001993.s003.docx]

**S1 Table. The impact of the combined targeted interventions on malaria prevalence and complexity of infection inside hotspots of malaria transmission** **in Rachuonyo South District in March-August 2012, presentation per hotspot**

| Cluster number | Baseline | | | | 1^st^ evaluation (8 weeks post intervention) | | | | 2^nd^ evaluation (16 weeks post intervention) | | | |
| --- | --- | --- | --- | --- | --- | --- | --- | --- | --- | --- | --- | --- |
|  | Parasite prevalence | Average number of parasite clones | | Allelic richness | Parasite prevalence | Average number of parasite clones | | Allelic richness | Parasite prevalence | Average number of parasite clones | | Allelic richness |
|  |  | Mean number of clones (Including nPCR negative individuals) | Complexity of infection (Excluding nPCR negative individuals) |  |  | Mean number of clones (Including nPCR negative individuals) | Complexity of infection (Excluding nPCR negative individuals) |  |  | Mean number of clones (Including nPCR negative individuals)) | Complexity of infection (Excluding nPCR negative individuals) |  |
| 4 | 17.1% (217) | 0.35 | 2.02 | 18.7 | 12.7% (142) | 0.38 | 3.11 | 20.0 | 9.6% (146) | 0.15 | 1.57 | 12.2 |
| 6 | 16.7% (132) | 0.33 | 2.15 | 17.5 | 5.1% (138) | 0.13 | 2.57 | 11.0 | 4.7% (129) | 0.09 | 1.83 | 10.7 |
| 7 | 20.0% (85) | 0.42 | 2.12 | 14.8 | 13.5% (126) | 0.24 | 1.88 | 16.1 | 7.0% (128) | 0.07 | 1.00 | 8.0 |
| 8 | 8.4% (95) | 0.14 | 1.63 | 10.0 | 5.7% (124) | 0.15 | 2.71 | 12.0 | 10.2% (137) | 0.24 | 2.24 | 13.4 |
| 10 | 38.4% (190) | 1.00 | 2.70 | 20.4 | 9.3% (173) | 0.28 | 3.20 | 18.1 | 19.3% (145) | 0.39 | 2.03 | 13.4 |
| Total | 21.8% (719) | 0.50 | 2.34 | 16.3 | 9.3% (703) | 0.24 | 2.71 | 15.4 | 10.4% (685) | 0.19 | 1.86 | 11.5 |
| Control |  |  |  |  |  |  |  |  |  |  |  |  |
| 1 | 12.6 % (135) | 0.28 | 2.24 | 15.8 | 26.6% (128) | 0.55 | 2.09 | 22.2 | 16.0% (150) | 0.39 | 1.44 | 12.7 |
| 2 | 21.1% (133) | 0.46 | 2.18 | 19.0 | 33.3% (126) | 0.97 | 2.95 | 23.0 | 26.5% (136) | 0.49 | 1.83 | 12.7 |
| 3 | 6.7% (119) | 0.26 | 3.88 | 17.2 | 17.9% (179) | 0.16 | 1.42 | 14.8 | 8.7% (149) | 0.21 | 2.38 | 14.0 |
| 5 | 30.3% (122) | 0.69 | 2.27 | 15.6 | 9.6% (125) | 0.19 | 2.00 | 18.1 | 9.5% (127) | 0.17 | 1.75 | 10.7 |
| 9 | 22.1% (131) | 0.58 | 2.62 | 17.2 | 9.8% (143) | 0.19 | 1.93 | 15.0 | 11.6% (129) | 0.22 | 1.93 | 12.8 |
| Total | 18.6% (640) | 0.45 | 2.44 | 17.0 | 19.1% (701) | 0.39 | 2.25 | 18.6 | 14.5% (691) | 0.30 | 2.05 | 12.9 |
