# Supplementary material for: The Impact of Hotspot-Targeted Interventions on Malaria Transmission in Rachuonyo South District in the Western Kenyan Highlands: A Cluster-Randomized Controlled Trial
Source: PLoS Med. 2016 Apr 12;13(4):e1001993. doi: 10.1371/journal.pmed.1001993 (PMC4829260; doi:10.1371/journal.pmed.1001993)
Supplement: S1 Text — (DOCX) [file pmed.1001993.s004.docx]

# 1. TITLE OF THE PROJECT:

Reducing the burden of malaria by targeting hotspots of transmission and improving malaria control measures in the highlands of Western Kenya: Simultaneous rollout of four malaria control interventions and evaluation by cross-sectional surveys.

**2. INVESTIGATORS AND INSTITUTIONAL AFFILIATIONS:**

Teun Bousema^1,2^ (PI)

Nabie Bayoh^3,4^

Jonathan Cox^1^

Meghna Desai^3,4^

Chris Drakeley^1^

Ulrike Fillinger^1,5^

Elizabeth Juma^6^

Simon Kariuki^3,4^

Kayla Laserson^3,4^

Elizabeth Marube^3,4^

Chrispin Owaga^3,4^

Jennifer Stevenson^1^

John M. Vulule^3^

^1^London School of Hygiene and Tropical Medicine, London WC1E 7HT, UK

^2^Radboud University Nijmegen Medical Centre, Nijmegen, the Netherlands

^3^Kenya Medical Research Institute, Centre for Global Health Research, Kisumu, Kenya

^4^Centers for Disease Control and Prevention, Division of Parasitic Diseases, Atlanta, GA 30341, USA

^5^ International Centre of Insect Physiology and Ecology, Thomas Odhiambo Campus, Mbita, Kenya

^6^Division of Malaria Control, Ministry of Public Health and Sanitation, Nairobi, Kenya

**3. ABSTRACT:**

The burden of malaria is not equally distributed in Kenya. Some villages experience a higher exposure to malaria compared to neighbouring villages and even within villages some households are disproportionally affected. In the Rachuonyo district in the Kenyan highlands, this heterogeneity in malaria exposure is striking with parasite prevalence rates at schools ranging from 0% to >70%. The persistence of malaria in small geographical areas despite high coverage with insecticide treated nets (ITNs) and indoor residual spraying (IRS) may be partially explained by outdoor biting and resting of malaria vectors.

These insights in the micro-epidemiology of malaria transmission in Rachuonyo district suggest that a revision of malaria control strategies may be needed to successfully control and eliminate malaria. In the current study, we propose to determine the value of rolling out four targeted malaria control efforts simultaneously in reducing malaria transmission. These targeted control efforts include local upscaling of currently used IRS and ITNs in hotspots of malaria transmission. In addition, larviciding will be employed to target malaria vectors, also those that are less susceptible to IRS and ITNs as a consequence of outdoor feeding and resting. Lastly, the human infectious reservoir will be reduced by treating parasite carriers and their household members in hotspots of malaria transmission.

The impact of these targeted interventions will be assessed in the context of currently ongoing malaria control activities in a 2-year cluster randomized trial. In the cluster randomized trial up to 8 interventions clusters will be randomized to receive hotspot targeted interventions and compared with an equal number of control clusters where the (untargeted) malaria control activities continue. The intervention will be evaluated based on changes in parasite prevalence measured in community surveys inside and outside hotspots of malaria transmission.

We expect that this approach will not only reduce the burden of malaria in these hotspots (direct effect), but will also reduce transmission in the area as a whole by an indirect community effect (indirect effect). Areas with higher parasite prevalence are likely to maintain transmission in the area by forming the main source of infection for mosquitoes; mosquitoes subsequently spread malaria over a larger geographical region. If a hotspot targeted approach proves promising in the current cluster randomized trial, it has the potential to maximize the impact of malaria control efforts in all areas with heterogeneous malaria transmission.

This protocol falls under the larger Malaria Transmission Consortium (MTC) umbrella of projects which aims to investigate the effectiveness of programmatic activities against malaria and to develop standardized methods of estimating malaria transmission in different epidemiological settings. As such, this protocol complements those studies already on-going evaluating IRS vs. ITN centred strategies (e.g. SSC 1517, 1802, 1399, 1589, 2007) and together they will provide critical information to the DOMC on the most effective malaria control strategies in highland areas of Western Kenya.

**4. LIST & DEFINITIONS OF ABBREVIATIONS, ACRONYMS**

| ACT | Artemisinin Combination Therapy |
| --- | --- |
| AL | Artemether-Lumefantrine |
| CDC | Centers for Disease Control and Prevention |
| DSS | Demographic Surveillance System |
| DOMC | Division of Malaria Control |
| EDTA | Ethylenediaminetetraacetic Acid |
| ELISA | Enzyme Linked Immunosorbant Assay |
| FSAT | Focal Screening And Treatment |
| GFATM | Global Fund against AIDS Tuberculosis and Malaria |
| HRP-2 | Histidine Rich Protein-2 |
| IPTp | Intermittent Preventive Treatment in Pregnancy |
| IRS | Indoor Residual Spraying |
| ITN | Insecticide Treated Nets |
| KEMRI | Kenya Medical Research Institute |
| LDH | Lactate Dehydrogenase |
| LLIN | Long- Lasting Insecticide Treated Nets |
| LMP | Last Menstrual Period |
| LSHTM | London School of Hygiene and Tropical Medicine |
| RUNMC | Radboud University Nijmegen Medical Centre, the Netherlands |
| MOH | Ministry Of Health |
| MTC | Malaria Transmission Consortium |
| PCR | Polymerase Chain Reaction |
| PCPB | Pest Control Product Board |
| PDA | Personalised Digital Assistant |
| PMI | Presidents Malaria Initiative |
| PSI | Population Services International |
| RDT | Rapid Diagnostic Test |
| RUNMC | Radboud University Medical Centre |
| RR | Relative Risk |
| SCR | Sero Conversion Rate |
| SP | Sulphadoxine Pyrimethamine |
| WHO | World Health Organisation |

**5. Introduction/BACKGROUND:**

The risk of malaria is not uniformly distributed between districts, between villages and even between households from the same village. Some households are disproportionally exposed to malaria and malaria incidence in these households may be more than 5-fold higher than the village average [[1-3](#_ENREF_1)]. This heterogeneity in the burden of malaria at micro-epidemiological level is largely explained by variation in exposure to infected mosquito bites [[3](#_ENREF_3)] and complicates malaria control by resulting in large variations in the impact of interventions [[4](#_ENREF_4)]. While average levels of malaria transmission may decline in an area, strongholds of intense malaria transmission may persist. This has been observed during intensive malaria control efforts on Bioko island [[4](#_ENREF_4)] and persisting malaria hotspots on Zanzibar [[5](#_ENREF_5)] and Mali [[6](#_ENREF_6)] despite overall trends of reducing transmission intensity.

In the highlands of Western Kenya, malaria transmission is also highly variable despite universal high coverage rates with insecticide treated nets (ITNs) and indoor residual spraying (IRS). In Rachuonyo district, community surveys that were conducted in 2010 indicated parasite prevalences averaging 14.8% but ranging from 0% to 51.5%. School surveys that were conducted in the same year at 46 schools in the district revealed an even more marked variation in children aged 8-14 years where parasite prevalence was on average 25.8% but ranged from 0 to 71.4%. These findings indicate that the current control efforts are insufficient to control or eliminate malaria in parts of the district. One of the reasons that may underlie this apparent failure to interrupt local malaria transmission may be the presence of outdoor biting and resting mosquitoes. Work conducted by the Malaria Transmission Consortium in Rachuonyo has recently indicated that a sizeable proportion of mosquitoes may feed outdoors in the Kenyan highlands [Stevenson *et al.*, in preparation]. Outdoor biting vectors may remain largely unaffected by control tools that target indoor biting and/or resting mosquitoes like IRS and ITNs. Additional malaria control efforts may therefore be needed in areas where transmission persists: control efforts that reduce malaria transmission regardless of the biting or resting behaviour of the mosquito vector. These targeted interventions are likely to have an impact on malaria transmission in the entire district since small areas with higher malaria transmission intensity form important contributors to overall malaria transmission. Households that are exposed to higher numbers of infected mosquito bites consequently have a higher parasite load in humans. This clustering results in an amplification of malaria transmission: households that are highly exposed to infected mosquitoes also form an important source of malaria infection for uninfected mosquitoes that can spread malaria in the rest of the community [[7-9](#_ENREF_7)]. Hotspots of malaria transmission can thereby increase the efficiency of malaria transmission by a factor 2 to 4 [7-8].

In a recent survey, we have collected information to identify micro-epidemiological patterns in malaria transmission in the Rachuonyo District in the Kenyan Highlands. In this survey, we collected filter paper blood samples from ~3,200 households in an area of 100km^2^. Hotspots of malaria transmission are identified by determining spatial patterns in the prevalence of *P. falciparum* parasites and serological markers of malaria exposure [2-3, 10]. Compared to parasite prevalence rates, serological markers of malaria exposure and haemoglobin levels have the advantage of providing information on malaria exposure that is less susceptible to seasonal variations in the force of infection [3, 11-12]. In an area in Tanzania, serological markers of malaria exposure were recently found to have a sensitivity of 93% and a specificity of 85% in identifying hotspots of malaria transmission [[3](#_ENREF_3)].

In the current study we propose to target hotspots of malaria transmission in Rachuonyo with two purposes: i) reducing the burden of malaria in areas where current control measures have proven inefficient in controlling malaria; ii) reducing the overall burden of malaria by interrupting transmission in these areas that form catalysts for overall malaria transmission. Coverage with currently available tools will be upscaled in hotspots of malaria transmission. In addition to a targeted upscaling of IRS coverage and ITN coverage, done by the study team in close communication with the DOMC, we will target water bodies within hotspots that are likely to persist for more than a week and that are found to have *Anopheles* larvae with larvicidal agents. As well as vector control measures, we will aim to reduce the human infectious reservoir in hotspots by Focal Screening and Treatment (FSAT) campaigns where symptomatic and asymptomatic individuals in hotspots of malaria transmission are screened for the presence of malaria parasites and treated with artemisinin combination therapy. This intervention follows the Kenyan National Malaria Strategy for 2009-2017 that mentions as part of strengthening the disease surveillance capacity a similar approach of targeted screening and treatment of asymptomatic malaria infections, “*… strengthening capacity to test every fever case, following up positive cases with home visits, screening household members of the index case and treating those found positive for malaria.”*[[13](#_ENREF_13)] and objectives to treat asymptomatic infections in school children as part of the malaria-free school initiative [[13](#_ENREF_13)].

The impact of the hotspot targeted approach will be evaluated by cross sectional community surveys in which we determine the prevalence and density of malaria infections and malaria-specific immune responses.

**6. Justification OF THE INTERVENTION:**

Seasonal indoor residual spraying with insecticides and campaigns to increase bednet coverage in the highlands of Western Kenya have reduced the burden of malaria in Rachuonyo district. However, the impact of the interventions is variable across the district and several areas continue to have high parasite prevalence rates among the general population and particularly among school children. These observations indicate that the current control efforts are insufficient to control or eliminate malaria in the area and additional efforts are needed.

For a maximum impact, these additional interventions should specifically aim to reduce malaria where it is most prevalent, target both indoor and outdoor biting and resting mosquitoes and also reduce the human infectious reservoir [[14](#_ENREF_14)]. When targeted to hotspots of malaria transmission, these interventions are expected to be highly efficacious in reducing malaria transmission locally and throughout the district.

**7. MAIN OBJECTIVE:**

To evaluate the impact of hotspot targeted interventions with LLINs, IRS, larviciding and FSAT on malaria indices in the community.

# 8. Specific objectives AND HYPOTHESES TO BE TESTED:

1. To evaluate the impact of hotspot targeted interventions with LLINs, IRS, larviciding and FSAT on malaria parasite prevalence inside hotspots of malaria transmission [Ho = hotspot targeted interventions will not reduce malaria parasite prevalence inside hotspots]
2. To evaluate the impact of hotspot targeted interventions with LLINs, IRS, larviciding and FSAT on malaria parasite prevalence in the area surrounding the hotspots of malaria transmission [Ho = hotspot targeted interventions will not reduce malaria parasite prevalence in the areas surrounding malaria hotspots]
3. To evaluate the impact of hotspot targeted interventions with LLINs, IRS, larviciding and FSAT on serological markers of malaria exposure inside hotspots of malaria transmission [Ho = hotspot targeted interventions will not reduce serological markers of malaria exposure inside hotspots]
4. To evaluate the impact of hotspot targeted interventions with LLINs, IRS, larviciding and FSAT on serological markers of malaria exposure in the area surrounding the hotspots of malaria transmission [Ho = hotspot targeted interventions will not reduce serological markers of malaria exposure in the areas surrounding malaria hotspots]

**9. DESIGN AND METHODOLOGY:**

## a. General study design

This study is a cluster-randomized trial where we compare the impact of hotspot targeted interventions on malaria transmission. Up to 16 clusters will be defined based on currently ongoing activities with half receiving the interventions. In each of the intervention clusters, all 4 hotspot-targeted interventions will be superimposed on ongoing control measures: hotspots will be targeted with a combination IRS, LLINs, larviciding and FSAT. The other clusters will serve as controls; these areas will not be targeted.

The interventions will be evaluated in the context of ongoing malaria control efforts. In the entire study area current ongoing malaria control interventions will continue without interruption.

## b. Study site and population.

Ethnicity in Rachuonyo is predominantly of the Luo ethnic group. Local residents depend upon farming and cattle and goat herding for subsistence. Compounds are distributed broadly across a rolling landscape intersected with small streams and rivers. The main malaria vectors in the area are *Anopheles gambiae*, *An. arabiensis*, and *An. funestus*. Malaria transmission is seasonal, with two seasonal peaks in malaria cases reflecting the bimodal rainfall pattern, with the heaviest rainfall typically occurring between April and June, with a smaller peak between October and November each year. Most malaria is caused by *Plasmodium falciparum*. Community surveys that were conducted in 2010 indicated parasite prevalences averaging 14.8% but ranging from 0% to 51.5%. School surveys that were conducted in the same year at indicated an average parasite prevalence of 25.8% (range 0 to 71.4%).

Detailed cross-sectional surveys have been conducted in an area of 100km^2^ (5x20km) in Rachuonyo district to determine small-scale variations in malaria transmission intensity. (see figure 1). This area was sub-divided into 400 500m^2^ areas referred to as cells.


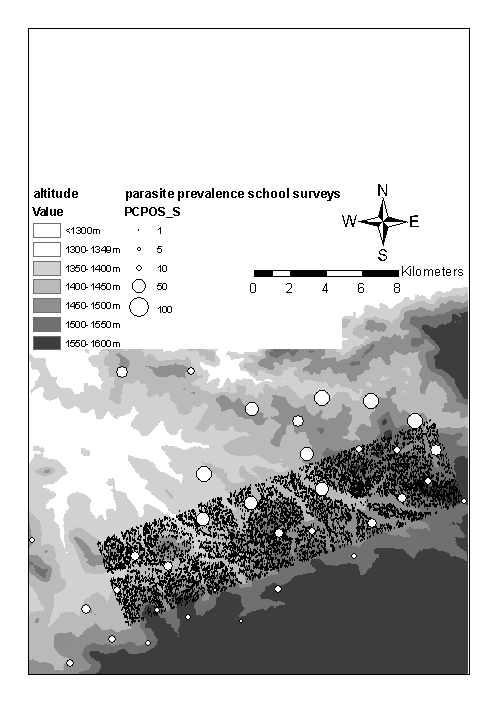


*Figure 1. The Rectangle indicates the area (5*20km) in Rachuonyo district where the activities will take place. Each dot is an individual household. White dots indicate the parasite prevalence in school surveys in the area in the year 2010; colour intensity indicates variation in the altitude. The proposed study area lies at an altitude of 1400-1550 meters.*

All structures in this 5 x 20km area were geo-located by remote sensing and 3,200 compounds were randomly selected for detailed characterization of malaria exposure. After informed consent, household characteristics were recorded for all houses that belonged to the selected compound. Temperatures were taken from each permanent resident of the selected compounds and individuals were asked to provide a single finger-prick blood sample for detection of malaria parasites by PCR [15], malaria-specific antibody responses by ELISA [[12](#_ENREF_12)] and haemoglobin concentration by HemoCue photometer [Angelholm, Sweden]. Febrile individuals were screened in the field using rapid-diagnostic tests [Paracheck, Orchid Biomedical Systems, India] and treated with first line artemisinin-combination therapy.

## c. Definition of clusters for interventions

## The data collected during the intensive cross-sectional surveys (SSC 1802) will be used to detect hotspots of malaria transmission in the study area based on malaria parasite prevalence and antibody responses. Hotspots will be defined as areas that are smaller than 1km^2^ with a level of transmission intensity that exceeds that in the surrounding area (in all directions). This will be indicated by a statistically significant higher parasite prevalence in combination with a statistically significant higher SCR and/or age-adjusted density of antibodies malaria antigens.

Malaria parasite prevalence will be determined by PCR-based detection of parasites based on the *P. falciparum* mitochondrial cytochrome b gene [15].

Malaria specific antibody responses will be quantified as the age-dependent seroconversion rate (SCR) for antibodies against malaria: antigens apical membrane antigen-1 (AMA-1), merozoite surface protein-1 (MSP-1_19_) and glutamate-rich protein (GLURP) [[12](#_ENREF_12)]. Antibody responses will be measured by standard ELISA methodology [12, 16] and the SCR will be determined by fitting a simple model to the age-dependent prevalence of antibody responses, as described in detail in publicly available protocols [16]. In addition, age-adjusted antibody densities will be calculated [17]. The SCR will be estimated for geographical subsets of 50 observations.

Hotspots will be of variable size and no quantitative estimate of the elevated transmission intensity inside hotspots can be given a-priori since this depends on the transmission intensity of the surrounding area [2-3,16]. Clusters will include hotspots and a surrounding ‘evaluation zone’ of 500m from the edge of the hotspot (see figure 2); this forms the unit of randomization. The grid structure that divided the study area into 250x250m cells, created as part of the baseline cross-sectional study will be used as easy references for the areas defined as hotspots. The size of a hotspot or intervention area will not be defined by the maximum mosquito dispersal range. We are not aiming to identify independent niches of malaria transmission. Minimum distance between hotspots will be 1km. Mosquito dispersal outside hotspots of malaria transmission is essential for the community effect and forms the cornerstone of our evaluation.


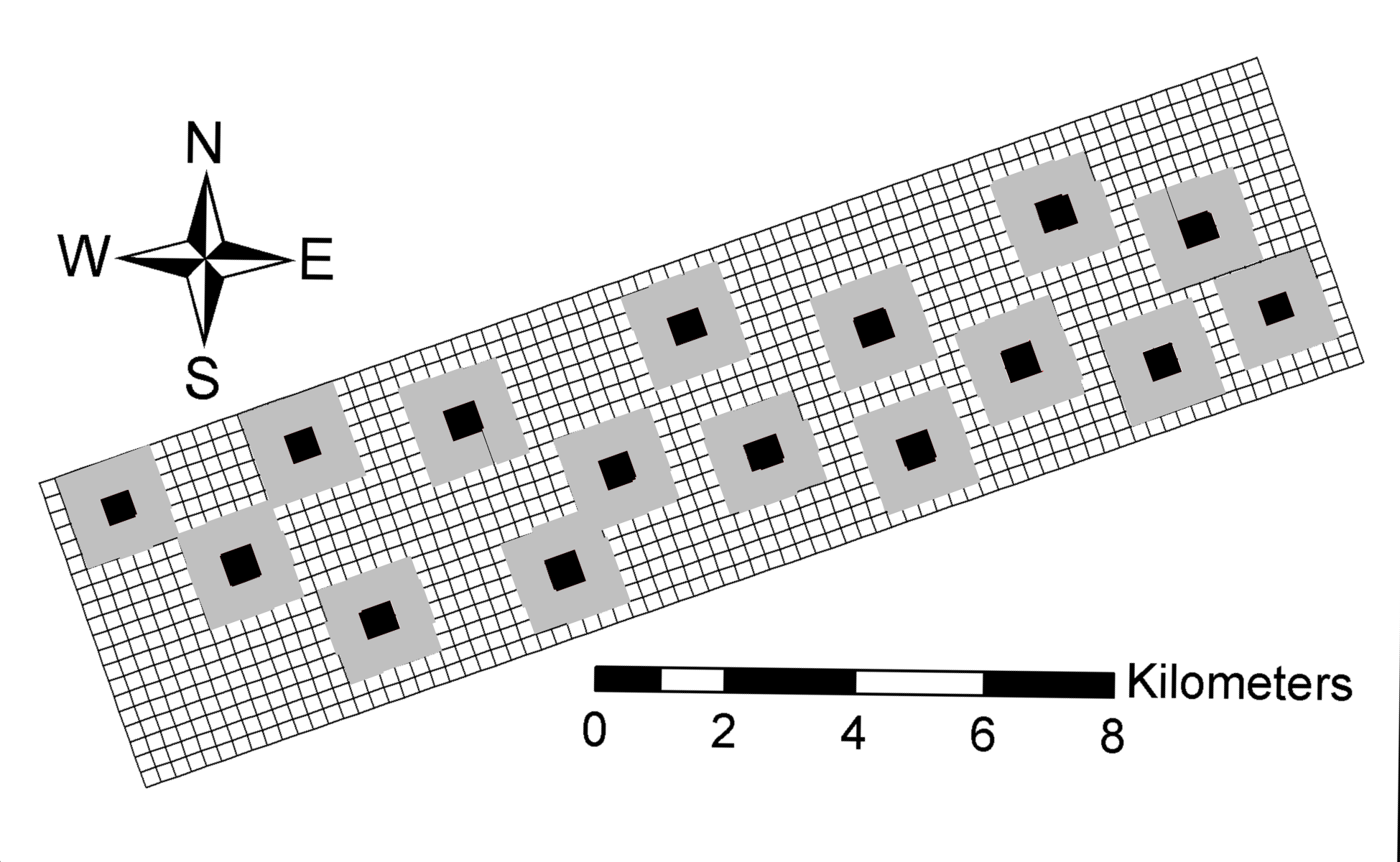


## *Figure 2. Example of possible layout of hotspots (black blocks) and the surrounding areas (grey blocks) where hotspot targeted interventions will be evaluated). The depicted grid cells are 250x250m.*

## d. Intervention

Up to sixteen clusters will be defined as in the example shown in figure 2. Each cluster consists of a hotspot of malaria transmission and the area surrounding the hotspot 500m in each direction. In the entire study area current malaria control interventions will continue without interruption. In the intervention clusters (i.e. half of the identified hotspots), interventions will be superimposed on ongoing control measures: these intervention hotspots will be targeted with IRS, LLINs, larviciding and FSAT. All the intervention measures will be discussed in more detail in section f. ‘Procedures- Intervention Phase’ however, a brief overview is provided below.

*Indoor Residual Spraying (IRS)*

IRS will be carried out twice per year in all households in the intervention hotspots in the period preceding the long rainy season (March 2012) and six months later (September 2012). Meetings will be held with DOMC to discuss the optimal insecticide for use in the context of ongoing IRS activities in the district.

*Long Lasting Insecticide Treated Bednets (LLIN)*

All compounds in intervention hotspots will be visited in March 2012 and each house will receive a Long Lasting Insecticide Treated Nets for each sleeping space. The DOMC will be consulted about which type of LLINs should be distributed and how best to approach this, but activities will be undertaken by study personnel A follow-up visit is planned one month and 6 months after LLIN distribution to provide additional nets where needed and to monitor accurate use.

*Larviciding*

All mosquito larval habitats in all hotspots and evaluation zones will be mapped using handheld GPS systems in January. All habitats found to persist for more than a week within intervention areas will be treated with a larvicide that has been approved by the Pest Control Product Board and by DOMC, *Bacillus thuringiensis var. israelensis* (Bti) and/or *Bacillus sphaericus* (Bs). Larviciding will begin in March 2012 and continue weekly throughout the rainy season (until July 2012).

*Focal screen and treatment (FSAT)*

All compounds in hotspots will be visited in March 2012, and children between 6 months and 20 years of age will be tested for malaria parasites using a rapid diagnostic test based on the detection of malaria antigens (Premier Medical First Response Combo^®^,) using one of the best tests in a recent evaluation of the World Health Organization [18]. This test is based on the detection of *P. falciparum* specific histidine rich protein-2 (HRP-2) and Lactate dehydrogenase (LDH) and thereby circumvents problems of false-positivity common to HRP-2 based tests. If a single individual in this age group is found positive by RDT every individual within this compound will be treated with AL except for pregnant females. For these individuals transport will be provided to a health facility in the area and costs covered for examinations and government recommended anti-malarial treatments. Children under the age of 6 months will not be treated. A second round of FSAT is planned for September 2012.

Consent will be obtained separately for each intervention at the DOMC, administrative and community level (all interventions), the compound level (IRS and LLINs), or the individual level (FSAT). In the case of permanent breeding sites that are privately owned (e.g. fish ponds) verbal consent will be sought from community and owners before larviciding takes place. Other studies have shown no negative impact of larviciding using *Bacillus thuringiensis var. israelensis* on fish population and acceptance of larviciding by fish pond owners and the community (Kahindi *pers comm.*).

The other clusters will serve as control areas; hotspots in these areas will not be targeted.

**Routine malaria control efforts in eight control clusters**

Any currently planned distributions of LLINs and yearly rounds of IRS will continue in the rest of the area. No interventions will be withheld from the population but no hotspot-targeted interventions will take place in the control clusters.

## d. Outcomes

*Primary outcome:*

1. The prevalence of *Plasmodium falciparum* parasitaemia as assessed by community surveys and measured by microscopy, RDT and PCR in hotspots of malaria transmission and in areas surrounding these hotspots.

*Secondary outcomes:*

1. The prevalence and density of serological markers of malaria exposure, antibodies against malaria-specific antigens, in hotspots of malaria transmission and in areas surrounding these hotspots.

## e. Sampling strategy and sample size calculation

The hotspot-targeted intervention will be evaluated inside and outside hotspots of malaria transmission. The latter is the main outcome measure of interest and forms the basis of sample size calculations. All available malaria simulation models indicate that malaria transmission in the area surrounding intervention hotspots would be considerably decreased because malaria transmission is effectively reduced/interrupted in those compounds that seed transmission to a larger geographical area [7-8, 19]. Because there are no published studies on the impact of hotspot targeted interventions, we determined the predicted impact of targeted interventions in our study area by one of the leading individual-based simulation model [19] using human, entomological and parasitological characteristics collected at our sites in Kenya. A previous study on the community benefits of insecticide treated nets in Asembo Bay, western Kenya, indicated that an indirect beneficial effect on malaria transmission may be limited to 500m from the intervention area [20]. We used this finding to define our evaluation zone surrounding the hotspots.

| 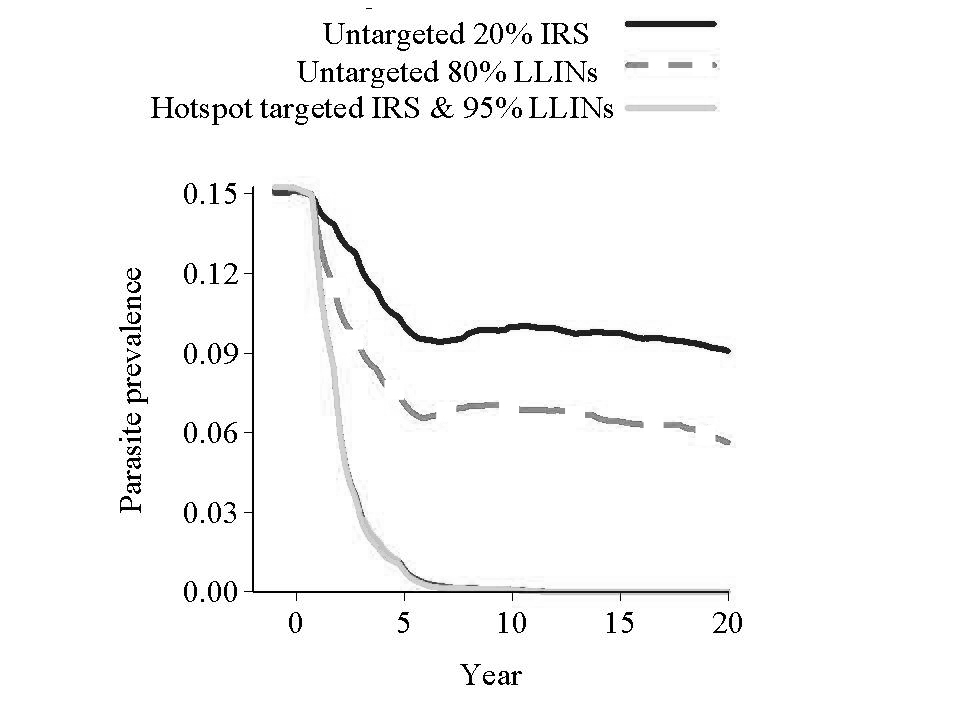 |
| --- |

***Figure 3. The predicted impact of untargeted and targeted interventions predicted by an individual-based simulation model.*** *Plotted are smoothed lines, the actual model estimates allow for seasonality.*

We modelled three scenarios in situations with a pre-intervention parasite prevalence in the human population of 5-15%: i) indoor residual spraying (IRS) in an untargeted approach, reaching 20% of the total population (black top line); ii) effective coverage with long lasting insecticide treated nets (LLINs) in an untargeted approach, reaching 80% of the total population (middle dashed grey line); iii) targeted IRS and 95% effective coverage with LLINs in the hotspots (bottom light grey line). The impact of larviciding is currently insufficiently parameterized to be included in the model [19].

We assumed a conservative estimate of the efficacy in detecting hotspots (75%, compared to 93% in Tanzania [[3](#_ENREF_3)]). Our simulations showed that targeted interventions can interrupt transmission completely, both inside and outside hotspots of malaria transmission, reducing overall parasite prevalence from 15% to <0.1%, in a manner that appears sustainable in the following years (see figure 3).

Our primary outcome of interest is the impact of hotspot-targeted interventions on parasite prevalence in the area surrounding the hotspot (the evaluation zone). If we evaluate our intervention in 300 randomly selected individuals living outside the hotspot per cluster and assume a conservative estimate of k (0.5 coefficient of variation of true proportions between clusters within each group), we need 8 clusters per arm to detect a reduction from 10% parasite prevalence at baseline to ≤ 5% after the intervention. The actual number of people sampled will depend on the size of the hotspot and surrounding evaluation zone, with a target of sampling 20% of the population in the evaluation zone (≤500m from edge hotspot) or a minimum of 100 and maximum of 500 people. The unit of randomization will be at the compound level therefore 100 people would equate to approximately 35 compounds.

We aim to evaluate the interventions in all age groups above 6 months. If the PCR results of the intensive survey indicates that parasite prevalence is below 10% in certain age groups, these age groups will be considered less suitable for evaluation purposes; they will receive the standard of care provided by the study, including screening and treatment of all febrile cases, but will not be routinely sampled.

To understand possible effect differences between clusters, we also want to determine whether our intervention succeeded in reducing malaria transmission inside hotspots. The estimated parasite prevalence in individuals between 6 months and 20 years of age who are living in hotspots is >20% (Stevenson *et al.*, unpublished data). With a sample size of 100 individuals per cluster (i.e. per hotspot in 16 clusters), we would be able to detect a reduction to ≤ 5% after the intervention (k=0.5; Z_α/2_=1.96; Z_β_=0.84). Again the actual number sampled will depend on the size of the hotspot, with a target of sampling 20% or a minimum of 50 individuals (approximately 9 compounds).

## f. Procedures: intervention phase

*Consent procedure intervention phase:*

Prior to the implementation of the interventions, meetings will be organised with the district commissioner, officers and chiefs with the district health team to seek approval for the study. Sensitization of communities will be done by chiefs and assistant chiefs and village elders from the study area. Community meetings (barazas) will be held in the selected areas and study staff will explain the purpose of the study, the procedures to be followed and the risks and benefits of participation. Information sheets shall be provided in English, DhoLuo, and Kiswahili to all district representatives, chiefs and households (Appendix A).

All consent forms will be available in English, DhoLuo, or Kiswahili. Translations will be checked through back-translation (Appendix B). Before the targeted distribution of LLINs and IRS, informed consent will be obtained at the household level from the head of the household; this will be recorded by signature or thumb-print which is then overseen by the signature of an independent witness. Before FSAT, informed consent will be obtained at the individual level; this will be recorded by signature or thumb-print accompanied by the signature of an independent witness of selected individuals and/or their parents/guardians. Assent forms will be signed by children between the ages of 13 and 17 years and by their parents/guardians. Each assent form will be accompanied by consent form signed by the parent/guardian.

Larviciding will be done after consulting with and receiving approval from the DOMC and PCPB and after community meetings. Verbal consent will be sought from any privately owned permanent breeding sites in the intervention areas (e.g. fish ponds) after distributing information sheets. Since most mosquito breeding sites are not necessarily restricted to certain households and larviciding should be seen as a community intervention [21-22] [21], consent at household level is not practical and approval from the community, DOMC and PCPB will be sought instead.

*Timing of the intervention:*

March-May 2012

The first round of interventions in all targeted hotspots will be completed before the start of the transmission season in May 2012. IRS, LLINs, larviciding and FSAT have the largest predicted impact if started before the start of the transmission season [19]. Follow-ups for LLIN coverage and usage will occur 1 and 6 months after initial distribution (see table in section 11). Clusters will be randomly allocated to the intervention or control arm. Based on a trial in Kwale District, Coast, a public randomization approach will be used to encourage participation among the community and to promote transparency of the selection procedure (Brooker et al., 2010). Meetings will be called with district officials, chiefs, community stakeholders and elders to explain the purpose and visually demonstrate the randomization process.

Depending on the outcomes of the first year of the intervention, in consultation with the DOMC, we aim to roll-out the most efficacious intervention/s to all hotspots in the study area.

*Procedure intervention:*

Compounds in intervention clusters that have been selected for the intensive malaria control will be visited in January-February for mapping, to update census lists, (appendix C) and explain the study procedures.

*Larviciding*

Persistent breeding sites will be mapped using handheld GPS systems in all clusters. In the period preceding the long rainy season (March-April 2012), all permanent aquatic habitats inside hotspots and those located on the fringe of hotspots but are a likely source of anophelines in hotspots based on expert opinion, will be treated at weekly intervals with approved larvicides (*Bacillus thuringiensis var. israelensis* (Bti) and/or *Bacillus sphaericus* (Bs). Within a hotspot all mapped water bodies will be treated by trained personnel and any other non-mapped water bodies will be dipped to search for *Anopheles* larvae and also treated if larvae are found. One week later the area will be revisited and all water bodies dipped again for anopheline larvae. Any positive sites will be treated. This will continue throughout the transmission season (May – July 2012).

Mapping of all persistent breeding sites will be used to initially locate sites within hotspots for larviciding and secondly to calculate distance to breeding sites from study compounds both in intervention and control clusters. This is necessary to take into account variability in distribution of breeding sites whether treated or not.

*Indoor residual spraying*

The insecticide that will be used for IRS will be decided on following consultations with DOMC and will be one of the following: lambdacyhalothrin, alphacypermethrin or deltamethrin. Activities and actual spraying will be undertaken by study personnel; IRS will be timed before the peak transmission season in March, at the time-point that is deemed most efficacious by comprehensive malaria transmission models [19] and again after 6 months.

Heads of compounds will be visited by the study team and consent sought. Compounds where the head, or someone with the authority to provide consent, is absent will be visited for a maximum of three times to maximize IRS-coverage in the hotspots. The study team will remove all movable items from the structures that will be sprayed. Any large items that cannot be removed will be moved into the centre of the room and covered with plastic sheets. Sprayers will use protective clothing, masks and gloves and will dispense the insecticide using a back pack sprayer. The walls and roof of all structures in the compound (residential and non-residential) will be sprayed for at least 15 minutes according to guidelines for the insecticide used. The structures will be left locked for 2 hours after which the doors and windows will be opened and allowed to air out for 30 minutes. After this the head of the house may enter to sweep clean. During the government-led IRS program of 2011 lambda-cyhalothrin was used and was reported to occasionally result in itching. Approximately 1 week after the spray households will be revisited to interview members on any reported side effects (see appendix F& H).

Spraying will be repeated at 6-monthly intervals (visits 1 and 3).

*Long-lasting insecticide treated nets distribution*

At the point of the census study staff will document the number of sleeping spaces within each house. At the time of the intervention (March 2012), the study team will distribute one LLIN for each sleeping space within the house, hang the net and explain the proper use and maintenance of the net. All study nets will be labelled for later identification. To avoid potential irritation to the insecticide used in the LLIN, nets will be hung outside for at least 24 hours before being distributed to communities to allow the strength of the insecticide to wane.

During a second visit, one month after initial LLIN distribution the correct usage of LLINs will be evaluated: the correct hanging of LLINs will be confirmed and household members will be asked to explain how they use the net. If needed, instructions about correct use will be repeated and additional nets can be provided if nets are missing or found to be torn. A third visit will occur to re-assess the quality and use of the nets 6 months after the first visit, in conjunction with the second round of IRS. At this point new ownership and quality will be re-assessed and new nets will be distributed as needed. All information on net use and quality will be recorded (appendix D).

*Focal Screening and Treatment (FSAT)*

Screening of a sentinel population within households

The FSAT utilizes individuals aged 6 month to 20 years as a sentinel group to indicate malaria exposure in the household because this age-group experiences the highest parasite burden [23-24]. For this age group all individuals will be tested for parasites using RDTs regardless of presence of symptoms or not. However clinical care will not be restricted to just this age group. All age groups may experience clinical malaria in our setting (Stevenson *et al.*, in preparation) and therefore those individuals over 20 years who are found to have fever will also be tested by RDT and receive treatment as oart of the FSAT intervention.

The FSAT campaign will take place in intervention hotspots in March 2012; a second round of FSAT is planned to take place in September 2012. Both campaigns will follow identical procedures.

Screening the sentinel group

Blood samples will be collected from all individuals aged between 6 months and 20 years. Blood will be collected by fingerprick and used to test for malaria by rapid diagnostic test (Premier Medical First Response Combo^®^,HRP-2 & LDH based), for determination of haemoglobin levels using a Copack Colour Scale ® (Oststeinbek, Germany), to prepare a microscopy slide and to collect blood into an EDTA microtainer tube and onto a filter paper. RDTs will be used for clinical decision-making; slides will read after the campaign to determine the relative sensitivity of RDTs and PCR in the study setting. The Copack Haemoglobin Colour Scale system has been available since 2001 and is a reliable and validated method to assess haemoglobin levels. The test consists of a drop of blood being placed on the test strip, and after 30 seconds is compared to the hue on the provided colour scale. The gradient of the colour scale indicates whether and how severely anaemic a person is with the scale ranging from <4 to 14 g/dl of haemoglobin. The collected blood samples will be analysed for parasite prevalence by microscopy PCR and the detection of malaria-specific antibody responses by ELISA (see section g: laboratory analyses).

Screening the non-sentinel group

In addition to testing the sentinel age-group, all other individuals aged above 20 years will have their temperature by ear thermometer and blood samples taken by fingerprick. Those that are febrile (as defined by the range of the specific thermometer used) will be tested for malaria using RDTs otherwise blood will be collected as described above for the same purposes.

Using the sentinel population to detect microscopic and submicroscopic parasite carriers

If no parasites are detected in the sentinel age group, it is unlikely that any parasite carriers are present in the household. If, on the other hand, one or more participant between 6 months and 20 years are parasite positive, it is likely that household members are also infected. Household members of malaria-infected individuals are up to 10-fold more likely to be infected with malaria themselves [2, 25][Mosha *et al.*, unpublished findings]. This household clustering of infections often occurs below the threshold density that allows infections to be detected by microscopy or RDTs. In other words, in households where somebody carries parasites as confirmed by RDT, there is highly significant clustering of low density infections that are negative by RDT and slide but positive by PCR [14, 25]. Up to 50% of all infections may be missed by RDTs or microscopy in settings like the Kenyan highlands because the density of infections is often very low [14](Stevenson, in preparation). These asymptomatic low-density infections are clinically and epidemiologically relevant and can result in chronic anaemia and continued malaria transmission [35].

Treatment once parasites are detected in a household

We will give all people who reside in the same house as anyone (in the sentinel or non-sentinel group) found to be RDT positive a complete curative dose of antimalarials to clear parasite densities, except those under 6 months of age and pregnant women (see below), This focal treatment of parasite carriers in hotspots will maximize the impact of FSAT [26] since it includes symptomatic and asymptomatic, microscopic and submicroscopic parasite and gametocyte carriers. A proportion of those people who stay in the same house as parasite carriers will be themselves be free of detectable parasites at the moment of FSAT. Since there is currently no molecular assay that can detect these sub-microscopic infections in field settings, our approach is the only operationally feasible way to include microscopic and sub-microscopic parasite and gametocyte carriers without exposing individuals who are exposed to low malaria risk (i.e. those living outside hotspots of malaria transmission) unnecessarily to antimalarial drugs. In line with this, our approach should not be interpreted as mass drug administration since our treatment is restricted to small (<1km^2^) areas with proven high exposure to malaria.

Treatment will be carried out using artemether-lumefantrine (Coartem, AL) according to national guidelines. AL is an antimalarial drug with an excellent safety profile; limited data on safety are available for children under 6 months and pregnant women, both groups will therefore be excluded from AL treatment (see below) but receive clinical care when necessary. As detailed below, all those who are given Coartem will be actively followed-up to monitor any side effects. This monitoring will happen on all days of treatment and one week after the start of treatment. If given to parasite-free individuals, AL will provide an effective prophylactic effect for 3-4 weeks, which has been shown to be highly beneficial for malaria control purposes in high endemic regions [36]. In parasite carriers, AL effectively clears asexual parasites and gametocytes, the parasite stage responsible for transmission [35]; the timing of FSAT is chosen to ensure individuals are parasite and gametocyte-free before the start of the peak transmission season. For those receiving AL, the first dose will be supervised and then instructions will be provided for taking of the second dose which will be issued. Teams will return to compounds the next day to administer and supervise the third dose and distribute the fourth. They will return again on the third treatment day to again administer and supervise the fifth dose and distribute the sixth. At the same time any side effects will be noted. (see appendix G & H).

All participants will be told their haemoglobin level immediately, and any person found to be anaemic will be provided haematinics according to national guidelines.

Treatment of risk groups: children <6 months and pregnant women

Children below 6 months of age will be excluded from the study; they will be screened for fever and referred to a health facility where needed but will not be included in FSAT.

Pregnant women will not receive AL. Prior to consent and treatment, it will be explained why AL cannot be given to pregnant women and therefore the necessity to assess whether women of childbearing age may be pregnant if anyone in their household is found to be positive for malaria. Women may opt not to be tested for malaria. In each survey team there will be a female field worker trained to carry out interviews and test for pregnancy. She will approach women of childbearing age (13-45 years) in households where there is ≥1 parasite positive participant. These women will be asked whether they could be pregnant, and the date of their last menstrual period. Women whose last menstrual period was not within the prior 4 weeks, and/or who could be pregnant will be asked whether they would be willing to perform a pregnancy test on their urine sample. A recent survey of houses in the study area showed all to have pit latrines where a urine sample can be collected and a pregnancy test can then be conducted by the field worker away from the house. Women will be provided results of their pregnancy test in a private area in a confidential manner. This was shown to work in a sister study (SSC 1517). Confidentiality will be ensured at all times. Females whom have a negative pregnancy test can be given AL.

Women with a positive pregnancy test or those who would rather not have a pregnancy test performed will be offered a test for malaria parasites by RDT. If a woman who is pregnant or may be pregnant is parasite positive by RDT, she will be offered to be taken to a nearby health facility for examination and appropriate treatment before being returned home. If a women who is pregnant or may be pregnant is parasite negative by RDT, she will be asked about her last dose of intermittent preventive treatment in pregnancy (IPTp) with sulphadoxine-pyrimethamine (SP) and her antenatal records will be consulted. If the IPTp dose of SP was given <4 weeks ago, no additional treatment will be given. If the dose is >4 weeks ago or a woman has never received IPTp and the woman is eligible for a dose of SP according to national guidelines, she will be offered to be taken to a nearby health facility for examination and appropriate treatment before being returned home. All costs incurred by the woman will be covered by the project.

As part of the supervisory team a qualified Clinical Officer will be available to advise on all treatment regimes and any side effects from drugs taken.

## g. Procedures: evaluation phase

**Community cross-sectional surveys**

*Consent procedure cross-sectional surveys:*

As with the intervention phase, meetings will be organised with the district commissioner, officers and chiefs with the district health team to seek approval for the evaluation phase. Sensitization of communities will be done by chiefs and assistant chiefs and village elders from the study area. Community meetings (barazas) will be held in the selected areas and study staff will explain the purpose of the surveys, the procedures to be followed and the risks and benefits of participation. Information sheets shall be provided in English, DhoLuo, or Kiswahili to all district representatives, chiefs and households (Appendix A).

Households will be informed at the barazas of the timing of the survey and that their compounds may be visited.

On the day of the cross-sectional survey informed consent will be obtained at the individual level. All consent forms will be available in English, DhoLuo, or Kiswahili. Translations will be checked through back-translation (Appendix B). Consent will be obtained by signature or thumb-print accompanied by the signature of an independent witness of selected individuals and/or their parents/guardians. Assent forms will be signed by children between the ages of 13 and 17 years and by their parents/guardians. Each assent form will be accompanied by consent form signed by the parent/guardian.

*Timing of the cross-sectional surveys:*

Six cross-sectional surveys will be conducted (see table in section 11):

- Pre-transmission season (March 2012)
- Peak transmission season (June 2012)
- Post transmission season (September 2012)
- Pre-transmission season (March 2013)
- Peak transmission season (June 2013)
- Post transmission season (September 2013; final survey)

*Procedure cross-sectional surveys:*

Prior to the surveys, census lists for all hotspots will be updated using PDAs, recording the name, age and gender of each household occupant (see appendix C for summary of information collected). For each cross-sectional survey up to 100 individuals above the age of 6 months will be randomly selected from malaria hotspots and up to 500 participants above the age of 6 months will be randomly selected from outside malaria hotspots in each cluster. Randomization will occur at the compound level.

Each occupied house in the randomly selected compounds will be visited by a team of fieldworkers. The head of the house will then be interviewed using a personalized digital assistant (PDA) to record information. The interview will assess household construction, wealth indices and use of anti-malarial measures (recent IRS activities, bednet ownership and use, use of mosquito coils, sprays etc.). Each selected participant or their parent/guardian will be interviewed inquiring about recent fever episodes, treatment seeking behaviour, ITN use and travel history. The transcript of the questionnaire is given in Appendix E. On the PDA translations for questions will be available by pressing buttons for DhoLuo or Kiswahili. The questionnaire it will be broken down into 4 parts; the first records details of the household, the second logs bednets, the third records personal details and the last records results of tests completed.

Temperature will be measured by ear thermometer; a single fingerprick blood sample will be used to i) determine haemoglobin levels using a Copack Haemoglobin Colour Scale system (blood sample spotted onto a special filter paper), ii) prepare a microscopy slide (sample taken directly from the finger onto a slide) and iii) collect a blood sample into an EDTA microtainer tube and onto a filter paper for parasite PCR and the detection of malaria-specific antibody responses by ELISA. For all individuals with fever (≥37.5°C), the presence of malaria parasites will be determined immediately by rapid diagnostic test (RDT, directly from the finger). Anyone with fever and a positive RDT will be treated for malaria at the home according to national guidelines (see above for procedures women of childbearing age). All participants will be told their haemoglobin level immediately, and any person found to be anaemic will be provided haematinics according to national guidelines. The volume of the blood sample taken for all measurements combined will be 200-300μL.

**Laboratory analyses**

Blood samples, slides and filter papers will be delivered to KEMRI/CDC laboratories in Kisumu. Here they will be processed to separate plasma and extract parasite DNA. Serological markers of malaria exposure will be determined in the microtainer-derived plasma samples, including IgG antibodies to malaria antigens [27]. We will also assay for antibodies to the circumsporozoite malaria antigen and to proteins and peptides which are components of mosquito saliva [28]. This will allow an assessment of recent exposure to both vectors and parasites. Microtainer-derived parasite DNA samples will be assayed using PCR to identify low-density parasite infections [29]. Filter paper blood samples will be taken as stable back-up sample for serum elution [16] and DNA extraction [29]. We will also use molecular techniques to look for markers that may be associated with treatment failures or contraindications for example *pfcrt, pfmdr,* or G6PD and markers associated with increased risk for contracting e.g. Duffy and transmitting malaria. A subsample (10%) of dried blood samples will be assayed for serology and related parasite PCR at LSHTM, UK and RUNMC, The Netherlands to ensure assay quality and quality assurance but the majority of laboratory analyses will be carried out at KEMRI/CDC Kisumu.

All questionnaires on the PDA will have the participant name, person ID, and barcode number. RDTs, slides, filter papers and blood samples will be labelled using the person ID and barcode number. No names will appear any of these samples. After the questionnaires are completed, the PDAs will be returned daily to the data management staff in the field for downloading and back up while the RDTs.

**10. DATA MANAGEMENT:**

a. Data storage

All data from the questionnaires during cross-sectional surveys will be collected on PDAs and downloaded each evening into Microsoft Access. Paper records will be used as a back-up to the PDAs for recording visited households and RDT and anaemia results. All consents, assents, PDAs and computers to which data are downloaded will be locked so that only the principal investigators and data management staff will have access to the data. Data will be cleaned on an on-going basis.

b. Data analysis

To assess the impact of the intervention, methods appropriate for cluster-randomized trials will be used [30]. The primary outcome will be PCR-confirmed parasitaemia. The prevalence by PCR in each cluster will be calculated and the unadjusted risk ratio (RR), or mean difference between clusters will be calculated. An overall estimate of programme impact will be obtained, and 95% confidence intervals will be adjusted for observed between-cluster variance. Formal hypothesis testing will be undertaken using stratified unpaired t-tests. Adjustment for covariates will be undertaken replacing the cluster means and proportions with covariate-adjusted residuals from regression analysis of individual-level data.

One of the key outcomes of the study is the community-wide effect of hotspot targeted interventions. To address the question of the distance over which this community-wide effect exists, we will relate parasite prevalence and antibody prevalence to the distance from the centre of the hotspot in intervention and control clusters.

Additionally, the analysis adjusts for baseline characteristics on malaria infection, haemoglobin concentration and socio-economic status. Statistical analysis will be carried out using STATA^®^ software, version 10.0. (Stata Corporation, Texas, USA).

**11. TIME FRAME/DURATION OF THE PROJECT:**

The preparatory work for this study is set to start in January 2012. The LLIN and FSAT interventions will be rolled out at the same time as the baseline cross-sectional study (March 2012/2013). The IRS and larviciding will take place immediately afterwards. Larviciding will be repeated weekly throughout the rainy season (March-July 2012/2013). IRS and LLIN top-up distribution will be repeated 6 months later after the post-transmission season cross-sectional survey (late September 2012/2013). See timeline below.

|  | 2012 | | | | | | | | | | | | 2013 | | | | | | | | | |
| --- | --- | --- | --- | --- | --- | --- | --- | --- | --- | --- | --- | --- | --- | --- | --- | --- | --- | --- | --- | --- | --- | --- |
| Activities | J | F | M | A | M | J | J | A | S | O | N | D | J | F | M | A | M | J | J | A | S | O |
| Community Engagement |  |  |  |  |  |  |  |  |  |  |  |  |  |  |  |  |  |  |  |  |  |  |
| Mapping Study Area |  |  |  |  |  |  |  |  |  |  |  |  |  |  |  |  |  |  |  |  |  |  |
| Updating  Census |  |  |  |  |  |  |  |  |  |  |  |  |  |  |  |  |  |  |  |  |  |  |
| Intervention: FSAT & ITNs |  |  |  |  |  |  |  |  |  |  |  |  |  |  |  |  |  |  |  |  |  |  |
| Intervention: IRS |  |  |  |  |  |  |  |  |  |  |  |  |  |  |  |  |  |  |  |  |  |  |
| Intervention: Larviciding |  |  |  |  |  |  |  |  |  |  |  |  |  |  |  |  |  |  |  |  |  |  |
| Cross-sectional survey |  |  |  |  |  |  |  |  |  |  |  |  |  |  |  |  |  |  |  |  |  |  |

**12. ETHICAL CONSIDERATIONS:**

a. Consent

Participation in the study is fully voluntary and participants have the right to refuse at any time during the study. Consent will be obtained in the preferred local language, DhoLuo, Kiswahili, or English directly from all adults participating in the cross-sectional survey. For children less than18 years of age, consent will be obtained from parents or guardians. In addition, child assent will be obtained for children who are greater than 13 but less than 18 years and live with a parent or guardian. Children providing assent will be asked to sign next to their name on the assent form. As defined in the Kenya national guidelines for HIV counselling and testing, Kenyan subjects between 13 and 17 years of age who are pregnant, are married, head of a household, or a parent are considered “mature minors” and are able to consent for themselves [31]. Mature minors will be consented directly (therefore assent will not be sought). The consent and assent forms are given in Appendix B. The reading level of the consent form is 8.5. Data from the demographic surveillance system (DSS) in Nyanza Province showed that approximately 77% of mothers had primary school education [32] which is approximately equivalent to the 8th grade in the US.

During the consenting process, the study will be explained and the consent form will be read to each person. It will be emphasised that participation is voluntary. The consent form will detail the design of the study, the questionnaires, the finger prick, and the blood samples taken. If participants agree, they will be asked to sign the consent or assent form. The storage of the blood samples will then be explained to each person. Everyone will have the option to consent to have their blood samples stored at KEMRI/CDC, and/or to consent for part of the analyses to be conducted outside Kenya. Samples will be stored for 5 years to adhere to Good Clinical Practice guidelines. All persons will be provided with a copy of the blank consent form for their records after they have agreed to participate in the study.

b. Benefits to intervention participants

*Benefits to participants in intervention area*

Intervention participants will receive intensive malaria control for their household: IRS, LLINs, larviciding and in FSAT. These interventions are expected to have a direct and an indirect beneficial effect on the level of malaria the household is exposed to: IRS will reduce indoor mosquito exposure, LLINs will protect individuals from (infected) mosquito bites, larviciding will reduce indoor and outdoor mosquito exposure; FSAT will clear parasite carriage and provide 3-4 weeks of efficacious prophylaxis against malaria infections. These interventions are provided free of charge.

The wider community, i.e. those living in the vicinity of hotspots will not receive any direct benefits from the interventions, but are likely to experience indirect effects. Reductions in neighbouring areas with high malaria prevalence may result in reduced malaria transmission in the wider region.

*Benefits to participants in control area*

Participants in the control areas will not benefit from the interventions in the short term but will benefit from free screening and treatment for symptomatic malaria. However, the information that will be learned as a result of this study can benefit them in the future through more effective and targeted malaria control interventions and the possible overall reduction in malaria transmission that may ensue. There are plans for district-wide government-led interventions such as IRS to take place. All these will continue as planned and none will be withheld from the communities. Depending on the outcomes of the first year of the intervention, in consultation with the DOMC, to roll-out the most efficacious intervention/s to all hotspots in the study area.

*Community benefits*

The community benefit of the project is that we expect malaria transmission in the entire area to be reduced as a result of our hotspot targeted interventions. Free LLINs and intensive vector controlled will be offered to individuals residing in some of the strongholds of malaria transmission in Rachuonyo district; plausibly leading to a direct benefit to this part of the population that is most affected by malaria. The MOH is also expected to benefit from this study by gaining a lot of information on the status of malaria transmission in this area of Rachuonyo district as the findings will be shared with them. The Government of Kenya may also benefit by improving malaria control strategies and targeting efforts where they can be most effective.

c. Risks to intervention participants

None of the interventions that are proposed in this study are experimental. LLINs and IRS are part of the malaria control efforts by the DOMC for the district; larviciding has been done in districts bordering or close to Rachuonyo district, in Nyando, Kisii, Suba and Kakamega districts [22]. All insecticides used will have been approved by DOMC and PCPB prior to application. The FSAT approach to screen asymptomatic children and treat all inhabitants of households where ≥1 individual is found parasite positive by RDT has not been done routinely in Kenya but is an extension of screening and treatment of asymptomatic individuals that is proposed under the Kenyan National Malaria Strategy for 2009-2017[[13](#_ENREF_13)]. The national strategy does not currently propose to treat household members of asymptomatic parasite carriers; the approach is justified by evidence of clustering of parasite carriage at household level [2, 6, 25, 33] and the low sensitivity of RDTs for detecting all epidemiologically relevant parasite densities [[14](#_ENREF_14)]. We will provide supervised treatment with the first line antimalarial drug AL that has an excellent safety profile and is recommended for treatment of symptomatic and asymptomatic malaria infections in Kenya [[13](#_ENREF_13)]. We will also closely monitor and document any reported side effects of all interventions by questionnaires on all days of treatment and one week after treatment, IRS and ITN distribution.

d. Benefits to survey participants

All participants of the cross-sectional survey will benefit from knowing their haemoglobin level and whether they have a clinical malaria infection. Any participant with low haemoglobin will receive treatment for anaemia. All individuals who are diagnosed with a clinical malaria episode will be treated with AL according to Kenya national guidelines.

e. Risks to survey participants

This survey is of minimal risk to participants. Blood will be collected by fingerprick only using a sterile lancet, and the total amount of blood collected is very small (200-300μl); participants may experience only a small bruise at the site where we collect the blood. Blood draws will be carried out by trained medical or laboratory staff members to ensure these are done in as safe a manner as possible.

After screening, each person who is diagnosed with clinical malaria will be treated with first line malaria treatment recommended by the Ministry of Health, Kenya, AL. AL is considered a very safe drug except in pregnant women (where there may be possible risk to the developing foetus) and very young children (where safety data to support its use are not available).

To minimize drug exposure in these risk groups, children under 6 months of age will be excluded from the study, and pregnant women will receive alternative medication as outlined above.

We will take steps to ensure that each study participant’s personal information will be protected. All questionnaires will be labelled with the participant name and person ID. Filter paper samples and other samples will be labelled using a personal ID only, which will only be known to study staff. After the questionnaires are completed, they will be returned to the data management staff while the filter paper samples will be returned to the laboratory staff at KEMRI/CDC in Kisian. Once at the laboratory, only data management staff will possess the link between the laboratory samples and the participants’ identifiable information.

**13. Expected application of the results:**

This study will provide critical data on for malaria control programmes on how to best allocate resources for malaria control. If a hotspot targeted approach proves promising in the current cluster randomized trial, it has the potential to maximize the impact of malaria control efforts in all areas with heterogeneous malaria transmission.

**14. References:**

1. Clark TD, Greenhouse B, Njama-Meya D, Nzarubara B, Maiteki-Sebuguzi C, Staedke SG, Seto E, Kamya MR, Rosenthal PJ, Dorsey G: **Factors Determining the Heterogeneity of Malaria Incidence in Children in Kampala, Uganda**. *JInfectDis* 2008, **198**(3):393-400.

2. Bejon P, Williams TN, Liljander A, Noor AM, Wambua J, Ogada E, Olotu A, Osier FH, Hay SI, Farnert A *et al*: **Stable and unstable malaria hotspots in longitudinal cohort studies in Kenya**. *PLoS Med* 2010, **7**(7):e1000304.

3. Bousema T, Drakeley C, Gesase S, Hashim R, Magesa S, Mosha F, Otieno S, Carneiro I, Cox J, Msuya E *et al*: **Identification of hot spots of malaria transmission for targeted malaria control**. *J Infect Dis* 2010, **201**(11):1764-1774.

4. Kleinschmidt I, Schwabe C, Benavente L, Torrez M, Ridl FC, Segura JL, Ehmer P, Nchama GN: **Marked increase in child survival after four years of intensive malaria control**. *Am J Trop Med Hyg* 2009, **80**(6):882-888.

5. Aregawi MW, Ali AS, Al-mafazy AW, Molteni F, Katikiti S, Warsame M, Njau RJ, Komatsu R, Korenromp E, Hosseini M *et al*: **Reductions in malaria and anaemia case and death burden at hospitals following scale-up of malaria control in Zanzibar, 1999-2008**. *Malar J* 2011, **10**:46.

6. Gaudart J, Poudiougou B, Dicko A, Ranque S, Toure O, Sagara I, Diallo M, Diawara S, Ouattara A, Diakite M *et al*: **Space-time clustering of childhood malaria at the household level: a dynamic cohort in a Mali village**. *BMCPublic Health* 2006, **6**:286.

7. Smith DL, McKenzie FE, Snow RW, Hay SI: **Revisiting the Basic Reproductive Number for Malaria and Its Implications for Malaria Control**. *PLoS Biol* 2007, **5**(3):e42.

8. Woolhouse ME, Dye C, Etard JF, Smith T, Charlwood JD, Garnett GP, Hagan P, Hii JL, Ndhlovu PD, Quinnell RJ *et al*: **Heterogeneities in the transmission of infectious agents: implications for the design of control programs**. *ProcNatlAcadSciUSA* 1997, **94**(1):338-342.

9. Greenwood BM: **The microepidemiology of malaria and its importance to malaria control**. *TransRSocTropMedHyg* 1989, **83 Suppl**:25-29.

10. Bejon P, Cook J, Bergmann-Leitner E, Olutu A, Lusingu J, Mwacharo J, Vekemans J, Njuguna P, Leach A, Lievens M *et al*: **Effect of the pre-erythrocytic candidate malaria vaccine RTS,S/AS01_E_ on blood stage immunity in young children**. *Journal of Infectious Diseases* 2011, **in press**.

11. Ceesay SJ, Casals-Pascual C, Erskine J, Anya SE, Duah NO, Fulford AJ, Sesay SS, Abubakar I, Dunyo S, Sey O *et al*: **Changes in malaria indices between 1999 and 2007 in The Gambia: a retrospective analysis**. *Lancet* 2008, **372**(9649):1545-1554.

12. Drakeley CJ, Corran PH, Coleman PG, Tongren JE, McDonald SL, Carneiro I, Malima R, Lusingu J, Manjurano A, Nkya WM *et al*: **Estimating medium- and long-term trends in malaria transmission by using serological markers of malaria exposure**. *ProcNatlAcadSciUSA* 2005, **102**(14):5108-5113.

13. DOMC: **National Malaria Strategy 2009-2017: Towards a malaria-free Kenya**. In*.* Nairobi: Division of Malaria Control; Ministry of Public Health and Sanitation; 2009.

14. Okell LC, Ghani AC, Lyons E, Drakeley CJ: **Submicroscopic infection in Plasmodium falciparum-endemic populations: a systematic review and meta-analysis**. *J Infect Dis* 2009, **200**(10):1509-1517.

15. Steenkeste N, Rogers WO, Okell L, Jeanne I, Incardona S, Duval L, Chy S, Hewitt S, Chou M, Socheat D *et al*: **Sub-microscopic malaria cases and mixed malaria infection in a remote area of high malaria endemicity in Rattanakiri province, Cambodia: implication for malaria elimination**. *Malar J* 2010, **9**:108.

16. Corran PH, Cook J, Lynch C, Leendertse H, Manjurano A, Griffin J, Cox J, Abeku T, Bousema T, Ghani AC *et al*: **Dried blood spots as a source of anti-malarial antibodies for epidemiological studies**. *Malaria Journal* 2008, **7** 195.

17. Bousema T, Youssef RM, Cook J, Cox J, Alegana VA, Amran J, Noor AM, Snow RW, Drakeley C: **Serologic markers for detecting malaria in areas of low endemicity, Somalia, 2008**. *Emerg Infect Dis* 2010, **16**(3):392-399.

18. World Health Organization GS: **Malaria Rapid Diagnostic Test Performance - results of WHO product testing of malaria RDTs: Round 1 (2008). 10.2471/TDR.09.978-924-1598071**. In*.*; 2009.

19. Griffin JT, Hollingsworth TD, Okell LC, Churcher TS, White M, Hinsley W, Bousema T, Drakeley CJ, Ferguson NM, Basanez M-G *et al*: **Strategies towards Plasmodium falciparum malaria elimination in Africa using currently available tools**. *PLoS Med* 2010, **6**:e20179.

20. Hawley WA, Phillips-Howard PA, ter Kuile FO, Terlouw DJ, Vulule JM, Ombok M, Nahlen BL, Gimnig JE, Kariuki SK, Kolczak MS *et al*: **Community-wide effects of permethrin-treated bed nets on child mortality and malaria morbidity in western Kenya**. *AmJTropMedHyg* 2003, **68**(4 Suppl):121-127.

21. Fillinger U, Kannady K, William G, Vanek MJ, Dongus S, Nyika D, Geissbuhler Y, Chaki PP, Govella NJ, Mathenge EM *et al*: **A tool box for operational mosquito larval control: preliminary results and early lessons from the Urban Malaria Control Programme in Dar es Salaam, Tanzania**. *Malar J* 2008, **7**:20.

22. Fillinger U, Ndenga B, Githeko A, Lindsay SW: **Integrated malaria vector control with microbial larvicides and insecticide-treated nets in western Kenya: a controlled trial**. *Bull World Health Organ* 2009, **87**(9):655-665.

23. Smith DL, Dushoff J, Snow RW, Hay SI: **The entomological inoculation rate and Plasmodium falciparum infection in African children**. *Nature* 2005, **438**(7067):492-495.

24. Brooker S, Kolaczinski JH, Gitonga CW, Noor AM, Snow RW: **The use of schools for malaria surveillance and programme evaluation in Africa**. *Malar J* 2009, **8**:231.

25. Stresman GH, Kamanga A, Moono P, Hamapumbu H, Mharakurwa S, Kobayashi T, Moss WJ, Shiff C: **A method of active case detection to target reservoirs of asymptomatic malaria and gametocyte carriers in a rural area in Southern Province, Zambia**. *Malar J* 2010, **9**:265.

26. Okell LC, Griffin J, Kleinschmidt I, Hollingsworth TD, Churcher T, White M, Bousema T, Drakeley CJ, Ghani A: **The potential contribution of mass treatment to the control of Plasmodium falciparum malaria.** *PLoS One* 2011, **6**(5):e20179.

27. Drakeley CJ, Corran PH, Coleman PG, Tongren JE, McDonald SL, Carneiro I, Malima R, Lusingu J, Manjurano A, Nkya WM *et al*: **Estimating medium- and long-term trends in malaria transmission by using serological markers of malaria exposure**. *Proceedings of the National Academy of Science, USA* 2005, **102**(14):5108-5113.

28. Poinsignon A, Cornelie S, Mestres-Simon M, Lanfrancotti A, Rossignol M, Boulanger D, Cisse B, Sokhna C, Arca B, Simondon F *et al*: **Novel peptide marker corresponding to salivary protein gSG6 potentially identifies exposure to Anopheles bites**. *PLoS ONE* 2008, **3**(6):e2472.

29. Hsiang MS, Lin M, Dokomajilar C, Kemere J, Pilcher CD, Dorsey G, Greenhouse B: **PCR-based pooling of dried blood spots for detection of malaria parasites: optimization and application to a cohort of Ugandan children**. *J Clin Microbiol* 2010, **48**(10):3539-3543.

30. Hayes RJ, Bennet SJ: **Simple sample size calculations for cluster-randomized trials**. *Int J Epidemiol* 1999, **28**:319-326.

31. MOH N: **National guidelines for HIV-testing and counseling in Kenya (draft)**. 2007.

32. Adazu K, Feiken, D., Ofware, P., Onyango, B., Laserson, K., Vulule, J. : **Migration and Child Mortality in rural Nyanza Province; Results from KHDSS in Western Kenya**. In: *Population Association of America Annual Meeting* New Orleans; 2008.

33. Pullan RL, Kabatereine NB, Bukirwa H, Staedke SG, Brooker S: **Heterogeneities and consequences of Plasmodium species and hookworm coinfection: a population based study in Uganda**. *J Infect Dis* 2011, **203**(3):406-417.

34. Brooker S, Okello G, Njagi K, Dubeck MM, Halliday KE, Inyega H, Jukes, MC: **Improving educational achievement and anaemia of school children: design of a cluster randomised trial of school-based malaria prevention and enhanced literacy instruction in Kenya.** *Trials* 2010, 11:93.

35.Bousema JT, Schneider P, Gouagna LC, Drakeley CJ, Tostmann A, Houben R, Githure JI, Ord R, Sutherland CJ, Omar SA, Sauerwein RW: **Moderate effect of artemisinin-based combination therapy on transmission of Plasmodium falciparum.** *J Infect Dis*. 2006 , 193:1151-9.

36. Okell LC, Drakeley CJ, Bousema T, Whitty CJ, Ghani AC: **Modelling the impact of artemisinin combination therapy and long-acting treatments on malaria transmission intensity.** *PLoS Med*. 2008. 5:e226.

**15. BUDGET**

(1 US$ = 90 KSH)

|  |  | Annual cost | Annual cost |
| --- | --- | --- | --- |
|  |  | KSh | $US |
|  | Project Personnel salaries and benefits | 16,520,000 | 183,556 |
| **Intervention costs** | | | |
| FSAT | | | |
|  | Field workers | 682,000 | 7,578 |
|  | Equipment, supplies, drugs | 898,000 | 9,978 |
|  | Travel | 2,437,500 | 27,083 |
| LLINs | | | |
|  | Field workers | 682,000 | 7,578 |
|  | Equipment, supplies, bednets | 7,040,000 | 78,222 |
|  | Travel | 2,437,500 | 27,083 |
| IRS | | | |
|  | Field workers | 1,364,000 | 15,156 |
|  | Equipment, supplies, insecticide | 860,000 | 9,556 |
|  | Travel | 4,920,000 | 54,667 |
| Larviciding | | | |
|  | Field workers | 808,000 | 8,978 |
|  | Equipment, supplies, larvicide | 30,500 | 339 |
|  | Travel | 3,510,000 | 39,000 |
| **Survey costs** | | | |
|  | Field workers | 3,492,000 | 38,800 |
|  | Equipment, supplies, insecticide | 2,400,000 | 26,667 |
|  | Travel | 7,380,000 | 82,000 |
| **Laboratory Costs** | | | |
|  | PCR | 1,044,000 | 11,600 |
|  | Serology | 296,910 | 3,299 |
| **Others** | | | |
|  | Animals | 0 | 0 |
|  | Consultancy fees | 0 | 0 |
|  | Contingency funds | 450,000 | 5,000 |
|  | Institutional administrative overheads | 8,587,862 | 95,421 |
|  | **Total** | **65,840,272** | **731,559** |
|  |  |  |  |

**16. BUDGET JUSTIFICATION**

**Personnel salaries and benefits.** Funds included here include salary cover for the period of the intervention and follow-up studies (including preparation and analysis) for all Kisumu-based staff. Staff include the study coordinator, clinical officer, data specialist, administrative and logistical support, entomology supervisor and technicians, laboratory technologists and community health workers.

Costs are broken down in to **intervention costs** and **survey costs.** Under each intervention field workers, equipment, supplies and any drugs or insecticides required are given. These encompass the following costs as detailed below:

Field workers This includes all salaries for casual staff during the particular study, such as community health workers and guides.

**Equipment, supplies, drugs/insecticides**. This is estimated to include sprayers, COPACK haemoglobin kits, nets and small laboratory equipment including thermometers, scissors, staining jars etc. Supplies covers the estimated cost of all the non-field work supplies required for the study. It includes photocopying, stationary, communication and printing over the study period. Drugs/insecticides covers costs of RDTs, Coartem, SP+AQ and ferrous sulphate sufficient to treat the estimated number of surveyed individuals for each stage as well as the insecticides required for IRS and larviciding.

**Travel and transportation**. This includes local travel and international travel of LSHTM staff to Kenya. All local travel costs during survey preparation and implementation are included. All costs for local travel and accommodation have been estimated using standard KEMRI mileage and per diem charges.

**Laboratory costs** This covers the costs of processing all blood samples collected for PCR and serological analyses.

Note: some **contingency costs** are included in this budget; additional contingency funds are available through wider Malaria Transmission Consortium budgets. Institutional overheads are levied at 15%.

**17. Roles investigators**

**Teun Bousema** is the Principal Investigator for the study and is a senior lecturer at both LSHTM, UK and Radboud University Nijmegen, The Netherlands. He will provide regular scientific support and supervision, help in design of the study and analysis of results and will participate in manuscript preparation.

**Nabie Bayoh** is the head of entomology at KEMRI/CDC research Station, Kisumu. He will assist with study design related to the larviciding intervention.

**Jonathan Cox** is a senior lecturer at LSHTM, UK. He will provide regular scientific support, will coordinate sampling strategies, and provide advice on spatial and statistical analysis.

**Meghna Desai** the Scientific Technical Advisor, Malaria Branch at KEMRI/CDC Kisumu and will provide scientific support and participate in manuscript preparation

**Chris Drakeley** is a Principal Investigator on this study and a senior lecturer at LSHTM, UK. He will provide support for serological analysis and will oversee training in this area. He will also provide general scientific support, data analysis and will participate in manuscript preparation

**Ulrike Fillinger** is a senior lecturer at LSHTM and senior scientist at the International Centre for Insect Physiology & Ecology (ICIPE) in Mbita, Kenya. She will assist with study design related to the larviciding intervention.

**Elizabeth Juma** is the working with KEMRI, Nairobi. She will provide guidance in the selection and planning of intervention tools, she will participate in the evaluation, analysis interpretation and manuscript preparation

**Simon Kariuki** is the Malaria Branch Chief at the KEMRI/CDC Research Station, Kisumu. He will provide on-site support for the survey and molecular analysis as well as participate in manuscript preparation.

**Kayla Laserson** is the KEMRI/CDC Field Research Station Director in Kisumu. She will provide regular scientific support and supervisory roles and will participate in manuscript preparation.

**Elizabeth Marube** is the lead clinical officer on the study team at KEMRI/CDC. She will advise on all clinical aspects of the work, monitoring drug treatment, reporting on adverse events and addressing any concerns raised by participants.

**Chrispin Owaga** is the Study Coordinator for the work at KEMRI/CDC, Kisumu. He will help with supervising teams, logistics and coordination of field activities.

**Jennifer Stevenson** is a Research fellow with LSHTM and Project Manager for the study in Rachuonyo. She will help with study design, manuscript preparation and will coordinate the interventions and cross-sectional studies.

**John M. Vulule** is the director of KEMRI CGHR and will provide regular scientific support and supervisory roles for both field and laboratory activities. He will participate in manuscript presentation.

***Scientific capacity building:***

Chrispin Owaga will be using part of the data collected during this project as part of his MSc project. Elizabeth Marube will be using this study to further improve her clinical and research skills and will, together with Chrispin Owaga and the more senior Kenyan staff members, be involved in the process of data analysis, manuscript preparation and publication. A student laboratory technician is currently in the process of submitting a proposal to carry out serological analyses from samples collected as part of her undergraduate project.

As part of this project, several laboratory technicians are currently being trained in molecular and serological assays at KEMRI/CDC Kisumu.

In general, the results from this will be made available to the head of the malaria branch Simon Kariuki and the director John Vulule in order to better inform the research centre and MOH about the options for targeted malaria control.

**Appendix A: Information sheets**

**A1: Study Information Sheet for District Representatives**

**English**

Reducing the burden of malaria by targeting hotspots of transmission and improving malaria control measures in the highlands of Western Kenya: Simultaneous rollout of four malaria control interventions and evaluation by cross-sectional surveys.

**Organizations involved:** Ministry of Health and Division of Malaria Control, Kenya, London School of Hygiene and Tropical Medicine UK (LSHTM), Radboud University Nijmegan, & KEMRI/CDC Kenya

**PIs:** Dr. Teun Bousema (LSHTM, Radboud University Nijmegan), Dr Jonathan Cox, Dr Chris Drakeley (LSHTM)

**Field Team Supervisors:**

| Dr. Jennifer Stevenson | Project Manager | 0713-510845 |
| --- | --- | --- |
| Gillian Stresman | Epidemiologist/Field Manager | 0713-880159 |
| Amrish Baidjoe | Molecular Immunologist /Field Manager | 0704-131154 |
| Chrispin Owaga | Study Coordinator | 0724-273318 |
| Diana Okello | Administrative Officer | 0720-801542 |
| Elizabeth Marube | Clinical Officer | 0722-282097 |
| Robin Oriango | Lab Tech/ Field Supervisor | 0721-823215 |
| Albert Okoth | Lab Tech/ Field Supervisor | 0721-824882 |
| Wycliffe Odongo | Data Manager | 0724-694426 |

**Aim:** To evaluate the impact of targeted malaria control interventions in communities with a high prevalence of malaria in the highland, unstable transmission areas in Rachuonyo.

**Background and justification**

Campaigns using massive distribution of bednets and indoor residual spraying by the Ministry of Health in Kenya have made significant progress in reducing malaria transmission. Despite these measures, malaria remains a problem in some rural communities. Studies carried out in 2011 in the epidemic prone highland areas of Rachuonyo indicated that there are areas that remain with higher malaria prevalence than the surrounding communities.

To reduce malaria transmission in the highlands of Rachuonyo we plan on targeting these malaria ‘hotspots’. Malaria hotspots are important as they may be areas where infections are maintained from one transmission season to the next and serve as the reservoir from which infections spread. Targeting interventions to those areas where malaria prevalence is higher at the time when transmission is low may allow for a more cost-effective way of reducing malaria in the whole community. Therefore we plan to study the effects of providing 4 different types of malaria control; 1) indoor residual spraying 2) provision of long-lasting bednets 3) larviciding of all breeding sites 4) focal screening and treatment of malaria positive households. We plan to provide these malaria interventions in up to 8 hotspots within Rachuonyo highlands and then compare malaria prevalence through community surveys in and around these areas to hotspots that do not receive the interventions. By using a combination of all these interventions at the time when we expect to see the biggest impact, we hope to reduce malaria transmission both inside the hotspots and the surrounding areas.

This protocol falls under the larger Malaria Transmission Consortium (MTC) umbrella of projects that aims to investigate the effectiveness of programmatic activities against malaria and to develop standardized methods of estimating malaria transmission in different epidemiological settings. As such this protocol complements those already on-going activities evaluating malaria control interventions and together they will provide critical information to the DOMC on the most effective malaria control strategies in highland areas of Western Kenya.

**Design and Methodology**

Study site: We would like to carry out the survey in highland Rachuonyo in rural areas with an average altitude of between 1400m and 1600m. The study sites will be in homesteads that fall within South Rachuonyo in a 5km by 25km area centred around Ringa (see map attached). In this area bednet coverage is about 60-70% and IRS campaigns have been running annually since 2008.

Selection of participants: In June-July 2011 satellite imagery of the area was acquired and the 5km x 25km area was divided into 500m x 500m units. Identified compounds were screened and tested for malaria. Using this grid, data maps were created showing the distribution of malaria in the community. These maps were used to identify communities that had a higher prevalence of malaria than others (i.e. hotspots). Of these hotspots, up to 16 will be selected and half will be randomly assigned for receiving the interventions. All compounds in the selected hotspots will be visited and offered the interventions. For the evaluation surveys up to 200 people inside each hotspot and up to 500 people living in the area surrounding each hotspot will be randomly selected to participate. We hope to carry out three surveys per year. The first survey will take place at the beginning of the transmission season (March), the next is planned for the peak of the transmission season (July), and the final study at the end of the transmission season (September). This study is expected to run for 2 years.

Intervention procedure: Prior to implementation communities, households and individuals will be visited to obtain consent. Roll-out of interventions will begin in March 2012.

*Indoor residual spraying:* The insecticide that will be used for IRS will be decided on following consultations with DOMC and will be one of the following: lambdacyhalothrin, alphacypermethrin or deltamethrin. Activities and actual spraying will be undertaken by study personnel; IRS will be timed before the peak transmission season, at the time-point that is deemed most efficacious by comprehensive malaria transmission models [19] and again after 6 months. The study team will remove all movable items from the structures that will be sprayed. Any large items that cannot be removed will be moved into the centre of the room and covered with plastic sheets. Sprayers will dispense the insecticide using a back pack sprayer. The walls and roof of all structures in the compound (residential and non-residential) will be sprayed for at least 15 minutes according to guidelines for the insecticide used. The structures will be left locked for 2 hours after which the doors and windows will be opened and allowed to air out for 30 minutes. After this the head of the house may enter to sweep clean. Spraying will be repeated at 6-monthly intervals.

*Long-lasting insecticide treated nets (LLIN) distribution:* Prior to roll-out, study staff will visit households to document the number of sleeping spaces within each house. At the time of the intervention (March 2012), the study team will distribute one LLIN for each sleeping space within the house, hang the net and explain the proper use and maintenance of the net. To avoid potential irritation to the insecticide used in the LLIN, nets will be hung outside for at least 24 hours before being distributed to communities to allow the strength of the insecticide to wane. After 1 month, the correct usage of LLINs will be evaluated. If needed, instructions about correct use will be repeated and additional nets can be provided if nets are missing or found to be torn. A third visit will occur to re-assess the quality and use of the nets 6 months after the first visit, in conjunction with the second round of IRS. At this point new ownership and quality will be re-assessed and new nets will be distributed as needed.

*Focal Screening and Treatment:* In intervention hotspots temperatures will be taken from everyone using an ear thermometer and blood samples will be collected by fingerprick. For those individuals aged between 6 months and 20 years the blood will be used to test for malaria by rapid diagnostic test (RDT) and for determination of haemoglobin levels, to prepare a microscopy slide and to collect blood into a small tube and onto a filter paper. These samples will be analysed in laboratories in Kisumu for parasite prevalence. If one or more participant between 6 months and 20 years are parasite positive, it is likely that household members are also infected. For individuals aged above 20 years, only those that are febrile will be tested for malaria using RDTs otherwise blood will be collected as described above for the same purposes. The volume of the blood sample taken for all measurements is 1/10 of a tea spoon. If an individual is found to be infected with malaria as shown by the RDT all members of that household, unless pregnant, will be treated using Coartem according to national guidelines. Coartem is not recommended for pregnant women. If a women of childbearing age (13-45 years) is found positive for malaria or lives in a house where another member is positive, a female health worker will approach them to assess whether they could be pregnant. This will be assessed through the use of a questionnaire and, if necessary and accepted by the woman, a urine test. If found to be pregnant or not willing to have a urine test, the woman will be taken to a nearby health facility where they can be examined and appropriate treatment given before being returned home. All costs will be covered by the project. As part of the supervisory team a qualified Clinical Officer will be available to advise on all treatment regimes and any side effects from drugs taken. All participants will also be told their haemoglobin level immediately, and any person found to be anaemic will be given iron according to national guidelines.

Survey procedure: A team of trained field workers will locate those identified for inclusion in the study. The team will introduce the study to all participants (and to parents of children if under the age of 18) and seek consent or assent from adults and from parents or guardians of children who are willing to participate, and who are over 6 months of age. Children under 6 months of age will be excluded from the study. This is because Coartem, the drug that will be used to treat malaria is not recommended for young infants.

The head of household or primary caretaker(s) will be interviewed on

- Household construction materials
- Geographical locators (e.g. nearest school, nearest market)
- Use of anti-malaria measures (the use of bednets, recent IRS within houses)

Results will be recorded using a personal digital assistant (PDA). Field workers will measure temperatures using an ear thermometer for each individual within the compound. For those found to have fever they will be tested for malaria using a RDT. All individuals whether febrile or not will also have a fingerstick blood sample taken using a lancet. This will be used to establish if they are anaemic (low blood levels) using a HemoCue machine and to collect blood samples for subsequent tests for malaria in the labs in Kisumu. The tests carried out will look for antibodies to malaria and DNA of malaria parasites. These samples will not be used to test for any other diseases.

RDT results and blood level results will be immediately available and recorded on PDAs. For those who are found to be positive for malaria by RDT they will be treated with Coartem according to National Guidelines. As described above women who are suspected to be pregnant cannot be treated with Coartem and so will be taken to nearby health facilities for further treatment.

For those found to be anaemic, iron or Ferro B will be administered. If participants are found to be severely anaemic they will be referred to the closest health facility. If participants are found to be extremely ill for any reason they will also be referred. If the project vehicle can reach the participant quickly they will be taken to the nearest health facility or hospital as is deemed appropriate.

**Treatment/referral administered on site and benefits for the community**

- All participants will be informed whether they have a fever
- Those who are febrile will receive a malaria test free of charge and know whether they have malaria infection immediately
- For those with a positive RDT and who are not less than 6 months or pregnant will be given Coartem for free
- Everyone will be offered a test for anaemia and the results made available immediately
- Anyone with low haemoglobin will receive free iron treatment for anaemia
- Anyone found to be severely ill will be referred to a health facility and if possible transport provided
- All results from the survey will be passed onto the DC, DOs, DMOHs and DPHOs as well as the chiefs and communities involved following completion of the project
- All project reports will be submitted to DOMC, Nairobi

**Mamoko A: Oboke mag tiend weche**

**A1: Oboke Mar Tiend Wach Nonro mar Jochung’ Mag Distrik**

**Dholuo**

Duoko pek mar maleria piny ka idimbo dago ma landruok tuo ng’enyie kod jiwo yore mag mag gayo malerie e hosi mag gode man milambo mar Kenya: Riwo yore ang’wen mag gayo maleria kod nonro margi ma dichiel.

**Migepe ma nitie:** Migao mochung’ ne gayo maleria, Kenya, Kar Tiegruok ma London mar Ler kod Tuoche malandore UK (LSTMH), Mbalariany mar Radboud Nijimegan, kod KEMRI/CDC Kenya.

**Jotend nonro:** Lkt. Teun Bousema (LSHTM, Radboud University Nijmegan), Lkt. Jonathan Cox, Lkt. Chris Drakeley (LSHTM)

**Jotend tich mar pap:**

| Lkt. Jennifer Stevenson | Jatelo maduong’ | 0713-510845 |
| --- | --- | --- |
| Gillian Stresman | Jalony mar kute mafuyo/Jarit pap | 0713-880159 |
| Amrish Baidjoe | Jalony mar kaka del gayo tuoche/Jarit pap | 0704-131154 |
| Chrispin Owaga | Ogaye mar nonro | 0724-273318 |
| Diana Okello | Jachung’ mar tayo ofis nonro | 0720-801542 |
| Elizabeth Marube | Laktar mar touche dhano | 0722-282097 |
| Robin Oriango | Jalony e tije lab/ Nyapara mar pap | 0721-823215 |
| Albert Okoth | Jalony e tije lab/ Nyapara mar pap | 0721-824882 |
| Wycliffe Odongo | Jalony mar data (weche mawuok e nonro) | 0724-694426 |

**Gima omiyo itimo nonro:** Dwaro fwenyo dwoko mar yore moketi maratiro mag gayo maleria e gwenge ma maleria ng’enyie ewi gode, ma landruok maleria ok osiko dhiyoe mbele, ei Rachuonyo.

**Tiend nonro kod gima omiyo itime**

Temo gayo maleria ka itiyo kod chiwo nede mag suna mathoth kod goyo yath ei udi gi Migao mar Ngima Dhano ma Kenya (Ministry of Health) osekelo yuto maduong’ kuom duoko piny landruok mar maleria. Kata kamano, maleria pod kelo chandruok maduong’ ne jok modak e kor gwenge. Nonro mosetim e hik 2011 e hosi mag wig ode ma Rachuonyo ma maleria japowore ahinya ne onyiso ni nitie kuonde ma maleria mapod odong’ gi maleria mang’eny moloyo gwenge molworogi.

Mondo oduok piny landruok maleria ewi gode ma Rachuonyo, wachano dimbo kuonde ma nitie ‘dago maleria’ gi. Kuondegi nitie gi pek ahinya nikech ginyalo bedo ni gin e kuonde ma maleria pondoe e kind thuolo ariyo mag landruok mapek, kendo chalo kaka dero ma maleria landore ka oae. Dimbo kuonde ma kama ma nitie gi maleria mang’eny e kinde ma otin kuonde molworogi gi yore mag gayo maleria nyalo kelo yo ma yot mar dwoko maleria piny e oganda ma lach. Kuom mano, wadwaro rango duoko mar tiyo kod yore 4 mag gayo maleria; 1) goyo yath madak ei ot 2) chiwo net mar suna ma dak 3) olo yath kuonde ma suna nyuolore duto 4) pimo kod thieth ma oyiero ute ma nitie joma nigi maleria. Wachano chiwo yoregi e dago maleria 8 ei Rachuonyo, bang’e to wapimo ng’eny maleria koluwa nonro mar kor gwenge ei kod e aluoragi, to kod dago ma ok oyudo yore gayo maleria. Kuom tiyo gi yoregi kanyakla e kinde ma wageno neno tekogi maber mogik, wageno duoko landruok maleria piny ei dago maleria kod aluora margi.

Nonroni nite e bwo riwruok mar nonro maduong’ marango landruok mar maleria (MTC), madwaro fwenyo yore mongirore mag gayo maleria kod temo yore mopuodhi mag pimo landruok mar maleria kuonde mopogore opogore. Kuom mano, nonroni riwore kod mamoko madhi nyime ewi yore mag gayo maleria, kendo kanyakla gibiro kelo fweny ne migao mochung’ne gayo maleria kuom yore mabeyo mogik mag gayo maleria e milambo mar Kenya.

**Yore ma nonro luwo**

Kama itimoe nonro: Wadwaro timo nonroni e gwenge ma ewi gode ma Rachuonyo ma bor mare kowuok e hosi mar pi wi nam en gimoro mita 1400 nyaka mita 1600. Nonro biro timore e udi man Rachuonyo ma **Nyanduat** e aluora maromo kilomita 5 konchiel kod kilomita 25 e hosi mar Ringa (ne goro mar map mochiw cha). E gwengegi, net mar suna yudore e atamalo 60-70 (kuom mia) kendo okange mag goyo yadh suna ei udi osebedo ka dhi nyime higa ka higa chakre hik 2008.

Yiero jokanyo mag nonro: E thuolo mar deu mar 6 nyaka 7 e hik 2011 picha mar setlait ne ogo bang’e eria mar nonro ma pim mare en kilomita 5 x kilomita 25 ne opogi e hosi matindo tindo madirom mita 500 x mita 500. Delni mane oyudi ne okaw kendo otimie pim mar maleria. Ka iyo gi picha ni, ne otim goro manyiso kaka mier ma maleria nitie ne obedo. Goro mag mabegi ne oti godo kuom piedho gwenge mane nitie kod maleria mang’eny moloyo moko (kata dago). Kuom dagogi, maromo 16 ibiro yier kendo nusgi ibiro kwany radharadha mondo odimbgi kod yore mag gayo maleria. Delni duto matie ei dago mokwanygi ibiro ketie yore mag gayo maleria. Kuom ng’iyo kaka yoregi tiyo, ji 200 ei dago ka dago kod ji 500 e aluora mar dago ka dago ibiro kwany radharadha mondo odonji e nonro. Wageno ng’iyo kaka yoregi tiyo nya didek higa ka higa. Mokwongo wabiro timo e kinde ma landruok mar maleria chakore (dwe mar adek), maluwe opang timo e kinde ma landruok biro ng’enyie ahinya (dwe mar abiriyo), kendo mogik ibiro tim tok kinde ma landruok biro yudo orumoe (dwe mar ochiko). Nonro ni igeno ni biro dhi nyime kuom higni ariyo.

Kaka wabiro keto okange mag gayo maleria: Ka pok wachako, ogendni, udi kod ji ibiro lim mondo okwai ayie mar donjo e nonro. Keto kange mag gayo maleria biro chakoro dwe mar Adek, 2012.

*Goyo yadh suna ei udi:* Yadh suna ma ibiri ti godo kuom goyo yath madak ei udi ibiro yier bang’ lalruok kod migao mochung’ ne gay maleria Kenya (DOMC). Dich kod goyo yath owuon ibiro tim kod jogo ma tiyo e nonroni; goyo yath ibiro tim ka pok kinde mag landruok maleria mang’eny mogik ochopo, e thuolo ma ber mogik kaluwore gi nonro mag landruok maleria mosekalo (19) kendo bang’ dweche auchiel. Jogo matayo nonro biro golo oko gik manyalo sudo oko mar ot ma igoyoe yath. Gigo duto madongo ma ok nyal gol oko ibiro choki e chuny die rot kendo ibiroum kod jwala. Jok magoyo yath biro timo kamano ka gitiyo kod rating’ yath ma iting’o e tok. Kor udi kod tat udi duto ma nitie e dala (ma ji dakie kod ma ok mag dak) ibiro goye yath kuom thuolo ma ok tin ne dakika 15 kaluwore kod yath ma itiyogo. Udigi biro dong’ ka olor makare kuom seche 2 bang’e dhoudi kod dirisni ibiro yaw kendo we thuolo kuom dakika 30. Bang’ ma wuon ot biro bedo thuolo donjo yweyo ot. Goyo yath biro timore dweche auchiel auchiel.

*Pogo ned suna mantie gi yath madak:* Ka pok wachako keto okangegi, jotaa nonro biro limo udi mondo giyud kwan mar kuonde nindo ei ot ka ot. E kinde mar chako okangegi (dwe mar Adek 2012), jota nonro biro pogo net achiel kuom kar nindo ka kar nindo ei ot, tweyo nedno kendo lero yo malong’o mar tiyo kendo rito nedno. Mondo ogeng’ ilo del ma nyalo wuok kuom yath ma oketi e net, nede ibiro mo oko kuom thuolo mohingo seche 24 ka pok opogi ogendni mondo omi teko tath odog piny. Bang’ dwe achiel, ibiro non ka bende tiyo gi net tiomore e yo makare. Ka dwarore, lero ibiro chiw kendo ewi nedegi kendo nede mamoko chiw ka oyud ka nede olal kata oyiech. Limbe mar adek ibro tim mondo onon kendo tiyo kod net dweche 6 bang’ limbe mokwongo, ka bende igoyo yath mar ariyo. E kindeni weg net manyien kod tiyo maber gi nede ibiro non kendo, ka ichiwo nede manyien kaka dwarore.

*Pimo kod thieth ma odimbi:* E dago ma yore gayo maleria biro timoree, liet mar del ibiro pim kuom ji duto ka itiyo gi rapim liet ma iketo e it kendo remo matin ibiro gol ewi lith lwedo. Kuom jogo ma hikgi ni e kind dweche 6 kod higni 20, remo ibiro tii godo e dwaro fwenyo ka ng’ato nigi maleria ka oluwo yo mapiyo ahinya (RDT) kod nono ng’eny mar remo e del, loso sampu a kiyo ma itiyogo e nyong’ong’ mafweyo gik matindo ahinya, kendo tiyo gi fururu matin ahinya e keto remo e kalatas moro makende. Sampu gi ibiro rang godo e lab man Kisumo ka ng’ato nitie gi kute makelo maleria. Ka ng’ato kata ji mohingo achiel ma hikgi nitie kind dweche 6 gi higni 20 nitie gi kutegi to nenore ni jomoko ei odno bende nitie gi kutegi. Kuom jogo ma hikgi ohingo 20, mana jok ma dend gi liet mohingo kaka dwarore ema ibiro pimnegi maleria ka itiyo gi RDT kata kamano remo to ibiro kaw kaka ondiki malo ka ka itiyogo bende machal. Remo ma ibiro kaw biro room 1/10 mar ojiko mar chae. Ka ng’ato oyudi gi kute mag maleria to ji duto manie odno ibiro thiedhi gi yadh Coartem mak mana mine mapek, kaluwore gi chik mar sirikal. Coartem ok opuodhi tiyogo kuom mine ma igi odongo. Ka dhako ma hike nitie e kinde ma onyalo nyuolie (higni 13-45) oyudi gi kute mag maleria kata odak ei ot ma oyudie ng’ato gi kute maleria, jatich migao mar thieth ma dhako biro chopo irgi mondo opim ka di gibed ni igi odongo. Ma ibiro tim ka iluwo penjo ma ondiki, kendo ka giyie, to ibiro pim lach. Ka oyudi ni iye odongo kata ka ok odwar ni opim lachne, ibiro tere e od thieth machiegni mogik kama inyalo pimgie kendo thieth makare chiwnigi ka pok oduokgi dala. Garama duto ibiro chung’ie kod nonro. Achiel kuom nyapara mantie e gwenge saa nonro biro bedo laktar mar ngima dhano, mabiro bedo machiegni ka ochiwo ler ewi thieth makare kod gigo manyalo wuok e yien ma itiyogo.

Yore mag nonro: Jogo motiegi e tije kor gwenge biro manyo jok ma oseyier mondo odonji e nonro. Jok motiegi gi biro chiwo ler makare ne jogo madonjo e nonroni duto (kendo ne jonyuol nyithindo ma hikgi ok rom 18), kendo kwayo ayie mar joma dongo kod jonyuol kata jorit nyithindo ma oyie donjo e nonro, kendo hikgi ohingo dweche 6. Nyithindo ma hikgi ok rom dweche 6 ok bi rwaki e nonro. Ma en nikech Coartem ma ibiro thiedh godo maleria ok opuodhi e thiedho nyithindo ma hikgi or rom dweche 6 .

Wuon ot kata ng’atno/jogo matayo ot/udi ibiro penji ler ewi

- Gigo ma oger godo ot
- Gigo ma nyalo nyiso dala (kaka sikul machiegni mogik, chiro machiegni mogik)
- Tiyo gi yore magayo maleria (nede, goyo yadh suna mokalo mogik)

Duoko ibiro ndiki ka itiyo gi gimoro ma sani ma iluongo ni PDA. Jogo matiyo e gwenge biro pimo liet mar dend ji duto mantie e dala ka gitiyo kod gir pimo liet ma iketo e it. Jogo ma oyud ni dendgi liet mohingo okang’ makare ibiro pim maleria kuomgi ka itiyogi RDT. Ji duto ma dendgi liet kata ok liet ahinya biro chiwo remo mar wi lieth lwedo. Ma ibiropim godo ng’eny mar rembgi ka itiyo gi HemoCue, kendo kuom kawo remo ma ibirotim godo pim mamoko mag rango maleria e lab ma Kisumo. Pim mitimogi biro dwaro gigo ma del tiyogo geng’o maleria (antibody) kod DNA mar kute maleria. Remogi ok bi ti godo e pimo tuoche mamoko.

Duoko mag RDT kod ng’eny mar remo biro wuok gi kanyo kendo ibiro rwaki e PDA. Jogo ma oyudi gi maleria kokalo e RDT ibiro thiedhi gi Coartem kaluwore gi chike mag sirikal. Kaka osewachi malo ka, mine ma ichicgo ni igi odongo ok nyal thiedhi gi Coartem omiyo ibiro tergi kuonde thieth machiegni mogik ne thieth makare.

Ne jogo ma oyudi gi remo matin, yeth ma iluongo ni Ferro B ibiro chiw. Ka oyudi ni remo tin ahinya to orgi e od thieth machiegni mogik. Ka jok mantie e nonro oyud ka tuo ahinya nikech gimoro amora to gin bende ibiro orgi e kuonde thieth mantie machiegni kodgi. Ka gari mar nonro nyalo tero jatuo mapiyo to ibiro tergi e od thieth machiegni mogik, kaka dwarore.

**Thieth/oro jatuo e do thieth ma ichiwo e gweng’ kod ber mare ne oganda**

- Jogo duto matie e nonro ibiro nyisi ka gin gi del maliet kata ka giongego
- Joma dendgi liet ibiro pim maonge chudo kendo biro ng’eyo gikanyo ka gin kod maleria
- Joma RDT onyiso ni nitie gi maleria ma hikgi ok tin ne dweche 6 to bende onge ich ibiro thiedhi gi Coartem maonge chudo
- Ji duto biro yudo pim mar ng’eny mar remo kendo yudo duoko gikanyo
- Ng’ato ang’ata ma rembe tin biro yudo thieth maduoko remo maonge chudo
- Ng’ato ang’ata ma tuo ahinya ibiro or e ute thieth, kendo inyalo kowgi kuro ka nyalore
- Duoko duto ibiro orne DC, DO, DMOH kod DPHO, nyaka ne ruodhi kod ogendni mobedoe nonro, tok rumo mar nonro.
- Ripode duto mag nonroni ibiro orne migao mar gayo maleria, DOMC, Nairobi

**Nyongeza A: Maelezo**

**A1: Maelezo juu ya utafiti kwa Waakilishi wa Wilaya**

**Kiswahili**

Kupunguza uzito wa ugonjwa wa malaria kwa kuelekeza juhudi mahali palipo ueneaji wa juu na kuimarisha juhudi za kupigana na malaria kwenye nyanda za juu za Kenya Magharibi: Udhibitaji wa pamoja wa njia nne za kupigana na malaria na uchunguzi wa manufaa kupitia utafiti wa safari moja moja.

**Vituo vinavyohusi:** Wizara ya Afya na Kitengo cha kupigana na Maleria, Kenya, Chuo Kikuu cha Usafi na Utafiti wa Magonjwa ya Kusambazwa ya London,UK (LSHTM), Chuo Kikuu cha Radboud Nijmegan & KEMRI/CDC Kenya.

**Vinara wa Utafiti:** Dkt. Teun Bousema (LSHTM, Chuo Kikuu cha Radboud Nijmegan), Dkt. Jonathan Cox, Dkt. Chris Drakeley (LSHTM)

**Wanaosimamia utafiti Mashinani:**

| Dkt. Jennifer Stevenson | Meneja wa Mradi | 0713-510845 |
| --- | --- | --- |
| Gillian Stresman | Msomi wa madudu /Meneja mashinani | 0713-880159 |
| Amrish Baidjoe | Msomi wa kinga za mwili / Meneja mashinani | 0704-131154 |
| Chrispin Owaga | Mratibu wa Mradi | 0724-273318 |
| Diana Okello | Afisa mkuu wa Mradi | 0720-801542 |
| Elizabeth Marube | Daktari ya magonjwa ya binadamu | 0722-282097 |
| Robin Oriango | Msomi wa Uabara/Mwangalizi (akida) | 0721-823215 |
| Albert Okoth | Msomi wa Uabara/ Mwangalizi (akida) | 0721-824882 |
| Wycliffe Odongo | Meneja wa data | 0724-694426 |

**Lengo:** Kudhibitisha matokeo ya njia za kupigana na maleria zinapotumika katika jamii zilizo na maleria nyingi katika maeneo ya nyanda za juu palipo ueneaji usiotaratibu ya Rachuonyo

**Kielezo na sababu za utafiti**

Juhudi zinazohusu usambazaji wa kiandarua (neti) ya mbu na unyunyiziaji dawa nyumbani zinazofanywa Wizara ya Afya ya Kenya zimeleta manufaa katika kupunguza ueneaji wa maleria. Hata hivyo, maleria bado inazidi kuwa tatizo haswa kwa jamii zilizo mashinani. Utafiti zilizofanywa mwaka wa 2011 katika maeneo yanapata maleria kwa urahisi katika nyanda za juu za Rachuonyo zilionyesha kuwa kuna maeneo kati ya haya yanayosalia na kesi za maleria nyingi kuliko mangine yanayoyazingira.

Ili kupunguza ueneaji wa maleria katika maeneo ya nyanda za juu za Racuonyo tunapanga kuweka juhudi thabiti kwa haya maeneo ‘masugu’. Maeneo masugu ya maleria ni muhimu kwa vile yanaweza kuwa pahali panapohifadhi vidudu vya maleria kati ya misimi ya ueneaji na kwa hivyo kuwa kama pahali pa uhifadhi wa usambazaji wa maleria. Kuelekeza juhudi hizi kwenye maeneo haya yenye uwingi wa maleria wakati ueneaji wa maleria umepungua kunaweza leta nafasi mwafaka ya kupunguza maleria katika jamii nzima. Kwa hivyo tunapanga kufanya utafiti juu ya kutumia njia 4 tofauti za kupigana na maleria; 1) upigaji wa dawa ya maleria inayodumu ndani ya nyumba 2) kupatiana neti ya mbu zinazotibiwa na hudumu 3) kutilia dawa ya kuwaua mbu mahali wanaozaana 4) kupima na kutibu watu wanoishe kwenye nyumba wanazoishi wenye wameambukizwa maleria. Tunapanga kutumia hizi mbinu katika maeneo sugu 8 ndani ya nyanda za juu za Rachuonyo na baadaye kulinganisha uwingi wa maleria katika jamii zilizo ndani na zinazozingira maeneo haya yenye hayatapokea juhudi hizi. Kwa kutumia mbinu hizi kwa pamoja wakati tunapotarajia matokeo makubwa zaidi, tunatarajia kupunguza ueneaji wa maleria ndani ya maeneo sugu na maeneo yaliyo karibu nayo.

**Namna utafiti utafanywa**

Pahali pa uatfiti: Tungependa kufanya huu utafiti katika nyanda za juu za Rachuonyo katika maeneo ya mashinani, yenye urefu wa mita 1400 hadi mita 1600 juu ya maji. Utafiti utafanywa katika maboma yaliyo Rachuonyo Kusini, katika eneo inayotoshea kilomita 5 upande moja kwa kilomita 25 upande mwengine (5km x 25km), linalozingira Ringa (tazama mchoro). Katika eneo hili neti za mbu yamesambazwa kwa kiwango cha 60-70 kwa miya, na juhudi za kupigia nyumba dawa ya mbu zimekuwa tangu 2008.

Kuchagua washiriki wa utafiti: Katika miezi ya Juni-Julai 2011 picha ya setlaiti ilipigwa na eneo hili la kilomita 5 upande moja kwa kilomita 25 upande mwengine likagawanywa kwa vipande vya mita 500 kwa mita 500 (500m x 500m). Kwa kutumia picha hili, ramani zinazoonyesha usambazaji wa maleria katika jamii zilitengenezwa. Hizi ramani zilitumika kutambua jamii zilizokuwa na kesi nyingi za maleria kuliko zingine (yaani maeneo sugu). Kwa haya maeneo sugu, 16 yatachaguliwa na nusu ya haya yataorodheshwa bila mapendeleo kupokea juhudi za kupigana na maleria. Maboma yota yaliyoko kwa maeneo yatakayochaguliwa yatatembelewa na kupatiwa hizi juhudi. Kutafsiri ueneaji wa maleria, watu 200 ndani ya kila eneo sugu na watu 500 wanaoishi kwa maeneo yanayozingira kila eneo sugu watachaguliwa bila mapendeleo kushiriki. Tunatarajia kufanya hivi mara tatu kwa mwaka. Utafiti wa kiwanza utafanyika mwanzoni mwa msimu wa usambazaji wa maleria (Machi), ufuatao umepangwa kufanyika wakati wa msimu wa usambazaji ya juu (Julai), wa mwisho ukifanyika mwishoni mwa msimu wa usambazaji wa maleria (Septemba). Utafiti huu unatarajiwa kuchukuwa miaka 2.

Mpangilio wa mikakati: Kabla ya kudumisha juhudi hizi katika jamii, maboma na watu binafsi watatembelewa kupatiana ruhusa ya kujiunga na utafiti. Mwanzo wa mikakati kamili utakuwa mwezi Machi 2012.

*Kupiga dawa ya mbu inayodumu nymbani:* ile dawa itakayopigwa kwa manyumba itajulikana baada ya mashauriano ya kina na DOMC. Vitendo na upigaji dawa wenyewe utafanywa na wanofanya utafiti; upigagji utafanywa kabla ya katikati ya msimu wa ueneaji wa juu zaidi, wakati unaosadikiwa kuwa mwafaka mno na wadadisi wengi wa ueneaji (19) na baada ya miezi 6. Wanautafiti wataviondoa nje ya nyumba ya kupigiwa dawa vyombo vyote viwezavyo kusongezwa. Vile visivyoweza kuondolewa vitasukumwa katikati ya vyumba na kufunikwa na karatasi ya plastiki. Dawa ita nyunyiziwa na chombo cha kubebwa mgongoni. Kuta na paa za nymba zote bomani (za kuishi na zinginezo) zitanyunyiziwa kwa muda usiopungua dakika 15 kulingana na maelezo kuhusu dawa itakayotumiwa. Nyumba zote zitafungwa kwa muda wa saa 2 halafu milango na madirisha yote yatafunguliwa ili hewa safi iingie kwa muda wa dakika 30. Baada hii, mwenye nyumba ataweza kuingia ili asafishe nyumba. Upigaji dawa utarudiwa kila miezi 6.

*Usambazaji wa neti iliyotibiwa ya kudumu:* Kabla ya kuanza kutilia juhudi hizi, wanaofanya utafiti watazuru maboma ili kuhesabu nambari ya mahali pa malazi kwa kila nyumba. Wakati wa kutia juhudi (Machi 2012), wanautafiti watapeana neti moja kwa kila mahali pa malazi ndani ya nyumba, kuitandaza na kutoa maelezo kamili juu ya matumizi bora na namna ya kuilinda. Ili kuepukana na kuwashwa kutokana na dawa inayotumika kwa neti, neti zote zitatandazwa nje kwa muda usiopungua masaa 24 kabla ya kupeanwa kwa jamii ili nguvu ya dawa ididimike. Baada ya mwezi 1, matumizi bora ya neti itaangaliwa. Kukihitajika, maelezo zaidi juu ya neti itarudiliwa na neti za ziada zipeanwe kukiwa kutakuwa na zile zimepotea au kuraruka. Kutembelewa ya tatu kutafanyika ili kuangalia matumizi ya neti tena baada ya miezi 6 tangu kuanza kwa juhudi, sambamba na raundi ya pili ya kupiga dawa. Kwa hivi sasa, uenyeji upya na hali ya neti itakaguliwa tena na neti mpya zipeanwe kunapohitajika.

*Kupima na kutibu wa binafsi:* Katika maneneo sugu yatakayopokea juhudi hizi, joto la mwili (harara) litapimwa kwa kila mtu kutumia vikipimajoto linalowekwa kwa masikio na sampuli ya damu kuchukuliwa kwa kudunga vidole. Kwa wale walio na umri kati ya miezi 6 had miaka 20, hii damu itatumika kupima maleria kutumia mbinu ya haraka mno (RDT) na kuchunguza ukosefu wa damu, kutayarisha sampuli kwa kioo ili kutumika kutafuta vidudu visababishavyo maleria, na kuhifadhi kiasi kidogo kwa karatasi spesheli. Hizi sampuli zitachunguzwa kwa maabara iliyoko Kisumu kutahmini ueneaji wa maleria. Ikiwa moja au zaidi ya watu walio na umri kati ya miezi 6 had miaka 20 atapatikana na vidudu hivi, basi huenda watu wengine nyumbani humo pia wameambukizwa. Kwa wale wanaozidi umri wa miaka 20, ni wale tu walio na jota la kimwili watakaopimiwa maleria kutumia RDT, hata hivyo mipango yota yanasalia ilivyoelezwa hapo awali. Kiwango cha damu kitakachochukuliwa kitakuwa ni1/10 cha kijiko cha chai. Kukiwa mtu mmoja ataonyeshwa kuambukizwa maleria kupitia RDT, basi kila mmojaaishiye nyumbani humo, isipokuwa wanawake wajaa wazito, atatibiwa na dawa ya Coartem kulingana na ratiba ya kitaifa. Coartem haukubaliki kutumiwa kuwatibu wajaa wazito. Ikiwa mwanamke aliye na umri ya kuweza kuzaa (miaka 13-45)atapatikana na maleria, au kuishi kwa nyumba aliyepatikana mwenye ameambukizwa maleria, mfanyi kazi wa afya ya uma atamtembelea na kutathmini ikiwa nan mimba. Hii itafanywa kupitia maswali machache yaliyoandikwa, na kama itahitajika, mkojo itapimwa. Akipatikana na mimba au mwanamke hakubalii kupimwa ikiwa ana mimba, atapelekwa kwa kituo cha afya ili achunguzwe zaidi na kutibwa vilivyo, kabla ya kurudishwa nyumbani. Gharama zote zitabebwa na huu utafiti. Kama sehemu muhimu ya huu utafiti, kutakuwa na daktari ya magonjwa ya binadamu ili kutoa ushauri juu ya matibabu na madhara yanayoweza kutokana na madawa. Washirika wote pia wataelezewa kiwango chao cha damu mwilini punde tu watakaopimwa, na yeyote atakaye patikana na upungufu, yaani anemia, atatibiwa kulingana na ratiba ya kitaifa.

*Taratibu ya kuchunguza ueneaji wa malerai:* Timu ya wafanyi kazi wa mashinani waliopewa mafunzo maalum watawatafuta wale waliochaguliwa kushiriki kwa uatfiti. Timu hii itawaelezea washiriki (na wazazi wa watoto wasio na miaka 18) kuhusu utafiti huu, na kuomba ruhusa ya kufanya utafiti kwao, pamoja na wazazi au walezi wa atoto watakaotaka kushiriki, na wana umri unaozidi miezi 6. Watoto wasiofikisha umri wa miezi 6 hawataingia kwa uchunguzi huu. Hii ni kwa sababu Coartem yenye itatumika kutibu maleria haikubaliki kutumiwa kwa watoto wa umri mdogo.

Mwenye nyumba au anayechunga nyumba ataulizwa maswali kuhusu

- Vitu vilivyotumika kujenga nyumba
- Vitu muhimu katika mazingira vinavyoweza kutambua boma (kama shule, soko)
- Matumizi ya mbinu za kupambana na maleria (neti, upigaji dawa nyumbani)

Majibu yatarekodiwa kwa vyombo vya PDA. Wanaofanya kazi ya mashinani watapima joto la mwili (harara) wa kila mtu anayeishi kwa kila boma wakitumia vikipimajoto linalowakwa sikioni. Wale watakaokuwa na joto la juu watapimiwa maleria kwa RDT. Watu wote wawe na joto la juu au la watachukuliwa damu kidogo ya kidole. Hii itatumika kuangalia kama kuna damu kidogo mwilini (safura) kutumia chombo cha HemoCue na kuchukuwa pia damu ya pima zinginezo za maleria katika maabara iliyoko Kisumu. Pima hizi zitatafuta chembe (antibodies) cha kupigana na maleria mwilini na DNA ya vidud vya maleria. Hizi sampuli hazitatumika kwa utafiti ya magonjwa mengine.

Matokeo ya RDT na damu (safura) yatakuwa tiyari hapo hapo na yatarekodiwa kwenye PDA. Wenye watapatikana na maleria kupitia RDT watatibiwa na Coartem kulingana na ratiba ya kitaifa. Ilivyoelezwa hapa juu, wanawake wanaoshukiwa kuwa wajaa awzito hawawezi kutibiwa na Coartem na kwa hivyo watapelekwa kwa vituo vya afya kilicho karibu kwa matibabu.

Wenye watapatikana kuwa na safura (anemia) watakabidhiwa dawa ya kurudisha damu au Ferro B. ikiwa washiriki watakuwa na upungufu wa damu mkubwa sana watatumwa kwa kituo cha afya kilicho karibu. Ikiwa washiriki watkuwa wamedhoofika sana kutokana na magonjwa mengine, nao pia watatumwa kwa kituo cha afya. Ikiwa gari la mradi/utafiti litaweza kumfikia mgonjwa kwa haraka, basi watafikishwa kwenye kituo cha afya au hospitali iliyo karibu kulingana na mahitaji.

**Matibabu/Kutumwa hospitalini ya mashinani na manufaa kwa jamii**

- Washiriki wote wataelezwa ikiwa wana joto la juu
- Walio na joto la juu watapimiwa maleria bila malipo na kujua ikiwa na maleria hapo hapo
- Watakaoonyeshwa wana maleria kupitia RDT ni umri wao unazidi miezi 6 na si wajaa wazito watapatiwa Coartem bila malipo
- Yeyote atapatikana na damu kidogo atapatiwa dawa ya kurudisha damu bila malipo
- Yeyote atapatikana amedhoofika atatumwa kwenye kituo cha afya na kupelekwa ikiwezekana
- Majibu yote ya uchunguzi wa ueneaji yatapishwa afisi za DO, DC, DMOH na DPHO, pamoja na chifu na jamii zote baada ya kukamilika kwa utafiti
- Ripoti zote za mradi zitatumwa kwa idara ya DOMC, Nairobi

**Appendix A2: Study Information Sheet for Intervention Communities**

**English**

Reducing the burden of malaria by targeting hotspots of transmission and improving malaria control measures in the highlands of Western Kenya: Simultaneous rollout of four malaria control interventions and evaluation by cross-sectional surveys.

**Organizations involved:** Ministry of Health and Division of Malaria Control, Kenya, London School of Hygiene and Tropical Medicine UK (LSHTM), Radboud University Nijmegan, & KEMRI/CDC Kenya

**PIs:** Dr. Teun Bousema (LSHTM, Radboud University Nijmegan), Dr Jonathan Cox, Dr Chris Drakeley (LSHTM)

**Field Team Supervisors:**

| Dr. Jennifer Stevenson | Project Manager | 0713-510845 |
| --- | --- | --- |
| Gillian Stresman | Epidemiologist/Field Manager | 0713-880159 |
| Amrish Baidjoe | Molecular Immunologist /Field Manager | 0704-131154 |
| Chrispin Owaga | Study Coordinator | 0724-273318 |
| Diana Okello | Administrative Officer | 0720-801542 |
| Elizabeth Marube | Clinical Officer | 0722-282097 |
| Robin Oriango | Lab Tech/ Field Supervisor | 0721-823215 |
| Albert Okoth | Lab Tech/ Field Supervisor | 0721-824882 |
| Wycliffe Odongo | Data Manager | 0724-694426 |

**What is KEMRI/CDC, Radboud University and LSHTM?**

KEMRI/CDC is a government organisation and Radboud University and London School of Hygiene and Tropical Medicine are universities that carry out medical research. Research is different from normal treatment because research aims to find better ways of preventing and treating illness in the future for everybody’s benefit. We are asking your permission for your compound/you/your child to participate in a research study.

What is this research about?

The Kenya Medical Research Institute, the Centers for Disease Control and Prevention, the London School of Hygiene and Tropical Medicine, Radboud University Nijmegen and the Ministry of Health are doing a research study. We want to learn how we can improve malaria control in highland areas of western Kenya by targeting interventions to those areas where malaria is most common.

Your community is located in an area of Rachuonyo that is exposed to intense malaria. It has therefore been selected for intensive malaria control that is given in addition to those malaria interventions already provided by the Kenyan Ministry of Health. As part of this study, we will provide each compound in randomly selected communities with four different malaria control measures:

1. Spraying the inside of all habited and non habited structures with insecticides (IRS)
2. Providing long lasting insecticide treated bednets (LLINs)
3. Applying safe chemicals to water where mosquitoes breed (larviciding)
4. Testing people within a house and treating everyone if at least one person has malaria

In this research we want to find out if by targeting all 4 forms of malaria control in areas with a lot of malaria we can reduce the number of people getting sick from malaria. In our study, communities that have high malaria will be selected by chance into two groups: some communities will be given the malaria control interventions and others will not. The decision on which communities receive the malaria interventions is not decided by the research team. It is decided by a system based on chance. In this instant your community has been randomly selected to receive the interventions and we would like to get your permission to carry out these interventions.

What will it involve for my compound?

Census

Initially we would like to take details of the number of people staying in your house, to map your house, take details of number of nets in your house and how many sleeping spaces there are. This will help us know how many compounds there are in our study areas, and how many people stay within compounds in Rachuonyo. It will help us know where the houses are and how many houses will receive the interventions.

Malaria Control

In the communities that will be offered the malaria interventions, all people who stay in that area will be offered four different control measures.

IRS: This will take place every 6 months in addition to the normal spraying conducted by the Ministry of Health. . The study team will remove all movable items from the buildings that will be sprayed. Any large items that cannot be removed will be moved into the centre of the room and covered with plastic sheets. Sprayers will be identified by badges and protective clothing. They will spray the insecticide using a back pack sprayer. The walls and roof of all buildings in the compound will be sprayed for at least 15 minutes. The buildings will be left locked for 2 hours when no one should enter. After this the doors and windows can be opened and the building allowed to air out for 30 minutes. After this the head of the house may enter to sweep clean.

LLINs: The number of insecticide treated bednets needed for each sleeping space will be checked before distribution. We will provide long lasting insecticide treated nets to every household. We will return four weeks after this initial assessment to make sure nets are being used properly and again 6 months later to ensure that the nets are still in good condition.

Larviciding: The third intervention targeting young mosquitoes is called larviciding. We will locate all bodies of water where we think mosquitoes breed. With the permission of the Ministry of Health and the local chiefs, we will put a safe chemical in the water that is safe for humans but will kill the mosquitoes. After we administer the chemical, humans can still drink the water, use it for washing, or eat food/ fish living in the water.

Testing and treating: We will take temperatures of everyone that stays in the compound over the age of 6 months. We will also collect blood samples and test for low blood for everyone. The blood will be collected using a sterile lancet to make a small prick on a finger. This sample will be less than 1/10 a teaspoon (1/2 ml). For those people aged 6months to 20 years the blood will be used for a rapid diagnostic test for malaria. For those over 20 years of age and who have a fever they too will receive a rapid test for malaria. For everyone the blood will be put in a machine to test for low blood. It will also be used to collect a small amount of blood into a tube and onto filter paper to later double check the tests for malaria and see if you had malaria in the past. These tests will mainly be done in the laboratories in Kisumu, but some tests will be carried out in the Netherlands and the UK to follow international standards for quality control. We would also like to store samples until the study has finished and store samples for 5 years to adhere to international guidelines on Good Clinical Practice for research.

You will know the results of the rapid diagnostic tests for malaria and low blood immediately but any tests carried out in the labs on blood samples will not have your name on it and so results from these will not be given to you. For tests done immediately everyone with low blood will be offered treatment with iron. If anyone in the compound is positive for malaria, EVERYONE who stays there will be offered treatment with Coartem. We are doing this because if one person is positive for malaria it is likely that others who stay in the same area will also be positive and because it is possible that some parasites may be missed by the rapid diagnostic tests. We would also like to ask about what you do to prevent malaria, whether you/your child have had recent fever or other illness, whether you/your child have taken drugs recently, and if you have traveled or lived outside of your village recently.

We will not be able to treat pregnant women in this study at home because Coartem is not recommended for pregnant women. If you are a woman between 13 and 45 years of age, and are found to have malaria or stay in a compound where someone has malaria we will ask you questions about whether or not you might be pregnant. If a woman may be pregnant, but is unsure, we will request that they do a pregnancy test at their home using a urine sample. If you are pregnant you will be taken by the team to a health facility to see a doctor and treated appropriately. The project will cover the costs of this.

Malaria surveys

To assess whether the malaria control has worked we will be testing up to 500 people from a community before and after the control has been implemented for malaria and compared to those areas where no malaria control took place. The compounds that will be visited for the survey will be randomly selected. This means that there is a chance that your compound may not be selected for the survey. If your compound has been selected for the survey we will fully describe the procedures at the time of the survey.

Everyone who tests positive for malaria will receive treatment. If you/your child has low blood, we will provide iron for treatment. As stated above we cannot treat pregnant women with Coartem. Any woman with fever and positive for malaria will be asked to go to the health facility for appropriate treatment.

How many visits will there be?

Altogether we expect to make up to six visits to your compound each year of the study, and the study is expected to last two years. The first visit (now) is to explain the study and to carry out a census of the area. The later visits will be to carry out the survey before we give interventions, then to carry out LLIN distribution, treating positive compounds and carrying out IRS and larviciding. After giving interventions we will carry out 2 more malaria surveys and also check on how LLINs are being used. When we carry out the surveys a separate information sheet will be given. We will also visit compounds that received treatment three days after treatment to assess progress and to check for any side-effects from the anti-malarial treatment or from the insecticides used on the nets and for spraying.

How long will this take?

The indoor residual spraying will take approximately 30 minutes. Checking for bednets, distributing if necessary, and instructions on proper hanging and use will take around 30 minutes. The testing for malaria and anaemia, administration of medications if necessary, and short questionnaire should only take about 45 minutes.

Are there any risks or disadvantages to me/my child of taking part?

Some people have a mild allergic reaction to the insecticide used for spraying and on bednets. We will reduce this risk by asking you not to enter your house for 2 hours after the spray and also to allow the house to air for another 30 minutes before entering. LLINs will be hung for 24 hours before we give them to you to allow the insecticide to weaken enough to reduce reactions in people sleeping under them. We will check to see whether you do have side effects on the three days we give treatment and one week after we sprayed your house.

The drugs used are known to be safe in most people, and are now in common use in Kenya. Artemisinin-based combination therapy (ACTs), such as Coartem, has no known adverse events for children. Iron treatment for anaemia also has no serious adverse effects. If treated, you will be asked questions after you have completed the treatment about any possible side effects of the drugs and will receive appropriate medical care for any such problem during the course of the study. Coartem is not safe for pregnant women so pregnant women who are positive for malaria will be taken to health facilities for appropriate treatment.

The study team will visit you after issuing IRS, LLIN and drugs to monitor any side-effects. On the study team is a clinical officer, Elizabeth Marube. If you experience any side effects you must contact her immediately on 0722282097. If for any reason the clinical officers monitoring the study think you would benefit from leaving this study they will recommend this to you and ensure that you receive the normal standard of treatment for people who are not in the study

The finger prick blood sample may cause minor temporary discomfort. The amount of blood removed will be too small to affect your health. There may be a small bruise or temporary mild pain on the finger where the blood is taken. There is also a small chance of infection when blood is drawn. However, our careful procedures make this very unlikely.

The information generated will also be very useful for making decisions about malaria control in your community and our country as a whole.

Are there any benefits to taking part?

As your compound has been randomly selected for targeted malaria control you will free mosquito control interventions, and a test for fever and anaemia. Those in your compound that are between 6 months and 20 years of age will also benefit from getting a test for malaria. If a malaria test is positive, free treatment will be provided to everyone in the compound. The knowledge gained from this study will help the country of Kenya in determining the best way to target malaria control measures.

**What will happen if I don’t agree to participate?**

All participation in research is voluntary. You are free to decide if you want to take part or not. You have the right to refuse. If anyone does not want to go on with this study they can stop at any time. Participants will still receive the recommended standard of care if they do not take part. If you do agree you can change your mind at any time and withdraw from the research. This will not affect any care now or in the future. If you choose to withdraw from the study, you have the right to ask us not to use any of the previously collected data. If this happens, we will accommodate this request and remove your information and samples from our systems.

What happens to the samples?

All of the tests that are needed as part of this research will be done locally in Kisumu, Kenya or for double checking in the Netherlands and the UK. Re-testing of some samples outside Kenya is done to follow international standards for quality control.

**Who will have access to information about participants in this research?**

Information about study participants will be kept confidential to the maximum extent allowable by law. All our research records are stored securely in locked cabinets and on password protected computers. Only staff researchers will have access to the data. Your name and house number will be stored with the data but will not appear on any reports. Your blood sample will be given a unique code. Your name will not appear on your blood sample but can be linked to the data with your name. At the end of the study, we will remove your name and the house number from the data so that no one can identify your information or your blood sample.

Who has allowed this research to take place?

This research has been approved by international independent expert committees in UK, the Netherlands and KEMRI SSC/ERC in Nairobi and a committee in Kisumu to make sure the research is conducted properly and that participants’ safety and rights are respected.

This research is supported by the KEMRI/CDC, Radbound University Nijmegan, and the London School of Hygiene and Tropical Medicine who will pay for any treatment or compensation in the unlikely event of any injury resulting from this study.

**What do I do if I have questions?**

You may ask any of our staff questions at any time. Their contacts are given at the beginning of this information sheet. You can also contact those who are responsible for the care of your child and this research:

Dr. Jennifer Stevenson, at KEMRI/CDC –P.O. Box 1578 - 40100, Kisumu, Kenya

Tel: +254 713 510 845 *or*

Elizabeth Marube Clinical Officer… at KEMRI/CDC –P.O. Box 1578 - 40100, Kisumu, Kenya

Tel: +254 722282097

If you have questions about your rights as a study participant, if you have concerns about the research, feel you have been harmed in any way, or if you want to ask someone independent anything about this research please contact

Community liaison officer, George Ben Okoth at KEMRI/CDC – P.O. Box 1578 - 40100, Kisumu, Kenya Tel: +254 725956512

*or*

Secretary, KEMRI/ERC, P. O. BOX 54840-00200, Nairobi, Telephone: 020 272 2541

If you are happy to be part of this study and to receive the interventions described above we would like you to sign some consent forms at each visit we make. There will be 4 forms; the first is to allow us to take a census of your house; one is for consent from the head of house to allow IRS and LLIN distribution to occur in your house; the second is for consent from each household member to allow us to carry out the testing and treating intervention; the fourth is consent for us to be able to carry out the malaria surveys.

**Mamoko A2: Oboke Mar Tiend Wach Nonro mar Ogendni ma Nonro Itimoe**

**Dholuo**

Duoko pek mar maleria piny ka idimbo dago ma landruok tuo ng’enyie kod jiwo yore mag mag gayo malerie e hosi mag gode man milambo mar Kenya: Riwo yore ang’wen mag gayo maleria kod nonro margi ma dichiel.

**Migepe ma nitie:** Migao mochung’ ne gayo maleria, Kenya, Kar Tiegruok ma London mar Ler kod Tuoche malandore UK (LSTMH), Mbalariany mar Radboud Nijimegan, kod KEMRI/CDC Kenya.

**Jotend nonro:** Lkt. Teun Bousema (LSHTM, Radboud University Nijmegan), Lkt. Jonathan Cox, Lkt. Chris Drakeley (LSHTM)

**Jotend tich mar pap:**

| Lkt. Jennifer Stevenson | Jatelo maduong’ | 0713-510845 |
| --- | --- | --- |
| Gillian Stresman | Jalony mar kute mafuyo/Jarit pap | 0713-880159 |
| Amrish Baidjoe | Jalony mar kaka del gayo tuoche/Jarit pap | 0704-131154 |
| Chrispin Owaga | Ogaye mar nonro | 0724-273318 |
| Diana Okello | Jachung’ mar tayo ofis nonro | 0720-801542 |
| Elizabeth Marube | Laktar mar touche dhano | 0722-282097 |
| Robin Oriango | Jalony e tije lab/ Nyapara mar pap | 0721-823215 |
| Albert Okoth | Jalony e tije lab/ Nyapara mar pap | 0721-824882 |
| Wycliffe Odongo | Jalony mar data (weche mawuok e nonro) | 0724-694426 |

**KERI/CDC, Radboud University kod LSHTM gin ang’o?**

KEMRI/CDC en bad sirikal kendo Radboud kod LSTMH gin mbalariany ma bende timo nonro mag ngima dhano. Nonro opogore gi thieth ma pile nikech nonro temo dwaro yore mabeyo mag geng’o kod thiedho tuoche e kinde ma bang’e ne ji duto. Wakwayo rusa mari/dalani/nyathini mondo odonji e nonro.

Nonroni en mar ang’o?

KEMRI, CDC, Mbalariany mar LSHTM, Malariany mar Radboud, kod Migao mar Ngima Dhano timo nonro. Wadwaro ng’eyo kaka wanyalo jiwo gayo maleria kuonde mag gode man Kenya ma milambo ka wadimbo gi yore mag gayo maleria e kuonde ma maleria ogyoe dago.

Ogandau nitie ei Rachuonyo kama malaria ng’enyie. Ma omiyo oyiere ne keto okange mapek mag gayo maleria ma ichiwo ka imedo ewi mago ma Migao mar Ngima Dhano ma Kenya oseketo. Kaka mano mabiro timore e nonroni, wabiro miyo dala ka dala ma nitie e gwenge mokwany radharadha yore ang’wen mag gayo maleria:

1. Goyo yadh suna ei udi duto ma ji odakie kod ma ok odagie (IRS)
2. Chiwo nede ma oketie yadh suna madak (LLINs)
3. Kiro yedhe ma ok richo gi ngima dhano e pige ma suna nyuolore (larviciding)
4. Pimo ji kendo thiedho ji duto ma nitie e ot tek ni ng’ato achiel oyudi gi malaria

E nonroni wadwaro ng’iyo kapo ni keto okange 4 duto mag gayo maleria ei gwenge ma nitie maleria mang’eny nyalo duoko piny kwan ji mayudo tuo mar maleria. E nonroni, ogendni man gi maleria mang’eny ibiro keti radharadha e kidienje ariyo: gwenge moko ibiroketie okange mag gayo maleria ka moko to ok bi timnegi kama. Gi sani, ogandani oseyier mondo oyud yore mag gayo maleria kendo wakwayo thuolo mari mondo waket okangegi.

En ang’o ma biro timore e dalana?

Kwano ji

Ka wachako, wabiro dwaro kawo kwan mar ji ma odak e odi, kawo map mar odi, kawo kwan mar need ma nitie ei odi kod kwan mar kuonde nindo. Ma biro konyowa ng’eyo kwan mar delni ma nitie e gwenge ma watimoe nonro, kod kwan ji ma odak e delni man Rachuonyo. Obiro bende konyowa ng’eyo kuonde ma ute nitie kod kwan mar ute mabiro yudo okange mag gayo maleria.

Gayo maleria

E gwenge mabiro yudo yore mag gayo maleria, ji duto ma odak e gweng’no biro yudo okange 4 mag gayo maleria.

Goyo yath ei udi: Mae biro timore bang’ fweche auchiel auchiel ka omedore ewi goyo yath mapile ma Migao mochung’ ne Ngima Dhano timo. Jogo matayo nonro biro golo oko gik manyalo sudo oko mar ot ma igoyoe yath. Gigo duto madongo ma ok nyal gol oko ibiro choki e chuny die rot kendo ibiroum kod jwala. Jok magoyo yath biro fwenyore gi baj ma kende, kod rwakruok mageng’o hinyruok. Gibiro goyo yath ka gitiyo kod rago yath ma iting’o e tok. Tat udi kod kor udi duto ma nitie e dala ibiro goye yath kuom thuolo ma ok tin ne dakika 15. Udigi biro dong’ ka olor makare kuom seche 2 bang’e dhoudi kod dirisni ibiro yaw kendo we thuolo kuom dakika 30. Bang’ ma wuon ot biro bedo thuolo donjo yweyo ot.

Nede suna ma oketie yath ma dak: Kwan nede suna m nitie yath ma dwarore e kuonde nindo duto ibiro kaw kapok opog nede. Wabiro pogo nedegi e udi duto. Wabiro duogo bang’ jumbe ang’wen bang’ kweno mokwogoni mondo wane ka nede itiyogo makare, kendo bang’ dweche 6 mondo wane ni nede pod beyo.

Kiro yath e pige ma suna nyuolore: Ma en okang’ mar adek ma idimbogo suna matindo ma iluongo ni larviciding. Wabiro manyo pige duto ma waparo ni suna tokoe. Ka wakawo thuolo mar Migao mochung’ ne Ngima Dhano to kod ruodhi mag gwenge, wabiro keto yath moro maonge rach kuom ngima dhano e pi mondo oneg suna. Ka waseketo yadhni, dhano pod biro nyalo modho pini, luoko kode, kata chamo chiemo/rech modak e pi.

Pim kod thieth: Wabiro pimo liet mar dend ji duto ma odak ei dala ma hikgi ohingo dweche 6. Wabiro bende kawo remo matin mar rango tin mar remo. Remoni ibiro gol gi gik maler mar chwoyo wi lith lwedo. Remoni bende biro bedo matin ne 1/10 mar ojiko chae. Remb jogo ma hikgi ni e kind dweche 6 kod higni 20 ibiro ti godoe fweny maleria mapiyo. Jogo ma hikgi ohingo 20 ma bende dendgi liet mokalo bende ibiropimnegi maleria. Ne ji duto, ibiro ti kod remo e pimo tin mar remo e del. Bende ibiro ti kod fururu moro matin kuom keto remo matin e kalatas moro ma kende ma ibiro ti godo e nono ka adieri ng’ato nigi maleria, kendo ka ng’ato osebedo gi maleria e kinde ma osekalo. Pimgi ng’enyne biro tiomre e lab ma Kisumo, to moko iboirotim Loka Netherlands gi UK niketch wadwa luwo ratiro ma oket mar piny ngima.

Wadwaro bende kano remo nyaka nonro rum.Kendo wakan remo kuom higni abich ka luwo ratiro mar piny ngima e weche ma yore mabeyo mathieth gi nonro.

Ibiro ng’eyo dwoko mar pim mapiyo mar maleria kod remo matin, kendo dwoko duto mawuok e lab ok bi bedo gi gi nyingi kendo ok bimiyigi. Kuom pim ma dwokogi wuok mapiyo ma nyiso remo matin ibirochiw thieth maduoko remo. Ka ng’ato e dala nigi maleria, JI DUTO modak kanyo ibirothiedhi gi Coartem. Watimo ma nikech ka ng’ato achiel nigi maleria to yot mondo jomoko modak kanyo bende nitie gi maleria, kendo nikech seche moko pim mapiyo nyalo koso fwenyo maleria. Wadwaro bende penjo gik ma itimo mondo igeng’ maleria, ka in/nyathini osebedo gi del machwakre kata tuoche moko machiegni, ka in/nyathini osemuonyo yath machiegni, kendo ka iselimo kata dak oko mar gweng’u machiegni.

Ok wabi thiedho mine man gi ich e nonroni e dala nikech Coartem ok opuodhi tiyogo kuom mon ma yach. Ka in dhako ma hike nitie kind higni 13-45, to oyudi gi maleria kata idak e dala ma ng’ato nitie gi maleria to wabiro penji ka dibed kod ich. Ka miyo nigi ich to ok en gi adier, wabiro kwayo mondo gipimre e dala ka gitiyo gi lach. Ka iyach to jotaa noro biro teri e od thieth mondo inee laktar kendo iyud thieth makare. Nonro biro chung’ne garamagi.

**Nono ng’eny mar maleria**

Mondo wane ka bende yoregi gayo maleria malong’o wabiro pimo ji 500 e oganda kapod ok oketi to kod bang’ tiyo okangegi mag gayo maleria, kendo pimo magi gi ogendni ma yore ok otiyego kata. Delni ma ibiro timgo nonro ibiro kwany radharadha. Kapo ni dalani okwany mar donjo e nonro to wabiro miyi ler makare kaluwore gi nonro, e kinde ma obiro chakore.

Ji duto ma oyudi gi maleria biro yudo thieth. Ka in/nyathini nitie gi remo matin, wabiro chiwo yath mamedo remo. Kaka osler malo kae, ok wabithiedho mine mapek gi Coartem. Miyo ma dend chwakore kendo nitie gi maleria ibirokwa mondo odhi e od thieth mondo oyud thieth ma kende.

Limbe adi ma ibiro tim?

Duto wageno timo limbe auchiel e dalani higa ka higa, kendo nonro igeno ni biro kawo higni ariyo. Limbe mokwongo (sani) en mar mar lero tiend nonro kendo kawo kwan ji e gwenge. Limbe mabiro luwo ma gin mag pimo ogendni kapok waketo okange mag gayo maleria, bang’e to wabiro pogo nede man gi yath madak, thiedhi ji e delni, kod goto yath ei udi ka bende wakiro yath e pige ma nitie e aluora. Bang’ keto okangegi wabiro pimo maleri safar 2 ka bende warango kaka nede itiyogo. E seche ma watimo magi, oboke machielo malero tiend nonro ibiro chiw. Wabiro bende limo delni ma oyudo thieth ndalo adek bang’thieth mondo wane kaka jidhi to kapo ni nitie rach moro amora ma thieth dikel, kata ka oa kuom yath ma okti e nede.

Mae biro kawo thuolo marom nadi?

Goyo yath ei udi biro kawa kaka dakika 30. Rango nede, kendo chiwo moko ka dwarore, kod golo ler ewi moyo kod tiyo gi net eyo maber biro kawo gimoro dakika 30. Pimo maleria gi tin mar remo, thieth kaka dwarore, kod duoko penjo matin duto onego kaw mana dakika 45.

Bende nitie hinyruok kata rach ma an/nyathina nyalo yudo e kinde nonro?

Ji moko dendgi nyalo ilo matin nikech yath ma igoyo ei udi kata mothiedhgo nede. Wabiro duoko ma chien ka wakwayi ni kik idonji e odi nyaka seche 2 rum bang’ goyo yath kendo weyo yamo aokwadh ot kuom dakika 30 kapok idonjo. Nede methiedhi gi yath madak ibiro moo oko kuom seche 24 kapok wamiyigi mondo oduok teko yath piny ma ok choch dend joma nindo ebwogi. Kinde ka kinde wabiro rango kapo ni ineno chochni moro amora.Wabiro ng’iyo ka ibedo gi chandruok mora mora e ndalo adek ma wachiwo yath. to kendo bang juma achiel ka wakwoyo yath e kor ot.

Yien mitiyogo duto osepuodhi onge rach e ngima dhano, kendo tiyo alanda ei Kenya sani. Yien molos gi artemisinin, kaka Coartem, onge rach moro amora malich kuom nyithindo. Yadh medo remo bende onge rach moro malich. Ka othiedhi, ibiro miyi penjo moko ka isetieko thieth ewi chochni moro amora madi ine nikech yien mothiedhiogo, kendo ibiro yudo kony mar thieth e kinde mar nonro. Coartem ok opuodhi tiyogo e mine man gi ich koro mine mayach to nitie gi maleria ibiro or e ute thieth ne theith makare

Jota nonro biro limi bang’ goyo udi yath, pogo nede kod yien mondo girang ka rach moro nitie. Kuom jogi nitie laktar mar dend dhano, Elizabeth Marube. Ka ineno chochni moro amora to nyaka itudori kode e simbe, 0722282097. Kapo ni lakteche mag dend dhano marango kaka nonroni dhi oneno ni inyalo konyori ka iweyo bedo ja nonroni to gibiro leroni ma kendo neon ni iyudo thieth mapile kaka imiyo jomoko ma ok nitie e nonro.

Remo matin migolo ewi lith lwedo nyalo kelo ilo moro matin ahinya. Remo migolo to tin ma ok nyal chocho ngimani. Kama ogolie remo nyalo gwarore matin nono, kata iwinjie rem moro matin ahinya. Seche moko kute moko nyaloga donjo kama ogolie remo. Kata kamano, yore ma watiyogo ler ahinya kendo ma ok yot mondo otimre.

Weche mowuok bende biro konyo ahionya e ng’ado rieko ewi gayo maleria e ogandau kod pinywa Kenya duto.

**Bende niti yuto kuom bedo e nonro?**

Kaka dalani osekwany radharadha mar ketoe okange mag gayo maleria, ibiro yudo yore mag agyo suna, kod pim mag chwakruok del gi remo matin maonge chudo. Ji ma titie e dalani ma hikgi en dweche 6 nyaka higa 20 bende biro konyore kod pim mar maleria. Ka pim oyudo ni nitie maleria nenga’to ang’ata to ibirothiedh ji duto ma nitie edalani. Weche mowuok e nonroni biro konyo piny Kenya bidho yo maber mogik mar ketao okange mag gayo maleria.

**En ang’o mabiro timore ka ok ayie donjo e nonro?**

Bedo e nonro duto en yiero mari. In thuolo yiero ka idonjo kata ok idonji e nonro. Teko duto en mari yiero donjo kata tamori. Ka ng’ato ok dwa dhi nyime gi nonroni to ginyalo chung’ kinde moro amora. Jo modonjo e onro pod biro yudo thieth makare kata ka giweyo dhi nyime gi nonro. Ka iyie to bende pod inyalo loko pachi saa asaya ma iwuogi e nonro. Ma ok bi chocho thieth moro amora ma iyudo sani kata e kinde mabiro. Ka iyiero mondo iwuoge e nonro,in gi ratiro mar tamowa tiyo gi weche mane osechok e kinde mokalo.kotimore kamano wabiro luo wachni no, kendo wabogolo wach moramora mamuli kendo kata remo mane wasekawo.

**Remo ibirotim nadi?**

Pim duto mag nonroni ibirotim mana Kisumo ka, ei Kenya, mak mana matin mar rnago ka pimgi dhi maber ema ibirotim loka Netherlands gi UK. Pim mar ariyo ma itimo loka, en niketch wadwa luwo ratiro ma oket mar piny ngima.

**Ji mage mabirobedo gi thuolo mar neno weche ewi jok mantie e nonroni?**

Weche ewi jok mantie e nonroni ibirokan mopondo mogik kaka chik oyie. Weche duto ibirokan kuonde ma ogo kiful kod komputa molor e ma ok fwenyre. Mana jota nonro ema biro bedo thuolo chopo kuode mokanie wechegi. Nyingi kod namba odi ibirokan gi weche ma e wiyi to ok biwuok e ripot moro amora. Rembi ibiromii namba moro ma kende. Nyingi ok biwuok e rembi to inyalotude kod weche mantie gi nyingi. Bang’ giko nonro, wabiro golo nyingi gi namba odi e data mondo kik ng’ato nyal dimbo weche ma e wiyi kata mag rembi.

En ng’ano ma opuodho nonroni mondo otim?

Nonroni opuodhi gi duonde ma kende ma oriwo jolony mapinje mang’eny man UK, Netherlands kod mano ma Nairobi, to kod duol man Kisumo, mondo omi nonro otim eyo makare ka irango ni ngima kod ratiro mar jo mantie e nonro omi luor.

Nonroni ojol gi KEMRI SSC/ERC, Mbalriany mar Radboud Nijmegan, kod Mbalariany mar London mar Ler Aluora gi Tuoche Dhano Malandore (LSHTM), mabiro chulo thieth duto, kata golo chudo kapo ni hinruok moro owuok nikech nonroni.

**Ka an gi penjo to atimo ang’o?**

Inyalo penjo jataa norno moro amora penjo saa asaya. Yore mar tudruok kodgi nitie ewi obokeni. Inyalo bende tudori kod jogo mochung’ ne rito nyathini e nonroni:

Lkt. Jennifer Stevenson, man KEMRI/CDC –P.O. Box 1578 - 40100, Kisumu, Kenya

Sime: +254 713 510 845

*Kata*

Elizabeth Marube Laktar mar dend dhano… man KEMRI/CDC –P.O. Box 1578 - 40100, Kisumu, Kenya, Sime: +254 722282097

Ka in kod penjo kuom ratiro magi kaka ng’at modonjo e nonro, ka in kod wach ewi nonroni, ka iparo ni isehinyori e yo moro amora, kata ka idwaro penjo ng’ato ma ok otudore gi nonroni penjo ewiye to yie itudori kod

Jambetre mar tudruok e kor gwenge, George Ben Okoth man KEMRI/CDC – P.O. Box 1578 - 40100, Kisumu, Kenya Tel: +254 725956512

*Kata*

Jagoro, kamitii mar ratiro mar nonnro (KEMRI/ERC), P. O. BOX 54840-00200, Nairobi, Sime: 020 272 2541

Ka imordonjo e nonroni kendo yudo okange mag gayo maleria ma oler malo kae, wabiro kwayi mondo iket koki e oboke moko e seche duto ma wabirotimo limbe. Biro bedo oboke 4; mokwongo biro miyowa thuolo kawo kwan ji e odi; mar ariyo en mar wuon ot mondo ochiw thuolo mondo ogo yath ei ot kendo opog nede e odi; mar adek en mar ji duto ma nitie e ot mondo oyie opimgi kendo othiedhgi; mar ang’wen en mar miyowa thuolo mondo wanon ng’eny mar maleria.

**Nyongeza A2: Maelezo juu ya utafiti kwa Jamii Wahusika**

**Kiswahili**

Kupunguza uzito wa ugonjwa wa malaria kwa kuelekeza juhudi mahali palipo ueneaji wa juu na kuimarisha juhudi za kupigana na malaria kwenye nyanda za juu za Kenya Magharibi: Udhibitaji wa pamoja wa njia nne za kupigana na malaria na uchunguzi wa manufaa kupitia utafiti wa safari moja moja.

**Vituo vinavyohusi:** Wizara ya Afya na Kitengo cha kupigana na Maleria, Kenya, Chuo Kikuu cha Usafi na Utafiti wa Magonjwa ya Kusambazwa ya London,UK (LSHTM), Chuo Kikuu cha Radboud Nijmegan & KEMRI/CDC Kenya.

**Vinara wa Utafiti:** Dkt. Teun Bousema (LSHTM, Chuo Kikuu cha Radboud Nijmegan), Dkt. Jonathan Cox, Dkt. Chris Drakeley (LSHTM)

**Wanaosimamia utafiti Mashinani**

| Dkt. Jennifer Stevenson | Meneja wa Mradi | 0713-510845 |
| --- | --- | --- |
| Gillian Stresman | Msomi wa madudu /Meneja mashinani | 0713-880159 |
| Amrish Baidjoe | Msomi wa kinga za mwili / Meneja mashinani | 0704-131154 |
| Chrispin Owaga | Mratibu wa Mradi | 0724-273318 |
| Diana Okello | Afisa mkuu wa Mradi | 0720-801542 |
| Elizabeth Marube | Daktari ya magonjwa ya binadamu | 0722-282097 |
| Robin Oriango | Msomi wa Uabara/Mwangalizi (akida) | 0721-823215 |
| Albert Okoth | Msomi wa Uabara/ Mwangalizi (akida) | 0721-824882 |
| Wycliffe Odongo | Meneja wa data | 0724-694426 |

**KEMRI/CDC, Chuo Kikuu cha Radboud na LSHTM ni nini?**

KEMRI/CDC ni idara ya serikali na Chuo Kikuu cha Radboud pamoja na Chuo Kikuu cha Usafi na Utafiti wa Magonjwa ya Kusambazwa ya London ni vyuo vinavyoendesha utafiti wa magonjwa ya binadamu. Utafiti ni tofauti na matibabu ya kawaida kwa sababu utafiti hutafuta njia mwafaka za kuzuia na kutibu magonjwa kwa siku za usoni kwa manufaa ya kila mtu. Tunaomba ruhusa yako ili boma lako/wewe/mtoto wako ashiriki kwa utafiti.

Utafiti huu ni ya nini?

KEMRI, CDC, LSHTM, Chuo Kikuuu cha Radboud Nijmegan, na Wizara ya Afya wanafanya utafiti. Tungependa kujua vile tunaweza kuimarisha vita dhidi ya maleria katika maeneo ya nyanda za juu ya magharibi ya Kenya kwa kutilia maanani maeneo yenye maleria nyingi zaidi.

Jamii yako inaishi katika eneo la Rachuonyo lenye hupata malaria nyingi sana. Kwa hivyo imechaguliwa kutiliwa juhudi thabiti dhidi ya malaria, zenye zitaungana nguvu na juhudi zilizomo za Wizara ya Afya ya Kenya. Kama sehemu ya huu utafiti, tutapeleka juhudi nne tofauti kwa kila boma katika maeneo yaliyochaguliwa bila mapendeleo:

1. Kunyunyuzia dawa ya madudu ndani ya kila nyumba ya kuishi na nyinginezo
2. Kupatiana neti iliyo na dawa ya kudumu
3. Kutia madawa yasiyokuwa na madhara kwa binadamu kwa maji ili kuwaua mbu
4. Kupima watu waishio kwa nyumba moja na kuwatibu wote ikiwa mmoja ana maleria

Katika huu utafiti tunataka kujua kama kwa kutumia hizi mikakati 4 dhidi ya maleria kwa maeneo yaliyo na maleria nyingi tunaweza kupunguza idadi ya wanaogonjeka kutokana na maleria. Kwa huu uchunguzi, jamii zilizo na maleria nyingi zitachaguliwa na kuwekwa kwa vikundi viwili bila mapendeleo: jamii zingine zitapokea hizi judi dhidi ya maleria na zingine hazitazipata. Kauli dhidi ya jamii zenye zinapokea hizi juhudi haikatwi na wanaoendesha utafiti. Inafanywa na njia taratibu inayotumia bahati. Kwa sasa jamii yako ishachaguliwa bila mapendeleo na tungeomba ruhusa yako ili tuzitie hizi juhudi.

Boma langu litahusika kivipi?

Kuhesabu watu

Mwanzoni tungependa kuchukuwa takrimu juu ya idadi ya watu wanaoishi kwa nyumba yako, kuchukuwa ramani ya nyumba yako, na idadi ya neti na mahali pa malazi ziliko nyumbani kwako.hii itatusaidia kujua ni maboma mangapi yaliyomo katika maeneo ya utafiti, na ni watu wangapi wanaoishi kwa maboma yaliyomo Rachuonyo. Itatusaidia kujua panapo nyumba na ni nyumba ngapi zitapokea hizi juhudi.

Juhudi dhidi ya maleria

Katika jamii zitakazopokea juhudi dhidi ya maleria, watu wote wanaoishi katika eneo hilo watazipokea juhudi zote nne.

Kunyunyizia nyumba dawa ya kudumu: Hii itafanyika baada ya kila miezi 6 ikiwa ni nyongeza juu ya ile inayofanywa na Wizara ya Afya. Timu la utafiti watatoa nje ya nyumba zenye zitanyunyiziwa dawa vitu vyote watakaoweza. Vitu vikubwa vyenye haviwezi kutolewa vitasukumwa katikati ya vyumba na kufunikwa na karatasi ya plastiki. Wanyunyizaji watatambuliwa kwa baji na nguo zao za kujikinga. Watanyunyiza dawa wakitumia chombo cha kubabe mgongoni. Kuta na paa za nyumba zote bomani zitanyunyiziwa dawa kwa muda usiopungua dakika 15. Nyumba zote zitafungwa kwa muda wa saa 2, wakati hakuna atakayeruhusiwa kuingia. Baadaye milango na madirisha yote yatafunguliwa ili hewa safi iingie kwa muda wa dakika 30. Baada hii, mwenye nyumba ataweza kuingia ili asafishe nyumba.

Neti zilizotibiwa zenye zinadumu: Idadi ya neti zilizotibiwa zenye zitahitajika kwa kila pahali pa malazi itakaguliwa kabla ya usambazaji wa neti. Tutapatiana neti zilizotibiwa na dawa ya kudumu kwa kila nyumba. Tutarudi baada ya wiki nne kufuatia huu ukaguzi wa mwanzo ili kuhakikisha kwamba neti zinatumika vizuri, na tena baada ya miezi 6 kuhakikisha hizi neti bado ziko kwa hali nzuri.

Kutia dawa kwenye maji yenye mbu huzaana: Mkakati wa tatu unanuiya kumenyana na mbu wachanga. Tutatmabua mahali pote pa maji penya tunafikiri mbu hutaga na kuzaana. Tukiwa na ruhusa ya Wizara ya Afya na machifu wa eneo, tutatia majini kemikali isiyokuwa na madhara kwa binadamu lakini inaua mbu.baada ya kuweke hii kemikali, watu wataweza kunywa haya maji, kuyafulia, ama kula chakula/samaki wanaishi kwa hayo maji.

Kupima na kutibu watu: Tutapima joto la mwili (harara) la kila mtu anayeishi kwa boma na ana umri unaozidi miezi 6. Tutachukuwa damu na kupima upungufu wa damu (safura) kwa kila mtu. Damu itatolewa kutumia wembe safi kabisa kusababisha tundu ndogo kwa kidole. Hii sampuli itapungua 1/10 ya kijiko cha chai. Kwa wale walio na umri kati ya miezi6 na miaka 20, damu itatumika kupima maleria kupitiambinu za upesi (RDT). Kwa wale waliozidisha umri wa miaka 20 na wanao joto (harara) la juu, hao pia watapimiwa maleria na RDT. Kwa kila mtu damu itawekwa kwa chombo maalum ili kupima upungufu wa damu (safura). Itatumika pia kuhifadhi sehemu kidogo kwa karatasi maalum kupitia mrija ili kudhibitisha ubora wa uchunguzi unaofanywa na RDT, na kuangalia pia kama umewahi kuambukizwa maleria. Hizi uchunguzi zitafanywa san asana hapa Kisumu, ingawaje chahe zitafanywa Uhulanzi na Uingereza Kulingana viwango vya kimataifa kwa ajili ya kudhibiti ubora. Tungependa pia kuhifadhi sampuli mpaka utafiti utakapokamilika. Na kuhifadhi sampuli kwa muda wa miaka tano kuzingatia miongozo juu ya njia bora ya hospitali kwa ajili ya utafiti

Utayajua matekeo ya uchunguzi wa maleria ya RDT na ile ya upungufu wa damu hapo hapo, lakini uchunguzi zote zitakazofanywa kwa maabara hazitakuwa na jina lako na kwa hivyo hautapewa majibu. Kwa uchunguzi zitafanywa upesi, yeyote atakayepatikana na upungufu wa damu atapewa madawa ya kurudisha damu. Ikiwa mtu mmoja atapatikana na maleria kwa boma, KILA MTU anayeishi kwa hilo boma atatibiwa na Coartem. Tunafanya hivi kwa sababu ikiwa mtu mmoja ameabukizwa maleria, kuna uwezekano wa juu kuwa wengine wanaoishi katika eneo hilo pia wameambukizwa kwa vile huenda vidudu vingine vikaepuka kutambulika na RDT. Tungependa pia kuuliza kuhusu chenye unafanya ili kuzuia maleria, ikiwa wewe/mtoto wako amekuwa na harara (joto jingi) hivi majuzi, au ugonjwa wowote hivi majuzi, na ikiwa wewe/mtoto wako amemeza madawa hivi majuzi, na kama umesafiri au kuishi nje ya kijiji chako hivi majuzi.

Hatutaweza kuwatibu wanawake wajaa wazito kwa huu utafiti nyumbani kwa sababu Coartem haipendelewi kutumika kwa wanawake wajaa wazito. Ukiwa mwanamke aliye na umri wa miaka 13- miaka 45, na umepatikana na maleria, au unaishi kwa nyumba aliyepatikana mwenye ameambukizwa maleria, tutakuuliza maswali kuhusu uwezekano wa kuwa na mimba. Ikiwa mwanamke anaweza kuwa mjaa mzito na hana uhakika, tutauliza ili wafanye uchunguzi wa kuwa na mimba nyumbani kwao wakitumia mikojo yao.ukiwa mjaa mzito utapelekwa nah ii timu hadi kwa kituo cha afya kumwona daktari na kutibiwa vilivyo. Gharama zote zitabebwa na huu utafiti.

Uchunguzi wa ueneaji wa maleria

Ili kutathmini ikiwa juhudi dhidi ya malaria zimeleta manufaa tutawapima watu 500 kutoka eneo moja kabla na baada ya kutekelezwa kwa juhudi hizi, na kulinganisha na maeneo mengine ambayo hayakuteleza hizi juhudi. Maboma yatakayotembelewa yamechaguliwa bila mapendeleo. Hii inamaanisha ya kwamba kuna uwezekano kuwa boma lako linawezawachwa nje ya yenye yatatembelewa. Boma lako likichaguliwa, tutakueleze mengi kuhusu huu utafiti wakati ukiwadia.

Yeyote atakayepatikana na maleria atatibiwa. Ikiwa wewe/mtoto wako ana upungufu wa damu, tutakupatia dawa ya kurudisha damu. Ilivyoezwa hapo mbeleni, hatutaweza kuwatibu wanawake wajaa wazito na Coartem. Mwanamke yeyote atakayekuwa na joto jingi (harara) na kudhibitishwa kuwa na maleria ataambiwa aende kwa kituo cha afya ili kupata matibabu yafaayo

Tutatembelewa mara ngapi?

Kwa jumla tunatarajia kulitembelea boma lako mara sita kila mwaka, na utafiti unakusudiwa kuendelea kwa miaka miwili. Ziara la kwanza (sasa hivi) ni ya kuwaelezea kuhusu utafiti, na kuchukuwa idadi ya watu katika maeneo ya utafiti. Maziara yatakayofuata yatakuwa ya kufanya ukaguzi kabla ya kutekeleza mikakati dhidi ya maleria, halafu kupatiana viandarua (neti), kuwatibu walioambukizwa na kunyunyizia nyumba na maji dawa ya kuwua mbu. Baada ya kutekeleza mikakati hii tutafanya ukaguzi ya maleria safari 2 na pia kuangalia vile neti zinatumika. Wakati tukifanya ukaguzi hizi, tutapatiana maelzo mengi mpya tena. Tutayazuru maboma yaliyopata matibabu siku tatu baada ya matibabu ili kutathmini vile wanaendelea na kuangaliwa kukiwa namadhara yoyote kutokana na dawa ya maleria ama dawa yeny imewekwa kwa net.

Hii itachukuwa muda gani?

Kunyunyizia nyumba dawa itachukuwa takriban dakika 30. Kukagua neti nakupeana zingine kukihitajika, na maelezo kuhusu kutandazwa kamili na matumizi mazuri ya neti itachukuwa kama dakika 30. Kupima maleria na upungufu wa damu, kupatiana dawa kukihitajika, na kujibu maswali machache itachukuwa takriban dakika 45.

Kunayo hatari ama madhara yoyote kwangu/kwa mtoto wangu tukishiriki?

Watu wengine wanaweza washwa kidogo kutokana na dawa inayonyunyiziwa nyumbani ama ile iliyoko kwa neti. Tutapunguza hatari hii kwa kukuuliza usiingie kwa nyumba yako kabala ya muda wa masaa 2 baada ya kunyunyiziwa dawa, na pia kuwacha nyumba ipate hewa safi kwa muda wa dakika 30 kabla ya kuingia. Neti zitatandazwa kwa muda wa masaa 24 kabla ya kukukabidhiwa ili nguvu ya dawa ididimike na kupunguza madhara kwa wenye wanalala ndani zao. Tutaangalia kila mara ikiwa unapta madhara yoyote. Tuta chunguza iwapo kutatokea madahara yoyote kwa siku tatu tunapo toa matibabu na pia wiki moja baada ya kunyunyiza dawa ukutani.

Dawa zinazotumika zinajulikana kuwa salama kwa watu wengi, na zinatumika pakubwa Kenya. Dawa zinazotoka kwa artemisinin (ACTS), kama Coartem, hayana madhara dhahiri kwa watoto. Dawa za kurudisha damu pia hazina madhara dhahiri. Ukitibiwa, utaulizwa maswali baada ya kumaliza kumeza dawa kuhusu kuweko kwa madhara yoyote, na utapewa matibabu halisi kulingana na shida iliyoko wakati utafiti ukiendelea. Coartem sis lama kwa wanawake wajaa wazito kwa hivyo wanawake wajaa wazito walio na maleria watapelekwa kwa vituo vya afya kwa matibabu yafaayo.

Timu ya utafiti watakutembelea baada ya kunyunyizia nyumba, kupeana neti na dawa ili kukadiria madhara. Kwa hii timu ni daktari ya magonjwa ya binadamu, Elizabeth Marube. Ukiona madhara yoyote ni lazima uwasiliane naye haraka iwezekanavyo kwa 0722282097. Ikiwa kwa sababu yoyote madaktari wataonelea utapata manufaa kwa kujiondoa kwa utafiti watakuelezea hii na kuhakikisha unapokea matibabu kamili kama ya yeyote asiyekuwa kwa utafiti.

Sampuli ya damu kutoka kwa kidole kinaweza kusababisha takalifu kidogo ya muda mfupi. Kiasi cha damu kinachochukuliwa ni kidogo sana na hakiwezi kudhuru afya yako. Kunawezatokea mkwaruzo kidogo na maumivu madogo yanayodumu muda mfupi sana pahali panapotolewa damu. Kuna uwezo mdogo pia ya kuambukizwa damu ikitolewa. Lakini, njia mwafaka tunazotumia inapunguza haya hatari kabisa.

Matokeo yatakayopatikana yatakuwa muhimu sana kwa kukata shauri kulingana na kupiga vita dhidi ya maleria kwa jamii yako na Kenya kwa jumla.

Kunayo manufaa ya kushiriki?

Boma lako limechaguliwa bila mapendeleo kupokea mikakati dhidi mya maleria kwa hivyo utapata kujikinga na mbu, kupimiwa joto la mwili na upungufu wa damu bila malipo. Wote walioko bomani mwako na wanao umri wa miezi6 hadi miaka 20 pia watanufaika na kupimiwa maleria. Ikiwa mtu mmoja atapatikana na maleria, kila mtu atakayekuwa kwa boma atapewa matibabu bila malipo. Maarifa yatakayopatikana kwa utafiti huu yatasaidia nchi ya Kenya kutambua mikakati mwafaka zaidi ya kupambana na maleria.

**Nini itafanyika ikiwa sitakubali kushiriki?**

Kushiriki kwa utafiti ni kwa hiari. Una uhuru wa kuamua ikiwa utajiunga au la. Una haki ya kukataa. Ikiwa mtu hataki kuendela kuwa kwa utafiti huu anaweza kujiondo wakati wowote. Washiriki bado watapokea huduma kama kawaida hata kama hawatashiriki. Ukikubali unaweza kubadilisha maamuzi yaki wakati wowote na kujiondoa kwenya utafiti. Hii haitadhuru huduma utakazopata kwa sasa au siku zijaazo. Ikiwa utachagua kujiondoa katika utafiti huu, una haki ya kutuuliza kutotumia taarifa yoyote ya awali inayokuhusu. hilo likitokea tutalisikiliza ombi hili na kuondoa taarifa yako na sampuli kutoka kwa mfumo wetu

Nini itafanyiwa sampuli?

Uchunguzi zote zenye zinafanywa kama sehemu ya huu utafiti zitafanywa hapa Kisumu, Kenya, au kuhakikisha ubora wa uchunguzi huko Uholanzi na Uingereza. Marudio ya upimaji wa sampuli nje ya kenya yanafanyika kulingana na viwango vya kimataifa kwa ajili ya kudhibiti ubora

**Nani atakuwa na uwezo wa kuona taarifa kuhusu washiriki wa utafiti huu?**

Taarifa kuhusu washiriki wa uatfiti itahifadhiwa kwa siri ya hali ya juu kulingana na sheria. Rekodi zetu zote za utafiti zinawekwa kwa makabati yanayofungwa kikamilifu na kufuli na kwa tarakilishi iliyowekewa vifunguo vya siri. Ni watafiti tu watakaokuwa na ruhusa ya kuona taarifa za utafiti. Jina lako na namba ya nyumba yako itakuwa na taarifa hizi lakini haitakuwa kwa ripoti yoyote. Sampuli ya damu yako itabandikiwa nambari fulani ya kisiri. Wakati utafiti utakapokamilika, tutaondoa jina lako na nambari ya nyumba yako kwa taarifa zako ili hakuna atakayetambua taarifa zako wala sampuli yako ya damu.

Ni nani aliyeidhinisha huu utafiti ufanywe?

Utafuti huu umeidhinishwa na mabaraza huru ya kimataifa ya Uingereza, Uholanzi, na mabaraza huru ya kitaifa yaliyo Nairobi, na kamati iliyo Kisumu ili kuhakikisha utafiti unafanywa kwa ustadi na usalama na haki za washiriki ziheshimiwe.

Utafiti huu unagharamiwa na KEMRI SSC/ERC, Chuo Kikuu cha Radboud Nijmegan, na Chuo Kikuu cha Usafi na Utafiti wa Magonjwa ya Kusambazwa ya London (LSHTM), ambao watalipia matibabu na kutoa fidia ikiwa kwa bahati mbaya madhara yatatokea.

**Nikiwa na swali ninafanyaje?**

Unaweza kumwuliza yeyote kati ya wanaoendesha utafiti maswali wakati wowote. Namna za kawasiliana nao imepeanwa mwanzoni wa maelezo haya. Unaweza pia kuwasiliana na wale wanaohusika na huduma kwa mtoto wako kwa huu utafiti:

Dkt. Jennifer Stevenson, akiwa KEMRI/CDC-S.L.P. 1578- 40100, Kisumu, Kenya

Simu: +254 713 510 845, *au*

Elizabeth Marube (daktari ya magonjwa ya binadamu) akiwa KEMRI/CDC - S.L.P. 1578-40100, Kisumu, Kenya.

Simu: +254 722282097

Ukiwa na maswali kuhusu haki zako kama nyumba mshiriki, tashwishi kuhusu utafiti wenywe, au ukiwa unataka kuuliza mtu asiyeegemea utafiti huu kabisa kuhusu huu utafiti, wasiliana na:

Afisa wa utangamano na jamii, George Ben Okoth wa KEMRI/CDC – S.L.P. 1578-40100, Kisumu, Kenya. Simu: +254 725956512

*Au*

Katibu, Kamitii la Kitaifa ya Haki na Usawa wa Uendeshaji wa Utafiti (KEMRI/ERC) - S.L.P. 54840-00200, Nairobi. Simu: 020 272 2541

Ukiridhishwa na ungependa kuwa kwa utafiti huu na kupokea mikakati dhidi ya maleria iliyoelezwa hapa juu tungepend utie kidole fomu ya ruhusa kila tutakapokutembelea. Kutakuwa na fomu 4; ya kwanza itatuwezesha kupata idadi ya watu wa nyumba yako; ya pili itakuwa ya kuomba ruhusa ya kiongozi ya nyumba ili nyumba zinyunyiziwe dawa na neti kupeanwa kwa nyumba; ya tatu ni ya kuomba ruhusa ya kila anayeishe kwa nyumba kushiriki kwa kupimwa na kupatiwa dawa; ya nne ni ya kuomba ruhusa ili tuweze kukagua ueneaji ya maleria.

**Appendix A3: Study Information Sheet for malaria surveys in all clusters**

**English**

Reducing the burden of malaria by targeting hotspots of transmission and improving malaria control measures in the highlands of Western Kenya: Simultaneous rollout of four malaria control interventions and evaluation by cross-sectional surveys.

**Organizations involved:** Ministry of Health and Division of Malaria Control, Kenya, London School of Hygiene and Tropical Medicine UK (LSHTM), Radboud University Nijmegan, & KEMRI/CDC Kenya

**PIs:** Dr. Teun Bousema (LSHTM, Radboud University Nijmegan), Dr Jonathan Cox, Dr Chris Drakeley (LSHTM)

**Field Team Supervisors:**

| Dr. Jennifer Stevenson | Project Manager | 0713-510845 |
| --- | --- | --- |
| Gillian Stresman | Epidemiologist/Field Manager | 0713-880159 |
| Amrish Baidjoe | Molecular Immunologist /Field Manager | 0704-131154 |
| Chrispin Owaga | Study Coordinator | 0724-273318 |
| Diana Okello | Administrative Officer | 0720-801542 |
| Elizabeth Marube | Clinical Officer | 0722-282097 |
| Robin Oriango | Lab Tech/ Field Supervisor | 0721-823215 |
| Albert Okoth | Lab Tech/ Field Supervisor | 0721-824882 |
| Wycliffe Odongo | Data Manager | 0724-694426 |

**What is KEMRI/CDC, Radboud University and LSHTM?**

KEMRI/CDC is a government organisation and Radboud University and London School of Hygiene and Tropical Medicine are universities that carry out medical research. Research is different from normal treatment because research aims to find better ways of preventing and treating illness in the future for everybody’s benefit. We are asking your permission for you/your child to participate in a research study.

What is this research about?

The Kenya Medical Research Institute, the Centers for Disease Control and Prevention, the London School of Hygiene and Tropical Medicine, Radboud University Nijmegen and the Ministry of Health are doing a research study. We want to learn how we can improve malaria control in highland areas of western Kenya by targeting interventions to those areas where malaria is most common.

Your community is located in an area of Rachuonyo that is exposed to intense malaria. It has therefore been selected to gather information on the location of houses, the number of people who live here, any anti-malaria methods you use and for testing for fever and low blood.

In this research we want to find out if your community has more or less malaria than other communities which have previously been found to have high malaria and which may or may not be receiving malaria control measures. In our study, communities that have high malaria have been selected by chance into two groups: some communities received the malaria control interventions and others did not. The decision on which communities received the malaria interventions was decided using a system based on chance.

What will it involve for my compound?

We will be testing up to 500 people for malaria from a community before and after the control has been implemented and compared to those areas where no malaria control took place. The compounds that will be visited for the survey will be randomly selected. Eeveryone who is above 6 months of age in each randomly selected compound will be asked if they want to take part in the survey. We will map your house, ask you questions about your house and what you do to prevent malaria. We will ask whether you or your child has had a recent fever, measure your temperature, and collect blood samples. If you or your child have a temperature, we will also test you/your child for malaria using a rapid diagnostic test. We collect the blood by pricking the finger or heel of you/your child and taking approximately 1/10 teaspoon (less than ½ ml) of blood. The amount of blood we will take is very small. We will check for low blood. We will take this small blood sample into a small tube and also put some blood on a filter paper so that we can later check whether you have been infected with malaria in the past and whether the malaria you have is difficult to treat. These tests will be carried out mainly in laboratories in Kisumu, Kenya but results may be confirmed on some samples in the Netherlands and the UK. Your samples may also be stored for the duration of the study. International research codes on Good Clinical Practice prescribe that we keep samples for a minimum of 5 years to make monitoring possible. We will ask you if you are happy for us to do so.

Everyone who tests positive for malaria will receive treatment. If you/your child has low blood, we will provide iron for treatment. We will not be able to treat pregnant women in this study because Coartem is not recommended for pregnant women. If you are a woman between 13 and 45 years of age, and are found to have malaria or we will ask you questions about whether or not you might be pregnant. If a woman may be pregnant, but is unsure, we will request that they do a pregnancy test at their home using a urine sample. If you are pregnant you will be referred to a health facility to see a doctor and treated appropriately..

**The procedure will take about 45 minutes.**

**Potential benefits**

You and your child will get a clinical examination and a test for low blood right now. If malaria is suspected, we will also test you or your child for malaria. Treatment is provided free of charge.

**Potential risks**

There may be a small bruise or temporary mild pain on the finger where the blood is taken. There is also a small chance of infection when blood is drawn. However, our careful procedures make this very unlikely.

**Privacy and confidentiality**

Information about you/your child will be kept confidential to the maximum extent allowable by law. The data we collect will be stored on computers at the KEMRI laboratory in Kisian and at the London School of Hygiene and Tropical Medicine. Only staff researchers will have access to the data. Your name and house number will be stored with the data but will not appear on any reports. Your blood filter paper sample will be given a unique code. Your name will not appear on your filter paper sample but can be linked to the data with your name. At the end of the study, we will remove your name and the house number from the data so that no one can identify your information or your filter paper sample.

**Your rights to participate, say no, or withdraw**

You are free to choose to be part of this study. You have the right to refuse. If anyone does not want to go on with this study they can stop at any time. If you do not want to participate in this study, your child can still be checked and treated for malaria or lack of blood at a hospital or health facility.

**Costs and compensation for being in the study**

There are no costs to participate in this study nor will you be compensated for your time.

**Who will have access to information about participants in this research?**

Information about study participants will be kept confidential to the maximum extent allowable by law. All our research records are stored securely in locked cabinets and on password protected computers. Only staff researchers will have access to the data. Your name and house number will be stored with the data but will not appear on any reports. Your blood sample will be given a unique code. Your name will not appear on your blood sample but can be linked to the data with your name. At the end of the study, we will remove your name and the house number from the data so that no one can identify your information or your blood sample.

Who has allowed this research to take place?

This research has been approved by international independent expert committees in UK, the Netherlands and KEMRI SSC/ERC in Nairobi and a committee in Kisumu to make sure the research is conducted properly and that participants’ safety and rights are respected.

This research is supported by the KEMRI/CDC, Radbound University Nijmegan, and the London School of Hygiene and Tropical Medicine who will pay for any treatment or compensation in the unlikely event of any injury resulting from this study.

**What do I do if I have questions?**

You may ask any of our staff questions at any time. Their contacts are given at the beginning of this information sheet. You can also contact those who are responsible for the care of your child and this research:

Dr. Jennifer Stevenson, at KEMRI/CDC –P.O. Box 1578 - 40100, Kisumu, Kenya, Tel: +254 713 510 845 *or*

Elizabeth Marube Clinical Officer… at KEMRI/CDC –P.O. Box 1578 - 40100, Kisumu, Kenya, Tel: +254 722282097

If you have questions about your rights as a study participant, if you have concerns about the research, feel you have been harmed in any way, or if you want to ask someone independent anything about this research please contact

Community liaison officer, George Ben Okoth at KEMRI/CDC – P.O. Box 1578 - 40100, Kisumu, Kenya Tel: +254 725956512 *Or*

Secretary, KEMRI/ERC, P. O. BOX 54840-00200, Nairobi, Telephone: 020 272 2541

If you agree to take part in the survey we will ask you to sign the following consent forms for you and/or your child.

**Mamoko A3: Oboke Mar Tiend Wach Nonro mar Ogendni duto**

**Dholuo**

Duoko pek mar maleria piny ka idimbo dago ma landruok tuo ng’enyie kod jiwo yore mag mag gayo malerie e hosi mag gode man milambo mar Kenya: Riwo yore ang’wen mag gayo maleria kod nonro margi ma dichiel.

**Migepe ma nitie:** Migao mochung’ ne gayo maleria, Kenya, Kar Tiegruok ma London mar Ler kod Tuoche malandore UK (LSTMH), Mbalariany mar Radboud Nijimegan, kod KEMRI/CDC Kenya.

**Jotend nonro:** Lkt. Teun Bousema (LSHTM, Radboud University Nijmegan), Lkt. Jonathan Cox, Lkt. Chris Drakeley (LSHTM)

**Jotend tich mar pap:**

| Lkt. Jennifer Stevenson | Jatelo maduong’ | 0713-510845 |
| --- | --- | --- |
| Gillian Stresman | Jalony mar kute mafuyo/Jarit pap | 0713-880159 |
| Amrish Baidjoe | Jalony mar kaka del gayo tuoche/Jarit pap | 0704-131154 |
| Chrispin Owaga | Ogaye mar nonro | 0724-273318 |
| Diana Okello | Jachung’ mar tayo ofis nonro | 0720-801542 |
| Elizabeth Marube | Laktar mar touche dhano | 0722-282097 |
| Robin Oriango | Jalony e tije lab/ Nyapara mar pap | 0721-823215 |
| Albert Okoth | Jalony e tije lab/ Nyapara mar pap | 0721-824882 |
| Wycliffe Odongo | Jalony mar data (weche mawuok e nonro) | 0724-694426 |

**KEMRI/CDC, Radboud University kod LSHTM gin ang’o?**

KEMRI/CDC en bad sirikal kendo Radboud kod LSTMH gin mbalariany ma bende timo nonro mag ngima dhano. Nonro opogore gi thieth ma pile nikech nonro temo dwaro yore mabeyo mag geng’o kod thiedho tuoche e kinde ma bang’e ne ji duto. Wakwayo rusa mari/dalani/nyathini mondo odonji e nonro.

Nonroni en mar ang’o?

KEMRI, CDC, Mbalariany mar LSHTM, Malariany mar Radboud, kod Migao mar Ngima Dhano timo nonro. Wadwaro ng’eyo kaka wanyalo jiwo gayo maleria kuonde mag gode man Kenya ma milambo ka wadimbo gi yore mag gayo maleria e kuonde ma maleria ogyoe dago.

Ogandau nitie ei Rachuonyo kama malaria ng’enyie. Ma omiyo oyiere mondo mi onwang’ ler ewi kuonde ma ute nitie, kwan ji ma odakie, yo moro amora mar gayo maleria ma itiyogo, kod pimo liet mar del kod tin mar remo.

E nonroni wadwaro ng’eyo ka ogandau nitie gi maleria mang’eny kata matin ne mago mane oseyudi ni nitie maleria mang’eny, ma bende biro kata ok bi yudo okange mag gayo maleria. E nonrowani, ogendni ma nitiegi maleria mang’eny osekwany radhradha mi oketi e kidienje ariyo: mane oyudo okange magayo maleria gi mago ma ok ne oyudo okangegi. Ng’ado ni oganda mage mayudo yore mag gayo maleriagi ne ochopie ka itiyo gi rieko moro mayiero radharadha kaluwo hawi.

Ang’o ma ibirotim e dalana?

Wabiro pimo ji 500 e oganda kapod ok oketi to kod bang’ tiyo okangegi mag gayo maleria, kendo pimo magi gi ogendni ma yore ok otiyego kata. Delni ma ibiro timgo nonro ibiro kwany radharadha. Ji duto ma lhikgi ohingo dweche 6 ibiro penji ka gidwaro donjo e nonro. Wabiro kawo map mar odi, penji ewi weche odi kod yore ma itiyogo e geng’o maleria. Wabiro penji ka in kata nyathini osebedo gi liet mar del machiegni, wabiro pimo liet mar dendi, kendo kawo remo. Ka in kata nyathi oyud gi liet mar del man malo, wabiro pimoni/nyathini maleria ka watiyo gi yo mapiyo ahinya. Wabiro golo remo ewi lith lwedo kat ombong’ tielo mari/nyathini ka wakawo remo matin madirom 1/10 mar ojiko mar chae. Remo ma wakawoni tin ahinya. Wabiro pimo tin mar remo. Wabiro kawo remoni e fururu moro matin ahinya kendo keto moko e kalatas moro ma kende mondo omi wabi wapim ka isebedoga gi maleria kapok nonro ochako, kata ka maleria ma in god tek thiedho. Pimgi ng’enyne biro timore ei lab man Kisumo, Kenya, en mana ni duoko inyalo cheki e moko matin kuomgi e pinje ma loke Netherlands kod UK.Ratiro mar piny ngima ewi thieth kod nonro chiko ni rembi okan nyaka higni abich rum mondo olu nonro maber. Wabiro penji ka imor timo kamano.

Ji duto ma oyud gi maleria biro yudo thieth. Ka in/nyathini nigi remo matin to ubiro yudo yadh medo remo. Ok wabi thiedho mine man gi ich e nonroni e dala nikech Coartem ok opuodhi tiyogo kuom mon ma yach. Ka in dhako ma hike nitie kind higni 13-45, to oyudi gi maleria kata idak e dala ma ng’ato nitie gi maleria to wabiro penji ka dibed kod ich. Ka miyo nigi ich to ok en gi adier,wabiro kwayo mondo ipimri e dala ka gitiyo gi lach. Ka iyach to jotaa noro biro ori e od thieth mondo ine laktar kendo iyud thieth makare.

**Mae biro kawo gimoro dakika 45**

**Yuto manyalo bedo**

In kod nyathini biro yudo pim mar ngima del kod pim mar remo matin e sechegi. Ka wachich ni maleria nitie, wabiro bende pimoni/nyathini maleria. Thieth ichiwo nono maonge chudo.

**Hinyruok manyalo bedo**

Kama ogolie remo nyalo gwarore matin nono, kata iwinjie rem moro matin ahinya. Seche moko kute moko nyaloga donjo kama ogolie remo. Kata kamano, yore ma watiyogo ler ahinya kendo ma ok yot mondo otimre.

**Kano weche mopondo kod luor mar jononro**

Information about you/your child will be kept confidential to the maximum extent allowable by law. The data we collect will be stored on computers at the KEMRI laboratory in Kisian and at the London School of Hygiene and Tropical Medicine. Only staff researchers will have access to the data. Your name and house number will be stored with the data but will not appear on any reports. Your blood filter paper sample will be given a unique code. Your name will not appear on your filter paper sample but can be linked to the data with your name. At the end of the study, we will remove your name and the house number from the data so that no one can identify your information or your filter paper sample.

Weche man e wiyi/nyathini ibirokan mopondo mogik kaka chik oyie. Wechegi duto ibirokan e komputa ei lab mar KEMRI man Kisian, kod Mbalariany mar LSHTM. Mana jota nonro ema biro bedo thuolo chopo kuode mokanie wechegi. Nyingi kod namba odi ibirokan gi weche ma e wiyi to ok biwuok e ripot moro amora. Rembi mantie otas ibiromii namba moro ma kende. Nyingi ok biwuok e otas moting’o rembi to inyalo tude kod weche mantie gi nyingi. Bang’ giko nonro, wabiro golo nyingi gi namba odi e data mondo kik ng’ato nyal dimbo weche ma e wiyi kata otas moting’o rembi.

**Ratiro mari e donjo, tamruok, kata wuok e nonro**

In thuolo yiero ka idonjo kata ok idonji e nonro. In kod ratiro mari mar tamri donjo e nonro. Ka ng’ato ok dwa dhi nyime gi nonroni to ginyalo chung’ kinde moro amora. Ka ok idwa donjo e nonroni, nyathini pod inyalopim kendo thiedhne maleria kata tin mar remo e od thieth.

**Garama kod chudo mar bedo e nonroni**

Onge chudo ma ichiwo mar bedo e nonroni, kendo onge mich ma ibiromiyi ne bedo e nonro

**Ji mage mabirobedo gi thuolo mar neno weche ewi jok mantie e nonroni?**

Weche ewi jok mantie e nonroni ibirokan mopondo mogik kaka chik oyie. Weche duto ibirokan kuonde ma ogo kiful kod e komputa molor e ma ok fwenyre. Mana jota nonro ema biro bedo thuolo chopo kuode mokanie wechegi. Nyingi kod namba odi ibirokan gi weche ma e wiyi to ok biwuok e ripot moro amora. Rembi ibiromii namba moro ma kende. Nyingi ok biwuok e rembi to inyalotude kod weche mantie gi nyingi. Bang’ giko nonro, wabiro golo nyingi gi namba odi e data mondo kik ng’ato nyal dimbo weche ma e wiyi kata mag rembi.

En ng’ano ma opuodho nonroni mondo otim?

Nonroni opuodhi gi duonde ma kende ma oriwo jolony mapinje mang’eny man UK, Netherlands kod mano ma Kairobi, to kod duol man Kisumo, mondo omi nonro otim eyo makare ka irango ni ngima kod ratiro mar jo mantie e nonro omi luor.

Nonroni ojol KEMRI SSC/ERC, Mbalriany mar Radboud Nijmegan, kod Mbalariany mar London mar Ler Aluora gi Tuoche Dhano Malandore (LSHTM), mabiro chulo thieth duto, kata golo chudo kapo ni hinruok moro owuok nikech nonroni.

**Ka an gi penjo to atimo ang’o?**

Inyalo penjo jataa norno moro amora penjo saa asaya. Yore mar tudruok kodgi nitie ewi obokeni. Inyalo bende tudori kod jogo mochung’ ne rito nyathini e nonroni:

Lkt. Jennifer Stevenson, man KEMRI/CDC –P.O. Box 1578 - 40100, Kisumu, Kenya

Sime: +254 713 510 845

*Kata*

Elizabeth Marube Laktar mar dend dhano… man KEMRI/CDC –P.O. Box 1578 - 40100, Kisumu, Kenya, Sime: +254 722282097

Ka in kod penjo kuom ratiro magi kaka ng’at modonjo e nonro, ka in kod wach ewi nonroni, ka iparo ni isehinyori e yo moro amora, kata ka idwaro penjo ng’ato ma ok otudore gi nonroni penjo ewiye to yie itudori kod

Jambetre mar tudruok e kor gwenge, George Ben Okoth man KEMRI/CDC – P.O. Box 1578 - 40100, Kisumu, Kenya Tel: +254 725956512

*Kata*

Jagoro, kamitii mar ratiro mar nonnro (KEMRI/ERC), P. O. BOX 54840-00200, Nairobi, Sime: 020 272 2541

Ka iyie donjo e nonroni to wabiro kwayi mondo iket koki e obokegi ne in kod nyathini.

**Nyongeza A3: Maelezo juu ya utafiti kwa ukaguaji ya malaria kwa pande zote**

**Kiswahili**

Kupunguza uzito wa ugonjwa wa malaria kwa kuelekeza juhudi mahali palipo ueneaji wa juu na kuimarisha juhudi za kupigana na malaria kwenye nyanda za juu za Kenya Magharibi: Udhibitaji wa pamoja wa njia nne za kupigana na malaria na uchunguzi wa manufaa kupitia utafiti wa safari moja moja.

**Vituo vinavyohusi:** Wizara ya Afya na Kitengo cha kupigana na Maleria, Kenya, Chuo Kikuu cha Usafi na Utafiti wa Magonjwa ya Kusambazwa ya London,UK (LSHTM), Chuo Kikuu cha Radboud Nijmegan & KEMRI/CDC Kenya.

**Vinara wa Utafiti:** Dkt. Teun Bousema (LSHTM, Chuo Kikuu cha Radboud Nijmegan), Dkt. Jonathan Cox, Dkt. Chris Drakeley (LSHTM)

**Wanaosimamia utafiti Mashinani**

| Dkt. Jennifer Stevenson | Meneja wa Mradi | 0713-510845 |
| --- | --- | --- |
| Gillian Stresman | Msomi wa madudu /Meneja mashinani | 0713-880159 |
| Amrish Baidjoe | Msomi wa kinga za mwili / Meneja mashinani | 0704-131154 |
| Chrispin Owaga | Mratibu wa Mradi | 0724-273318 |
| Diana Okello | Afisa mkuu wa Mradi | 0720-801542 |
| Elizabeth Marube | Daktari ya magonjwa ya binadamu | 0722-282097 |
| Robin Oriango | Msomi wa Uabara/Mwangalizi (akida) | 0721-823215 |
| Albert Okoth | Msomi wa Uabara/ Mwangalizi (akida) | 0721-824882 |
| Wycliffe Odongo | Meneja wa data | 0724-694426 |

**KEMRI/CDC, Chuo Kikuu cha Radboud na LSHTM ni nini?**

KEMRI/CDC ni idara ya serikali na Chuo Kikuu cha Radboud pamoja na Chuo Kikuu cha Usafi na Utafiti wa Magonjwa ya Kusambazwa ya London ni vyuo vinavyoendesha utafiti wa magonjwa ya binadamu. Utafiti ni tofauti na matibabu ya kawaida kwa sababu utafiti hutafuta njia mwafaka za kuzuia na kutibu magonjwa kwa siku za usoni kwa manufaa ya kila mtu. Tunaomba ruhusa yako ili boma lako/wewe/mtoto wako ashiriki kwa utafiti.

Utafiti huu ni ya nini?

KEMRI, CDC, LSHTM, Chuo Kikuuu cha Radboud Nijmegan, na Wizara ya Afya wanafanya utafiti. Tungependa kujua vile tunaweza kuimarisha vita dhidi ya maleria katika maeneo ya nyanda za juu ya magharibi ya Kenya kwa kutilia maanani maeneo yenye maleria nyingi zaidi.

Jamii yako inaishi katika eneo la Rachuonyo lenye hupata malaria nyingi sana. Imechaguliwa ili kukusanya taarifa kuhusu mahali palipo nyumba, idadi ya watu wanaoishi humu, mikakati yoyote unayotumia dhidi ya malaria, na kupima joto la mwili na upungufu wa damu.

Kwa huu utafiti tungependa kudhibitisha ikiwa jamii yako ina kesi nyingi au kidogo za maleria ikilinganishwa na jamii zingine ambazo zimeshaonyeshwa kuwa na kesi nyingi za maleria, na ambazo huenda zitapokea ama hazitapokea mikakati dhidi ya maleria. Kwa utafiti wetu, jamii zilizo nz kesi nyingi za maleria zimeshagawanywa kwa vikundi viwili: jamii zingine zilipokea mikakati dhidi ya maleria na zingine hazikupokea mikakati. Uamuzi ya kuzikabidhi hii mikakati ulifanywa na taratibu fulani inayotumia bahati.

Nini itafanyika kwa boma langu?

Tutawapima maleria kwa watu 500 kutoka jamii kabla na baada ya utekelezaji wa mikakati na kulinganisha na maeneo ambayo hayajapokea hii mikakati. Maboma yatakayotembelewa yatachaguliwa bila mapendeleo. Kila mtu aliye na umri unaozidi miezi 6 kwa kila boma litakalochaguliwa ataulizwa kama watataka kushiriki kwa utafiti. Tutachukuwa ramani ya nyumba yako, kukuuliza maswali kuhusu nyumba yako na mbinu unayotumia kuzuia maleria. Tutakuuliza ikiwa wewe/mtoto wako amekuwa na harara (joto jingi) hivi majuzi, kuwapima joto la mwili, na kuchukuwa sampuli ya damu. Ikiwa wewe au mtoto wako atapatikana na joto jingi, tutakupimia/mtoto wako maleria kwa RDT. Tunachukuwa damu kwa kuweka tundu ndogo kwa kidole chako/cha mtoto wako na kuto kama 1/10 cha kijiko cha chai ya damu. Hii ni damu kidogo sana. Tutachunguza upungufu wa damu. Tutahifadhi kiasi kidogo yah ii damu kwa karatasi spesheli ili kutathmini baadfaye ikiwa umewahiambukizwa maleria na ikiwa maleria uliyonayo ni sugu kutibu. Hizi uchunguzi zitafanywa sana sana kwa maabara iliyo Kisumu, Kenya, lakini machache yanaweza kupelekwa Uholanzi na Uingereza ili kuhakikisha ubora wa uchunguzi hizi. Sampuli yako huenda itahifadhiwa kwa muda wote wa utafiti. Tutakuuliza kama utakubali tufanye hivyo. codi za kimataifa za mazoezi bora za kliniki zaagiza kuweka sampuli kwa muda wa miaka mitano ya kufanya ufuatiliaji iwezekanavyo

Kila mtu atakayepatikana kuwa na malaria atatibiwa. Ukiwa/mtoto wako atapatikana na upungufu wa damu, tutapatiana dawa ya kurudisha damu. Hatutaweza kuwatibu wanawake wajaa wazito kwa huu utafiti kwa sababu Coartem haipendelewi kutumika kwa wanawake wajaa wazito. Ukiwa mwanamke aliye na umri wa miaka 13- miaka 45, na umepatikana na maleria, au unaishi kwa nyumba aliyepatikana mwenye ameambukizwa maleria, tutakuuliza maswali kuhusu uwezekano wa kuwa na mimba. Ikiwa mwanamke anaweza kuwa mjaa mzito na hana uhakika, tutauliza ili wafanye uchunguzi wa kuwa na mimba nyumbani kwao wakitumia mikojo yao.ukiwa mjaa mzito utapelekwa nah ii timu hadi kwa kituo cha afya kumwona daktari na kutibiwa vilivyo.

**Taratibu hii itachukuwa karibu dakika 45**

**Manufaa yanayoweza kupatikana**

You and your child will get a clinical examination and a test for low blood right now. If malaria is suspected, we will also test you or your child for malaria. Treatment is provided free of charge.

Wewe na mtoto wako mtakaguliwa kiafya na kupimiwa upungufu wa dama sasa hivi. Tukishuku una malaria, pia tutakupima au mtoto wako mkiwa na malaria. Matibabu yanapeanwa bila malipo.

**Hatari yanayoweza kutokea**

Kunawezatokea mkwaruzo kidogo na maumivu madogo yanayodumu muda mfupi sana pahali panapotolewa damu. Kuna uwezo mdogo pia ya kuambukizwa damu ikitolewa. Lakini, njia mwafaka tunazotumia inapunguza haya hatari kabisa.

**Siri na heshima kwa washiriki**

Taarifa kuhusu wewe/mtoto wako itahifadhiwa kwa siri ya hali ya juu kulingana na sheria. Taarifa zote tunazokusanya zinawekwa kwa tarakilishi iliyoko kwa maabara ya KEMRI, Kisian, na Chuo Kikuu cha Usafi na Utafiti wa Magonjwa ya Kusambazwa ya London. Ni watafiti tu watakaokuwa na ruhusa ya kuona taarifa za utafiti. Jina lako na namba ya nyumba yako itakuwa na taarifa hizi lakini haitakuwa kwa ripoti yoyote. Sampuli ya damu yako itabandikiwa nambari fulani ya kisiri. Wakati utafiti utakapokamilika, tutaondoa jina lako na nambari ya nyumba yako kwa taarifa zako ili hakuna atakayetambua taarifa zako wala sampuli yako ya damu.

**Haki yako ya kushiriki, kukataa, au kujiondoa**

Una uhuru wa kuamua ikiwa utajiunga na utafiti huu. Una haki ya kukataa. Ikiwa mtu hataki kuendela kuwa kwa utafiti huu anaweza kujiondo wakati wowote. Ukiwa hautaki kushiriki kwa huu uchunguzi, mtoto wako bado atachunguzwa na kutibiwa maleria au upungufu wa damu kwa hospitali au kituo cha afya.

**Gharama na fidia ya kushiriki**

Hakuna gharama ya kushiriki wala hautalipwa fidia kwa kushiriki.

**Nani atakuwa na uwezo wa kuona taarifa kuhusu washiri wa huu utafiti?**

Taarifa kuhusu washiriki wa uatfiti itahifadhiwa kwa siri ya hali ya juu kulingana na sheria. Rekodi zetu zote za utafiti zinawekwa kwa makabati yanayofungwa kikamilifu na kufuli na kwa tarakilishi iliyowekewa vifunguo vya siri. Ni watafiti tu watakaokuwa na ruhusa ya kuona taarifa za utafiti. Jina lako na namba ya nyumba yako itakuwa na taarifa hizi lakini haitakuwa kwa ripoti yoyote. Sampuli ya damu yako itabandikiwa nambari fulani ya kisiri. Wakati utafiti utakapokamilika, tutaondoa jina lako na nambari ya nyumba yako kwa taarifa zako ili hakuna atakayetambua taarifa zako wala sampuli yako ya damu.

Nani ameidhinisha huu uatafiti ili uendelee?

Utafuti huu umeidhinishwa na mabaraza huru ya kimataifa ya Uingereza, Uholanzi, na mabaraza huru ya kitaifa yaliyo Nairobi, na kamati iliyo Kisumu ili kuhakikisha utafiti unafanywa kwa ustadi na usalama na haki za washiriki ziheshimiwe.

Utafiti huu unagharamiwa na KEMRI SSC/ERC, Chuo Kikuu cha Radboud Nijmegan, na Chuo Kikuu cha Usafi na Utafiti wa Magonjwa ya Kusambazwa ya London (LSHTM), ambao watalipia matibabu na kutoa fidia ikiwa kwa bahati mbaya madhara yatatokea.

**Nikiwa na swali ninafanyaje?**

Unaweza kumwuliza yeyote kati ya wanaoendesha utafiti maswali wakati wowote. Namna za kawasiliana nao imepeanwa mwanzoni wa maelezo haya. Unaweza pia kuwasiliana na wale wanaohusika na huduma kwa mtoto wako kwa huu utafiti:

Dkt. Jennifer Stevenson, akiwa KEMRI/CDC-S.L.P. 1578- 40100, Kisumu, Kenya

Simu: +254 713 510 845, *au*

Elizabeth Marube (daktari ya magonjwa ya binadamu) akiwa KEMRI/CDC - S.L.P. 1578-40100, Kisumu, Kenya. Simu: +254 722282097

Ukiwa na maswali kuhusu haki zako kama nyumba mshiriki, tashwishi kuhusu utafiti wenywe, au ukiwa unataka kuuliza mtu asiyeegemea utafiti huu kabisa kuhusu huu utafiti, wasiliana na:

Afisa wa utangamano na jamii, George Ben Okoth wa KEMRI/CDC – S.L.P. 1578-40100, Kisumu, Kenya. Simu: +254 725956512

*Au*

Mwenyekiti, Kamitii la Kitaifa ya Haki na Usawa wa Uendeshaji wa Utafiti (KEMRI/National ERC) - S.L.P. 54840-00200, Nairobi. Simu: 020 272 2541

Ukikubali kushiriki kwa huu utafiti tutakuuliza utie kidole kwa fomu zifuatazo za kutoa ruhusa yako na/au ya mtoto wako.

**Appendix B: Consent and Assent forms**

**Appendix B1: Household Consent for IRS and LLIN distribution**

**English**

**Highland MTC**- Reducing the burden of malaria by targeting hotspots of transmission and improving malaria control measures in the highlands of Western Kenya: Simultaneous rollout of four malaria control interventions and evaluation by cross-sectional surveys.

| Today’s date | _ _ /_ _ / _ _ _ _ | Study/Cell/Compound/ House code | V2__/ __ __ __ __/__ __ __ |
| --- | --- | --- | --- |
| Head of House: First name ___________________________ | | Middle name  _______________________ | Last name  _____________________ |

Your compound is located in an area of Rachuonyo that is exposed to intense malaria. It is therefore selected for intensive malaria control that is given in addition to those malaria interventions already provided by the Kenyan Ministry of Health. As part of this study, we will provide your compound with two mosquito control measures. For both of them you are asked to give consent separately. You can participate in both or one of the interventions; we recommend you participate in all to maximize the protection of your household. Signing this consent form will allow us to carry out LLIN distribution and IRS in your house as detailed in the information sheet. In summary we will:

1. Count the number of sleeping spaces in your house and give one long lasting net per sleeping space.
2. Spray your house and other buildings in the compound with insecticides that are routinely used for indoor residual spraying in the area by the MoH. This spraying will be repeated every 6 months and conducted by trained staff.

I, ________________________________ have had the research explained to me. I have understood all that has been read and had my questions answered satisfactorily. I understand that I can change my mind at any stage and it will not affect the benefits due to my household. Mark one box with **Y/N**:

| 1. **I CONSENT FOR LLINs TO BE PROVIDED AS DESCRIBED IN THE INFORMATION SHEET ABOVE:** |  |  |
| --- | --- | --- |
| 1. **I CONSENT FOR IRS TO BE CARRIED OUT IN MY HOUSE AND OTHER STRUCTURES AS DESCRIBED IN THE INFORMATION SHEET ABOVE:** |  |  |

I certify that I have followed all the study specific procedures described in the SOP for obtaining informed consent.

**Investigator’s name:____________**_____

**Investigator’s signature:___**__________

**Date: _________________**

**Adult/mature minor: Name____________________**

**Signature/ thumb print**: ______________________

**Date** ________________

***Only necessary if the parent/guardian cannot read:***

I *attest that the information concerning this research was accurately explained to and apparently understood by the subject and that informed consent was freely given by the subject.

**Witness’ name**: _____________________________________

**Witness’ signature:** _____________________________________ **Date** _____________

***A witness is a person who is independent from the study**

Thumbprint of the adult/parent as named above if they cannot write _____________________

**THE SUBJECT/PARENT/GUARDIAN SHOULD NOW BE GIVEN A SIGNED COPY TO KEEP**

**Mamoko B: Oboke mag Ayie**

**Mamoko B1: Oboke mar Ayie mar Goyo Udi Yath kod Pogo Nede Madak**

**Dholuo**

**Highland MTC-** Duoko pek mar maleria piny ka idimbo dago ma landruok tuo ng’enyie kod jiwo yore mag mag gayo malerie e hosi mag gode man milambo mar Kenya: Riwo yore ang’wen mag gayo maleria kod nonro margi ma dichiel.

| Tarik ma kawuono | _ _ /_ _ / _ _ _ _ | Study/Cell/Compound/House code | V2__/ __ __ __ __/__ __ __ |
| --- | --- | --- | --- |
| Wuon ot: Nying mokwongo ___________________________ | | Nying madiere  _______________________ | Nying mogik  _____________________ |

Dalani nitie ei Rachuonyo kama malaria ng’enyie. Ma omiyo oyiere ne keto okange mapek mag gayo maleria ma ichiwo ka imedo ewi mago ma Migao mar Ngima Dhano ma Kenya oseketo. Kaka mano mabiro timore e nonroni, wabiro miyo dalani gi yore ariyo mag gayo suna. Ewi magi duto, ikwayi mondo ichiw ayie ne moro ka moro. Inyalo donjo e achiel kata ariyogi duto; wejiwo ni idonji e yoregi duto mondo otegi rageng’ mar odi. Keto koki e obokeni biro miyowa thuolo mar goyo odi yath kod pogo nede mag suna man gi yath madak kaka oselerni malo kae. Machiek, wabiro:

1. Kwano kuonde nindo ma nitie e odi kendo pogo net achiel man gi yath madak ne kar nindo ka kar nindo
2. Goyo yadh suna ei odi kod udi duto ma nitie e dalani gi yedhe mag kute mafuyo ma Migao mar Ngima Dhano tiyogo e aluorau kuom nego suna ei udi. Goyo yath ibironwo bang dweche auchiel auchiel kendo ibirotim kod joma nigi lony.

An, ______________________________ oselrna tiend nonroni. Asewinjo duto ma osesomna kendo penjona odwoki makare. Ang’eyo ni anyalo loko pacha saa asaya kendo ma ok bi chocho yuto ma onego oda bedgo kowuok e nonro. Ket alama mar **Y/N** e boksi achiel

| 1. **AYIE NI NEDE MADAK OCHIW E ODA KAKA OSELER E OBOKE MAN MALO KA:** |  |  |
| --- | --- | --- |
| 1. **AYIE NI ODA GI UTE MAMOKO OGOYE YADH SUNA KAKA OSELER E OBOKE MAN MALO KA:** |  |  |

**Ng’at maduong’/Rawera mopong’: Nying_____________________**

Apuodho ni aluwo yore duto mag nonro kaka ondiki ka akawo ayie mar bedo e nonro

**Nying jataa nonro:______________**__

**Sei jataa nonro:___**____________

**Tarik: ____________**

**Sei/alama mar lwedo:________________________**

**Tarik:____________________**

***Dwarore mana ka janyuol/jarit nyathi ok nyal somo:***

*Apuodho ni ler ewi nonroni ne ochiw malong’o kendo owinjore ne jagoni, ka ayie mar bedo e nonro bende ne ochiw gi jago madonjo e nonro owuon.

**Nying janeno:**  _____________________________________

**Sei mar janeno:** _____________________________________ **Tarik** _____________

***Janeno en ng’at ma ok otudore gi nonro e yo moro amora**

Alama lwet ng’at maduong’/jarit nyathi kaka ondiki malo ka kapo ni ok ginyal ndiko:

_____________________

**JANONRO/JANYUOL/JARIT NYATHI KORO IMIYO OBKE KAKA MA MOKETIE SEI MONDO OKAN**

**Nyongeza B: Fomu za ruhusa ya kushiriki**

**Nyongeza B1: Fomu ya ruhusa ya kunyunyiza nyumba dawa na kupeana neti**

**Kiswahili**

Kupunguza uzito wa ugonjwa wa malaria kwa kuelekeza juhudi mahali palipo ueneaji wa juu na kuimarisha juhudi za kupigana na malaria kwenye nyanda za juu za Kenya Magharibi: Udhibitaji wa pamoja wa njia nne za kupigana na malaria na uchunguzi wa manufaa kupitia utafiti wa safari moja moja.

| Tarehe ya leo | _ _ /_ _ / _ _ _ _ | Study/Cell/Compound/House code | V2__/ __ __ __ __/__ __ __ |
| --- | --- | --- | --- |
| Mkuu wa nyumba: Jina la kwanza ___________________________ | | Jina la katikati  _______________________ | Jina la mwisho  _____________________ |

Boma lako yako liko katika eneo la Rachuonyo lenye hupata malaria nyingi sana. Kwa hivyo imechaguliwa kutiliwa juhudi thabiti dhidi ya malaria, zenye zitaungana nguvu na juhudi zilizomo za Wizara ya Afya ya Kenya. Kama sehemu ya huu utafiti, tutakuletea mikakati miwili ya kupambana na mbu. Kwa kila mkakati, unaulizwa kutoa ruhusa tofauti. Unaweza kushiriki kwa moja amah ii mikakati miwili pamoja; tunahimiza ushiriki kwa mikakati yote ili kuinua kinga za nyumba yako. Kutia kidole kwa hii fomu kutaturuhusu kupeana neti na kunyunyizia nyumba yako dawa vile imeelezwa hapo awali. Kwa machache tuta:

1. Hesabu idadi ya mahali pa malazi nyumbani mwako, na kupeana neti moja inayodumu kwa kila malazi
2. Nyunyizia nyumba yako na majengo mengine bomani mwako dawaya madudu inayodumu yenye hutumiwa sana katika eneo hili na Wizara ya Afya. Unyunyiziaji huu utarudiwa baada ya kila miezi 6 na kufanywa na waliopewa mafunzo kamili.

Mimi,_____________________________ nimelezewa kuhusi huu utafiti. Nimeelewa kuwa nimesomewa kamili na maswali yangu kujibiwa vilivyo. Ninaelewa kuwa ninaweza kubadilisha uamuzi wangu wakati wowote nah ii haitadhuru manufaa yanayofaa nyumba yangu. Weka alama ya **Y/N** kwa kisanduku moja:

| 1. **NIMEKUBALI NETI ZIPEANWE VILE IMEELEZWA HAPA JUU:** |  |  |
| --- | --- | --- |
| 1. **NIMEKUBALI NYMBA YANGU NA MAJENGO MENGINE KUNYUNYIZIWA DAWA ILIVYOELEZWA HAPA JUU:** |  |  |

Ninadhibitisha nimefuata kanuni zote za utafiti wakati ninachukuwa ruhusa hii.

**Jina la mtafiti:___________**____

**Seii ya mtafiti:___**___________

**Tarehe ______________________**

**Mtu mzima/mtoto mkubwa: Jina_______________________**

**Seii/alama ya kidole_____________________**

**Tarehe________________**

***Muhimu ikiwa mzazi/mlezi wa mtoto hawezi kusoma:***

Ninadhibitisha kuwa taarifa juu ya huu utafiti ulielezwa kamili na mshiriki kuelewa na kwamba ruhusa hii imetolewa kwa hiari.

**Jina la shahidi**: _________________________________________

**Seii la shahidi**: _________________________________________ **Tarehe ___________**

*** Shahidi ni mtu asiyeegemea utafiti kwa namna yoyote**

Alama ya kidole cha mtu mzima/mzazi ametajwa hapa juu kama hawezi kuandika: ___________________

**MSHIRIKI/MZAZI/MLEZI ANATAKIKANA SASA KUPATIWA KOPI YA KARATASI HILI ILIYOTIWA KIDOLE ILI AWAKE**

**Appendix B2. Consent form: Focal Screening and Treatment intervention (individual level) - English**

**Highland MTC** - Reducing the burden of malaria by targeting hotspots of transmission and improving malaria control measures in the highlands of Western Kenya: Simultaneous rollout of four malaria control interventions and evaluation by cross-sectional surveys.

| Today’s date | _ _ /_ _ / _ _ _ _ | Study/Cell/Compound/House code | V2__/ __ __ __ __/__ __ __ |
| --- | --- | --- | --- |
| First name ___________________________ | | Middle name  _______________________ | Last name  _____________________ |

Your compound is located in an area of Rachuonyo that is exposed to intense malaria and has been selected for intensive malaria control that will be given in addition to those malaria interventions already provided by the Kenyan Ministry of Health. Signing this form will allow us to carry out testing and treatment of all individuals in the compound as detailed in the information sheet. In summary we will take temperatures and blood samples by finger prick and test for low blood from everyone. All children between the ages of 6 month and 20 years of age will be screened for malaria with a rapid diagnostic test. All people over 20 years with a temperature will also be tested for malaria using an RDT. If anyone is found to be parasite positive, all members of the compound receive free anti-malarial treatment.

We will not be able to treat women who are pregnant in this study. Pregnant women who are sick need to go to a health facility to see a doctor. If you are a woman between 13 and 45 years of age, we will ask you questions about whether or not you might be pregnant. If you may be pregnant, but are unsure, we will ask you to do a pregnancy test here in your home from your urine sample.

I, ________________________________ have had the research explained to me. I have understood all that has been read and had my questions answered satisfactorily. I understand that I can change my mind at any stage and it will not affect the benefits due to my household.

Mark one box with **Y/N**:

| **I CONSENT TO SCREENING AND TREATMENT AS DESCRIBED IN THE INFORMATION SHEET:** |  |  |
| --- | --- | --- |

**Minor if under 13:Name**____________________

I certify that I have followed all the study specific procedures described in the SOP for obtaining informed consent.

**Investigator’s name:________________**_____

**Investigator’s signature:** _____________

**Date _______**

**Parent/guardian’s: Name____________________**

**OR**

**Adult/mature minor: Name__________________**

**Signature/thumbprint**: _________________

**Date** ___________

***Only necessary if the parent/guardian cannot read:***

I *attest that the information concerning this research was accurately explained to and apparently understood by the subject and that informed consent was freely given by the subject.

**Witness’ name**: _____________________________________

**Witness’ signature:** _____________________________________ **Date** _____________

***A witness is a person who is independent from the study**

**THE SUBJECT/PARENT/GUARDIAN SHOULD NOW BE GIVEN A SIGNED COPY TO KEEP**

We also would like store your/your child’s filter paper blood sample at KEMRI/CDC in Kisian for 5 years. We would like to test to see if there are genes that help malaria spread and make malaria hard to treat with medicine. Your blood will be stored with a unique code that is known only to the study staff. We will not screen your/your child’s blood for any other diseases. For quality control, we will test a small proportion of samples in the UK. We ask you for specific consent to ship samples abroad.

If you no longer want us to store your/your child’s blood, please contact Dr. Jennifer Stevenson or Chrispin Owaga at the above address or telephone numbers and we will destroy the sample. After the study period has ended we will remove any means to link the sample to you/your child and we will not be able to identify the sample. If you do not wish to have your/your child’s blood stored, you/your child may still participate in our study. You/your child will still be examined for malaria and low blood. If you/your child has malaria or low blood, we will still treat you/your child.

**Long term storage and future studies:**

I agree to allow KEMRI/CDC to store my/ my child’s filter paper blood sample for future studies. I understand that I can change my mind to not have my/my child’s filter paper blood sample stored and used for future research during the next 5 years. I may also ask that my/my child’s filter paper blood sample not be used for certain types of testing. To do this, I may tell Dr. Jennifer Stevenson of my request and she will tell the study staff at KEMRI/CDC

Mark one box with **X**:

| **I DO CONSENT:** |  | **I hereby agree to allow storage of my blood samples for future studies** |
| --- | --- | --- |

| **I DO NOT CONSENT:** |  | **I do not allow storage of my blood samples for future studies** |
| --- | --- | --- |

I certify that I have followed all the study specific procedures described in the SOP for obtaining informed consent.

**Investigator’s name:_____________**____

**Investigator’s signature:** ___________

**Date _______**

**Minor if under 13:Name**____________________

**Parent/guardian’s: Name____________________**

**OR**

**Adult/mature minor: Name__________________**

**Signature/thumbprint**: _________________

**Date** ___________

***Only necessary if the parent/guardian cannot read:***

I *attest that the information concerning this research was accurately explained to and apparently understood by the subject/parent/guardian and that informed consent was freely given by the subject/parent/guardian.

**Witness’ name**: _____________________________________

**Witness’ signature:** _____________________________________ **Date** _____________

***A witness is a person who is independent from the study**

**THE SUBJECT/PARENT/GUARDIAN SHOULD NOW BE GIVEN A SIGNED COPY TO KEEP**

**Momedi B2: Oboke mar Ayie: Pimo kod thieth modimbi (ng’ato owuon)**

**Dholuo**

**Highland MTC-** Duoko pek mar maleria piny ka idimbo dago ma landruok tuo ng’enyie kod jiwo yore mag mag gayo malerie e hosi mag gode man milambo mar Kenya: Riwo yore ang’wen mag gayo maleria kod nonro margi ma dichiel.

| Tarik ma kawuono | _ _ /_ _ / _ _ _ _ | Study/Cell/Compound/House code | V2__/ __ __ __ __/__ __ __ |
| --- | --- | --- | --- |
| Wuon ot: Nying mokwongo ___________________________ | | Nying madiere  _______________________ | Nying mogik  _____________________ |

Dalau nitie ei Rachuonyo kama malaria ng’enyie. Ma omiyo oyiere ne keto okange mapek mag gayo maleria ma ichiwo ka imedo ewi mago ma Migao mar Ngima Dhano ma Kenya oseketo. Kaka mano mabiro timore e nonroni, wabiro miyo dalani gi yore ariyo mag gayo suna. Keto koki e obokeni biro miyowa thuolo mar pimo kod thiedho ji duto e dalani kaka oselerni malo kae. Machiek, wabiro pimo liet mar del kod kawo remo ewi lith lwedo kendo tin mar remo ne ji duto. Jogo duto ma hikgi en dweche 6 nyaka higni 20 ibiropimnegi malaria gi yo mapiyo ahinya. Ji duto ma hikgi ohingo 20 to dendgi liet bende ibirotimnegi malaria gi RDT. Ka ng’ato oyudi gi malaria, ji duto mantie e dalano ibiro thiedhi negi malaria maonge chudo.

Ok wabi thiedho mine man gi ich e nonroni e dala nikech Coartem ok opuodhi tiyogo kuom mon ma yach. Ka in nyako/dhako ma hike nitie kind higni 13-45,to wabiro penji ka dibed kod ich. Ka dipo ni ipek to ionge gi adier, wabiro kwayo mondo ipimri e dala ka itiyo gi lachni.

An, ______________________________ oselrna tiend nonroni. Asewinjo duto ma osesomna kendo penjona odwoki makare. Ang’eyo ni anyalo loko pacha saa asaya kendo ma ok bi chocho yuto ma onego oda bedgo kowuok e nonro. Ket alama mar **Y/N** e boksi achiel

| **AYIE NE PIM KOD THIETH KAKA OLER E OBOKE:** |  |  |
| --- | --- | --- |

**Nyathi ka hike tin ne 13: Nying____________________**

Apuodho ni aluwo yore duto mag nonro kaka ondiki ka akawo ayie mar bedo e nonro.

**Nying jataa nonro:_____________**

**Sei jataa nonro:___**____________

**Tarik ________________**

**Janyuol/Jarit nyathi: Nying________________________**

**KATA**

**Ng’at maduong’/Rawera mopong’: Nying_______________**

**Sei/alama mar lwedo:_________________**

**Tarik_________**

***Dwarore mana ka janyuol/jarit nyathi ok nyal somo:***

*Apuodho ni ler ewi nonroni ne ochiw malong’o kendo owinjore ne jagoni, ka ayie mar bedo e nonro bende ne ochiw gi jago madonjo e nonro owuon.

**Nying janeno:**  _____________________________________

**Sei mar janeno:** _____________________________________ **Tarik** _____________

***Janeno en ng’at ma ok otudore gi nonro e yo moro amora**

**JANONRO/JANYUOL/JARIT NYATHI KORO IMIYO OBKE KAKA MA MOKETIE SEI MONDO OKAN**

Wadwaro bende kano rembi/remb nyathini ma nitie e otas ma kende kar KEMRI/CDC man Kisian kuom higni abich.wabiro dwaro pimo ka nitie kido moko konyo malaria landore kendo miyo obedo matek thiedho gi yien. Rembi ibirokan gi namba moro ma kende ma jota nonro kende ema ong’eyo. Ok wabipimo tuoche mamoko e rembi/remb nyathini. Mar mondo wane ka pim gi dhi maber, wabiro oro moko matin mar remogi loka UK. Wakwayi mondo imiwa ayie ma kende mar oro remo loka.

Ka e thuolo mar nonro, ok idwar ni wamed kano rembi/remb nyathini, yie itudori kod Lkt. Jennifer Stevenson kat Chrispin Owaga kaka onyisi malo kae, bang’e to wabiro wito remogo e yo makare. Bang’ rumo nonro to wabiro golo yo moro amora ma nyalo tudo remono kodi/nyathini kendo ok wabinyalo bidho weg remogo.kata ka ok idwar ni okan rembi/remb nyathini to pod in/nyathini nyalo donjo e nonro. In/nyathini pod ibirotimne pim mar malaria kod remo matin. Ka in/nyathini nitie kod malaria kata remo matin, pod wabiro thiedhi/nyathini.

**Kano remo kuom thuolo ma lach ne nonro ma bang’e:**

Ayie ne KEMRI/CDC mondo okan remba/remb nyathina mantie e otas ne nonro ma bang’e. Ang’eyo ni anyalo loko pacha mondo kik kan remba/remb nyathina matie e otas ne nonro ma bang’e e thuolo mar higni abich mabiro. Anyalo bende kwayo ni remba/remb nyathina ok ti godo e pim moko. Mondo atim ma, anyalo nyiso Lkt. Jennifer Stevenson kwayona kendo obiro nyiso jota nonro man KEMRI/CDC.

Ket alama mar **X** e boksi achiel

| **AYIE:** |  | **Ayie mondo okan remba ne nonro mabiro bang’e** |
| --- | --- | --- |

| **OK AYIE:** |  | **Ok ayie mondo okan remba ne nonro mabiro bang’e** |
| --- | --- | --- |

Apuodho ni aluwo yore duto mag nonro kaka ondiki ka akawo ayie mar bedo e nonro.

**Nying jataa nonro:______________**

**Sei jataa nonro:___**____________

**Tarik ________________**

**Nyathi ka hike tin ne 13: Nying____________________**

**Janyuol/Jarit nyathi: Nying________________________**

**KATA**

**Ng’at maduong’/Rawera mopong’: Nying_______________**

**Sei/alama mar lwedo:_________________ Tarik_________**

***Dwarore mana ka janyuol/jarit nyathi ok nyal somo:***

*Apuodho ni ler ewi nonroni ne ochiw malong’o kendo owinjore ne jagoni, ka ayie mar bedo e nonro bende ne ochiw gi jago madonjo e nonro owuon.

**Nying janeno:**  _____________________________________

**Sei mar janeno:** _____________________________________ **Tarik** _____________

***Janeno en ng’at ma ok otudore gi nonro e yo moro amora**

**JANONRO/JANYUOL/JARIT NYATHI KORO IMIYO OBKE KAKA MA MOKETIE SEI MONDO OKAN**

**Nyongeza B2: Fomu ya ruhusa: Mkakati wa Kupima na Kutibu (watu binafsi)**

**Kiswahili**

Kupunguza uzito wa ugonjwa wa malaria kwa kuelekeza juhudi mahali palipo ueneaji wa juu na kuimarisha juhudi za kupigana na malaria kwenye nyanda za juu za Kenya Magharibi: Udhibitaji wa pamoja wa njia nne za kupigana na malaria na uchunguzi wa manufaa kupitia utafiti wa safari moja moja.

| Tarehe ya leo | _ _ /_ _ / _ _ _ _ | Study/Cell/Compound/House code | V2__/ __ __ __ __/__ __ __ |
| --- | --- | --- | --- |
| Mkuu wa nyumba: Jina la kwanza ___________________________ | | Jina la katikati  _______________________ | Jina la mwisho  _____________________ |

Boma lako yako liko katika eneo la Rachuonyo lenye hupata malaria nyingi sana. Kwa hivyo imechaguliwa kutiliwa juhudi thabiti dhidi ya malaria, zenye zitaungana nguvu na juhudi zilizomo za Wizara ya Afya ya Kenya. Kama sehemu ya huu utafiti, tutakuletea mikakati miwili ya kupambana na mbu. Kwa kila mkakati, unaulizwa kutoa ruhusa tofauti. Unaweza kushiriki kwa moja amah ii mikakati miwili pamoja; tunahimiza ushiriki kwa mikakati yote ili kuinua kinga za nyumba yako. Kutia kidole kwa hii fomu kutaturuhusu kupima na kutibu watu wote walioko bomani mwako vile imeelezwe hapa juu. Kwa ufupi tutapima joto la mwili na kuchukuwa sampuli ya damu kwa kidole na kupima upungufu wa damu kwa watu wote. Watoto wote wenye umri wa miezi 6 hadi miaka 20 watachunguziwa malaria kutumia mbinu ya upesi (RDT). Watu wote wliozidi umri wa miaka 20 wenye joto jingi pia watachunguziwa malaria kwa RDT. Mtu yeyote akipatikana na malaria, watu wote wa hilo boma watatibiwa dhidi ya malaria bila malipo.

Hatutaweza kuwatibu wanawake wajaa wazito kwa huu utafiti. Wanawake wajaa wazito wenye ni wagonjwa watahitajika kwenda kwa kituo cha afya kumwona daktari. Ukiwa mwanamke aliye na umri wa miaka 13- miaka 45, tutakuuliza maswali kuhusu uwezekano wa kuwa na mimba. Ukiwa unaweza kuwa mjaa mzito na hauna uhakika, tutakuuliza ufanye uchunguzi wa kuwa na mimba hapa nyumbani ukitumia mkojo wako.

Mimi,____________________ nimelezewa kuhusi huu utafiti. Nimeelewa kuwa nimesomewa kamili na maswali yangu kujibiwa vilivyo. Ninaelewa kuwa ninaweza kubadilisha uamuzi wangu wakati wowote nah ii haitadhuru manufaa yanayofaa nyumba yangu.

Weka alama ya **Y/N** kwa kisanduku moja:

| **NIMEPATIANA RUHUSA YA KUPIMWA NA KUTIBIWA ILIVYOELEZWA HAPA JUU:** |  |  |
| --- | --- | --- |

Ninadhibitisha nimefuata kanuni zote za utafiti wakati ninachukuwa ruhusa hii.

**Jina la mtafiti:______________**____

**Seii ya mtafiti:___**_______________

**Tarehe: _______________________**

**Mtoto kama chini ya 13: Jina______________________**

**Mzazi/Mlezi: Jina___________________**

**AU**

**Mtu mzima/mtoto mkubwa: Jina__________________**

**Seii/alama ya kidole________________**

**Tarehe____________**

***Muhimu ikiwa mzazi/mlezi wa mtoto hawezi kusoma:***

Ninadhibitisha kuwa taarifa juu ya huu utafiti ulielezwa kamili na mshiriki kuelewa na kwamba ruhusa hii imetolewa kwa hiari.

**Jina la shahidi**: _________________________________________

**Seii la shahidi**: _________________________________________ **Tarehe ___________**

*** Shahidi ni mtu asiyeegemea utafiti kwa namna yoyote**

**MSHIRIKI/MZAZI/MLEZI ANATAKIKANA SASA KUPATIWA KOPI YA KARATASI HILI ILIYOTIWA KIDOLE ILI AWAKE**

Tungependa pia kuhifadhi sampuli ya damu yako/damu ya mtoto wako ilioyoko kwa kartatasi huko KEMRI/CDC kwa miaka mitano. Tungependa kupima ili tuone ikiwa kuna majijni fulani ambayo husaidia malaria kuenea na kuisababissha ngumu kutibu. Damu yako/damu yamtoto wako itahifadhiwa na nambari fulani ya siri inayujulikana tu kwa watafiti husika. Hatutachunguza damu yako/damu ya mtoto wako kwa magonjwa mengine. Ili kudhibitisha ubora wa uchunguzi tunaofanya, kiasi kidogo cha sampuli kitatumwa kwa uchunguzi Uingereza. Tunakuuliza ruhusa ya kipekeee ya kutuma sampuli ng’ambo.

Ikiwa wakati utafiti unapoendelea, utataka tusiendelee kuhifadhi damu yako/ya mtoto wako, tafadhali wasiliana na Dkt. Jennifer Stevenson au Chrispin Owaga kwa njia ya mawasiliano iliyo hapa juu na tutaharibu sampuli yako.ifikapo tamati ya uchunguzi, tuaondoa namna yote inaweza kukulinganisha/mtoto wako na sampuli, na hatutaweza kuitambua sampuli hiyo. Kama hutaki damu yako/ya mtoto wako kuhifadhiwa, wewe/mtoto wako bado wanaweza kuendelea kushiriki kwa utafiti. Wewe/mtoto wako bado atachunguziwa malaria na upungufu wa damu. Ukiwa/akiwa na malaria au upungufu wa damu, bado tutakutibu/tutamtibu mtoto wako.

**Uhufadhi wa sampuli na utafiti za baadaye:**

Naipatia KEMRI/CDC ruhusa ya kuhifadhi damu yangu/ya mtoto wangu iliyo kwa karatasi maalum kwa utafiti za baadaye. Ninaelewa kuwa ninaweza kubadilisha uamuzi wangu ili damu yangu/ya mtoto wangu isihifadhiwe kwa uchunguzi kwa kipindi cha miaka mitano ifuatayo. Ninaweza pia kuuliza kwamba damu yangu/ya mtoto wangu iliyo kwa karatasi maalum isitumike kwa aina fulani ya uchunguzi. Ili nifanye hivi, ninaweza kumjulisha Dkt. Jennifer Stevenson nia yangu na atawajulisha wahusika kule KEMRI/CDC.

Weka alama ya **X** katika kisanduku kimoja:

| **NIMEKUBALI:** |  | | **Nimekubali damu yangu ihifadhiwe kwa utafiti za baadaye** | |  |
| --- | --- | --- | --- | --- | --- |
| **SIJAKUBALI:** | |  | | **Sijakubali damu yangu ihifadhiwe kwa utafiti za baadaye** | |

**Mtoto kama chini ya 13: Jina__________________**

Ninadhibitisha nimefuata kanuni zote za utafiti wakati ninachukuwa ruhusa hii.

**Jina la mtafiti:______________**____

**Seii ya mtafiti:___**_______________

**Tarehe _______________________**

**Mzazi/Mlezi: Jina____________________________**

**AU**

**Mtu mzima/mtoto mkubwa: Jina_______________**

**Seii/alama ya kidole__________________**

**Tarehe___________**

***Muhimu ikiwa mzazi/mlezi wa mtoto hawezi kusoma:***

Ninadhibitisha kuwa taarifa juu ya huu utafiti ulielezwa kamili na mshiriki kuelewa na kwamba ruhusa hii imetolewa kwa hiari.

**Jina la shahidi**: __________________________________

**Seii la shahidi**: __________________________________ **Tarehe ___________**

*** Shahidi ni mtu asiyeegemea utafiti kwa namna yoyote**

**MSHIRIKI/MZAZI/MLEZI ANATAKIKANA SASA KUPATIWA KOPI YA KARATASI HILI ILIYOTIWA KIDOLE ILI AWAKE**

**Appendix B3: Assent form: Focal Screen and Treat intervention (individual level)**

**English**

**Highland MTC**- Reducing the burden of malaria by targeting hotspots of transmission and improving malaria control measures in the highlands of Western Kenya: Simultaneous rollout of four malaria control interventions and evaluation by cross-sectional surveys.

| Today’s date | _ _ /_ _ / _ _ _ _ | Study/Cell/Compound/House code | V2__/ __ __ __ __/__ __ __ |
| --- | --- | --- | --- |
| Head of House: First name ___________________________ | | Middle name  _______________________ | Last name  _____________________ |

- I am being asked to decide if I want to be in this research study.
- I am being asked if I want to be tested for malaria
- I know I will have a few drops of blood drawn from my finger and have my temperature taken
- If I have malaria or someone in my compound has malaria I will also receive medicine for malaria even if I do not have malaria
- I may be given medicine for malaria even if I am not feeling sick today.
- I asked and got answers to my questions. I know that I can ask questions about this study at any time.
- I know that I can stop being in this study at anytime without anyone being mad at me.

Mark box with **Y/N**:

| **I CONSENT TO SCREENING AND TREATMENT AS DESCRIBED IN THE INFORMATION SHEET:** |  |  |
| --- | --- | --- |

**Child (13-17):Name**__________________

I certify that I have followed all the study specific procedures described in the SOP for obtaining informed consent.

**Investigator’s name:_____________**___

**Investigator’s signature:** ___________

**Date ______**

**Signature/thumbprint**: ______________**Date** __________

**Parent/guardian’s: Name_________________**

**Signature/thumbprint**: ________________

**Date** ___________

***Only necessary if the parent/guardian cannot read:***

I *attest that the information concerning this research was accurately explained to and apparently understood by the subject/parent/guardian and that informed consent was freely given by the subject/parent/guardian.

**Witness’ name**: _____________________________________

**Witness’ signature:** _____________________________________ **Date** _____________

***A witness is a person who is independent from the study**

**THE SUBJECT/PARENT/GUARDIAN SHOULD NOW BE GIVEN A SIGNED COPY TO KEEP**

**Momedi B3: Oboke mar Ayie ewi nyithindo: Pimo kod thieth modimbi (ng’ato owuon)**

**Dholuo**

**Highland MTC-** Duoko pek mar maleria piny ka idimbo dago ma landruok tuo ng’enyie kod jiwo yore mag mag gayo malerie e hosi mag gode man milambo mar Kenya: Riwo yore ang’wen mag gayo maleria kod nonro margi ma dichiel.

| Tarik ma kawuono | _ _ /_ _ / _ _ _ _ | Study/Cell/Compound/House code | V2__/ __ __ __ __/__ __ __ |
| --- | --- | --- | --- |
| Wuon ot: Nying mokwongo ___________________________ | | Nying madiere  _______________________ | Nying mogik  _____________________ |

- Ikwaya ni awachi ka adwaro donjo e nonro
- Ikwaya ka adwaro ni mondo opimna maleria
- Ang’eyo ni ibirokaw remo mtin ahinya e lith lweta kendo ibiropim liet mar denda
- Ka na kata ng’ato ang’ata nitie gi maleria e dalana to abiro yudo thieth mar maleria kata obedo ni aonge gi maleria
- Inyalo miya yadh maleria kata ka ok awing matuo kawuono
- Ne apenjo kendo ayudo dwoko mag penjona. Ang’eyo ni anyalo penjo ewi nonroni saa asaya
- Ang’eyo ni anyalo weyo bedo e nonroni ma onge ng’at makelo koko moro amora

Ket alama mar **Y/N** e boksi achiel

| **AYIE NE PIM KOD THIETH KAKA OLER E OBOKE:** |  |  |
| --- | --- | --- |

**Nyathi (13-17): Nying________________________**

Apuodho ni aluwo yore duto mag nonro kaka ondiki ka akawo ayie mar bedo e nonro.

**Nying jataa nonro:___________**____

**Sei jataa nonro:___**____________

**Tarik ________________**

**Sei/alama mar lwedo:_________________**

**Tarik__________**

**Janyuol/Jarit nyathi: Nying_______________**

**Sei/alama mar lwedo:_________________**

**Tarik__________**

***Dwarore mana ka janyuol/jarit nyathi ok nyal somo:***

*Apuodho ni ler ewi nonroni ne ochiw malong’o kendo owinjore ne jagoni, ka ayie mar bedo e nonro bende ne ochiw gi jago madonjo e nonro owuon.

**Nying janeno:**  _____________________________________

**Sei mar janeno:** _____________________________________ **Tarik** _____________

***Janeno en ng’at ma ok otudore gi nonro e yo moro amora**

**JANONRO/JANYUOL/JARIT NYATHI KORO IMIYO OBKE KAKA MA MOKETIE SEI MONDO OKAN**

**Nyongeza B3: Fomu ya ruhusa: Mkakati wa Kupima na Kutibu watoto (watu binafsi)**

**Kiswahili**

Kupunguza uzito wa ugonjwa wa malaria kwa kuelekeza juhudi mahali palipo ueneaji wa juu na kuimarisha juhudi za kupigana na malaria kwenye nyanda za juu za Kenya Magharibi: Udhibitaji wa pamoja wa njia nne za kupigana na malaria na uchunguzi wa manufaa kupitia utafiti wa safari moja moja.

| Tarehe ya leo | _ _ /_ _ / _ _ _ _ | Study/Cell/Compound/House code | V2__/ __ __ __ __/__ __ __ |
| --- | --- | --- | --- |
| Mkuu wa nyumba: Jina la kwanza ___________________________ | | Jina la katikati  _______________________ | Jina la mwisho  _____________________ |

- Ninaulizwa kuamua ikiwa ningependa kushiriki kwa utafiti
- Ninaulizwa ikiwa ninataka kupimiwa maleria
- Nitatolewa damu kidogo kwa kidole na kupimiwa joto la mwili
- Nikipatikana na maleria au mtu yeyote katika boma langu akipatikana na maleria nitapokea dawa za maleria hata kama sina maleria
- Ninaweza kupewa dawa za maleria hata kama sihisi mgonjwa leo hii
- Niliuliza maswali nikajibiwa. Ninajua ninaweza uliza maswali kuhusu huu utafiti wakati wowote
- Ninaju naweza kujiondoa kwa utafiti bila yeyote kuniletea maneno

Weka alama ya **Y/N** kwa kisanduku moja:

| **NIMEKUBALI KUPIMWA NA KUTIBIWA ILVYOELEZWA HAPA JUU:** |  |  |
| --- | --- | --- |

Ninadhibitisha nimefuata kanuni zote za utafiti wakati ninachukuwa ruhusa hii.

**Jina la mtafiti:______________**____

**Seii ya mtafiti:___**_______________

**Tarehe _______________________**

**Mtoto (13-17): Jina**__________________________

**Seii/alama ya kidole_____________________**

**Tarehe________________**

***Muhimu ikiwa mzazi/mlezi wa mtoto hawezi kusoma:***

Ninadhibitisha kuwa taarifa juu ya huu utafiti ulielezwa kamili na mshiriki kuelewa na kwamba ruhusa hii imetolewa kwa hiari.

**Jina la shahidi**: _________________________________________

**Seii la shahidi**: _________________________________________ **Tarehe ___________**

*** Shahidi ni mtu asiyeegemea utafiti kwa namna yoyote**

**MSHIRIKI/MZAZI/MLEZI ANATAKIKANA SASA KUPATIWA KOPI YA KARATASI HILI ILIYOTIWA KIDOLE ILI AWAKE**

**Appendix B4. Consent form: Community malaria surveys (individual level)**

**English**

**Highland MTC**- Reducing the burden of malaria by targeting hotspots of transmission and improving malaria control measures in the highlands of Western Kenya: Simultaneous rollout of four malaria control interventions and evaluation by cross-sectional surveys.

| Today’s date | _ _ /_ _ / _ _ _ _ | Study/Cell/Compound/House code | V2__/ __ __ __ __/__ __ __ |
| --- | --- | --- | --- |
| First name ___________________________ | | Middle name  _______________________ | Last name  _____________________ |

Your compound is located in an area of Rachuonyo that is exposed to intense malaria. We would like to carry out a study in your compound to see whether malaria control that has been recently implemented has been effective in reducing malaria. Signing this form will allow us to interview you and/or you child, take measurements of temperature and collect blood samples by fingerprick to test for malaria in the compound or in laboratories in Kisumu, the UK and the Netherlands. If anyone is found to be parasite positive, they will receive anti-malarials free of charge. And everyone who has low blood will receive iron.

We will not be able to treat women who are pregnant in this study. Pregnant women who are sick need to go to a health facility to see a doctor. If you are a woman between 13 and 45 years of age, we will ask you questions about whether or not you might be pregnant. If you may be pregnant, but are unsure, we will ask you to do a pregnancy test here in your home from your urine sample.

I, ________________________________ have had the research explained to me. I have understood all that has been read and had my questions answered satisfactorily. I understand that I can change my mind at any stage and it will not affect the benefits due to my household. Mark one box with **Y/N**:

| **I CONSENT TO BE INTERVIEWED AND BLOOD SAMPLE TAKEN AS DESCRIBED IN THE INFORMATION SHEET:** |  |  |
| --- | --- | --- |

I certify that I have followed all the study specific procedures described in the SOP for obtaining informed consent.

**Investigator’s name:________________**_

**Investigator’s signature:** ____________

**Date: ______________**

**Minor if under 13:Name**____________________

**Parent/guardian’s: Name____________________**

**OR**

**Adult/mature minor: Name__________________**

**Signature/thumbprint**: _________________

**Date:** ___________

***Only necessary if the parent/guardian cannot read:***

I *attest that the information concerning this research was accurately explained to and apparently understood by the subject and that informed consent was freely given by the subject.

**Witness’ name**: _________________________

**Witness’ signature:** ______________________ **Date** _____________

***A witness is a person who is independent from the study**

**THE SUBJECT/PARENT/GUARDIAN SHOULD NOW BE GIVEN A SIGNED COPY TO KEEP**

We also would like store your/your child’s filter paper blood sample at KEMRI/CDC in Kisian for 5 years. We would like to test to see if there are genes that help malaria spread and make malaria hard to treat with medicine. Your blood will be stored with a unique code that is known only to the study staff. We will not screen your/your child’s blood for any other diseases. For quality control, we will test a small proportion of samples in the UK. We ask you for specific consent to ship samples abroad.

If you no longer want us to store your/your child’s blood, please contact Dr. Jennifer Stevenson or Chrispin Owaga at the above address or telephone numbers and we will destroy the sample. After the study period has ended we will remove any means to link the sample to you/your child and we will not be able to identify the sample. If you do not wish to have your/your child’s blood stored, you/your child may still participate in our study. You/your child will still be examined for malaria and low blood. If you/your child has malaria or low blood, we will still treat you/your child.

**Long term storage and future studies:**

I agree to allow KEMRI/CDC to store my/ my child’s filter paper blood sample for future studies. I understand that I can change my mind to not have my/my child’s filter paper blood sample stored and used for future research during the next 5 years. I may also ask that my/my child’s filter paper blood sample not be used for certain types of testing. To do this, I may tell Dr. Jennifer Stevenson of my request and she will tell the study staff at KEMRI/CDC

Mark one box with **X**:

| **I DO CONSENT:** |  | **I hereby agree to allow storage of my blood samples for future studies** |
| --- | --- | --- |

| **I DO NOT CONSENT:** |  | **I do not allow storage of my blood samples for future studies** |
| --- | --- | --- |

I certify that I have followed all the study specific procedures described in the SOP for obtaining informed consent.

**Investigator’s name:__________**_____

**Investigator’s signature:** ____________

**Date _______**

**Minor if under 13:Name**____________________

**Parent/guardian’s: Name____________________**

**OR**

**Adult/mature minor: Name__________________**

**Signature/thumbprint**: _________________

**Date** ___________

***Only necessary if the parent/guardian cannot read:***

I *attest that the information concerning this research was accurately explained to and apparently understood by the subject/parent/guardian and that informed consent was freely given by the subject/parent/guardian.

**Witness’ name**: _____________________________________

**Witness’ signature:** _____________________________________ **Date** _____________

***A witness is a person who is independent from the study**

**THE SUBJECT/PARENT/GUARDIAN SHOULD NOW BE GIVEN A SIGNED COPY TO KEEP**

**Momedi B4: Oboke mar Ayie: Pimo ng’eny maleria e oganda (ng’ato owuon)**

**Dholuo**

**Highland MTC-** Duoko pek mar maleria piny ka idimbo dago ma landruok tuo ng’enyie kod jiwo yore mag mag gayo malerie e hosi mag gode man milambo mar Kenya: Riwo yore ang’wen mag gayo maleria kod nonro margi ma dichiel.

| Tarik ma kawuono | _ _ /_ _ / _ _ _ _ | Study/Cell/Compound/House code | V2__/ __ __ __ __/__ __ __ |
| --- | --- | --- | --- |
| Wuon ot: Nying mokwongo ___________________________ | | Nying madiere  _______________________ | Nying mogik  _____________________ |

Dalani nitie ei Rachuonyo kama malaria ng’enyie. Wadwaro timo nonro e dalani mondo wane ka yore ma gayo malaria ma osekiti e kinde mokalo oseduoko ng’eny malaria piny. Keto koki e obokeni biro miyowa thuolo mar miyi/kata nyathini penjo moko, pimo liet mar del, kod kawao remo ewi lith lwedo mondo wapimgo malaria e dalani kata e lab magwa man Kisumo, UK kod Netherlands. Ng’at ma oyudi gi malaria ibiro thiedhi maonge chudo. Ji duto ma oyudi gi remo matin bende ibiro thiedhi gi yath daduoko remo.

Ok wabi thiedho mine man gi ich e nonroni e dala nikech Coartem ok opuodhi tiyogo kuom mon ma yach. Ka in nyako/dhako ma hike nitie kind higni 13-45 to wabiro penji ka dibed kod ich. Ka dipo ni ipek to ionge gi adier, wabiro kwayo mondo ipimri e dala ka itiyo gi lachni.

An, ______________________________ oselrna tiend nonroni. Asewinjo duto ma osesomna kendo penjona odwoki makare. Ang’eyo ni anyalo loko pacha saa asaya kendo ma ok bi chocho yuto ma onego oda bedgo kowuok e nonro.

Ket alama mar **Y/N** e boksi achiel

| **AYIE DUOKO PENJO GI GOLO REMO KAKA OLER E OBOKE:** |  |  |
| --- | --- | --- |

Apuodho ni aluwo yore duto mag nonro kaka ondiki ka akawo ayie mar bedo e nonro.

**Nying jataa nonro:_______**_______

**Sei jataa nonro:___**____________

**Tarik: ________________**

**Nyathi ka hike tin ne 13: Nying____________________**

**Janyuol/Jarit nyathi: Nying_______________________**

**KATA**

**Ng’at maduong’/Rawera mopong’: Nying_______________**

**Sei/alama mar lwedo:_________________ Tarik____________**

***Dwarore mana ka janyuol/jarit nyathi ok nyal somo:***

*Apuodho ni ler ewi nonroni ne ochiw malong’o kendo owinjore ne jagoni, ka ayie mar bedo e nonro bende ne ochiw gi jago madonjo e nonro owuon.

**Nying janeno:**  _____________________________________

**Sei mar janeno:** _____________________________________ **Tarik** _____________

***Janeno en ng’at ma ok otudore gi nonro e yo moro amora**

Alama lwet ng’at maduong’/jarit nyathi kaka ondiki malo ka kapo ni ok ginyal ndiko______________

**JANONRO/JANYUOL/JARIT NYATHI KORO IMIYO OBKE KAKA MA MOKETIE SEI MONDO OKAN**

Wadwaro bende kano rembi/remb nyathini ma nitie e otas ma kende kar KEMRI/CDC man Kisian kuom higni abich.wabiro dwaro pimo ka nitie kido moko konyo malaria landore kendo miyo obedo matek thiedho gi yien. Rembi ibirokan gi namba moro ma kende ma jota nonro kende ema ong’eyo. Ok wabipimo tuoche mamoko e rembi/remb nyathini. Mar mondo wane ka pim gi dhi maber, wabiro oro moko matin mar remogi loka UK. Wakwayi mondo imiwa ayie ma kende mar oro remo loka.

Ka e thuolo mar nonro, ok idwar ni wamed kano rembi/remb nyathini, yie itudori kod Lkt. Jennifer Stevenson kat Chrispin Owaga kaka onyisi malo kae, bang’e to wabiro wito remogo e yo makare. Bang’ rumo nonro to wabiro golo yo moro amora ma nyalo tudo remono kodi/nyathini kendo ok wabinyalo bidho weg remogo.kata ka ok idwar ni okan rembi/remb nyathini to pod in/nyathini nyalo donjo e nonro. In/nyathini pod ibirotimne pim mar malaria kod remo matin. Ka in/nyathini nitie kod malaria kata remo matin, pod wabiro thiedhi/nyathini.

**Kano remo kuom thuolo ma lach ne nonro ma bang’e:**

Ayie ne KEMRI/CDC mondo okan remba/remb nyathina mantie e otas ne nonro ma bang’e. Ang’eyo ni anyalo loko pacha mondo kik kan remba/remb nyathina matie e otas ne nonro ma bang’e e thuolo mar higni abich. Anyalo bende kwayo ni remba/remb nyathina ok ti godo e pim moko. Mondo atim ma, anyalo nyiso Lkt. Jennifer Stevenson kwayona kendo obiro nyiso jota nonro man KEMRI/CDC.

Ket alama mar **X** e boksi achiel

| **AYIE:** |  | **Ayie mondo okan remba ne nonro mabiro bang’e** |
| --- | --- | --- |

| **OK AYIE:** |  | **Ok ayie mondo okan remba ne nonro mabiro bang’e** |
| --- | --- | --- |

**Nyathi ka hike tin ne 13: Nying____________________**

Apuodho ni aluwo yore duto mag nonro kaka ondiki ka akawo ayie mar bedo e nonro.

**Nying jataa nonro:_________**______

**Sei jataa nonro:___**_________

**Tarik ________________**

**Janyuol/Jarit nyathi: Nying________________________**

**KATA**

**Ng’at maduong’/Rawera mopong’: Nying_______________**

**Sei/alama mar lwedo:_________________**

**Tarik_________**

***Dwarore mana ka janyuol/jarit nyathi ok nyal somo:***

*Apuodho ni ler ewi nonroni ne ochiw malong’o kendo owinjore ne jagoni, ka ayie mar bedo e nonro bende ne ochiw gi jago madonjo e nonro owuon.

**Nying janeno:**  _____________________________________

**Sei mar janeno:** _____________________________________ **Tarik** _____________

***Janeno en ng’at ma ok otudore gi nonro e yo moro amora**

**JANONRO/JANYUOL/JARIT NYATHI KORO IMIYO OBKE KAKA MA MOKETIE SEI MONDO OKAN**

**Nyongeza B4: Fomu ya ruhusa: Ukaguzi wa Maleria (watu binafsi)**

**Kiswahili**

Kupunguza uzito wa ugonjwa wa malaria kwa kuelekeza juhudi mahali palipo ueneaji wa juu na kuimarisha juhudi za kupigana na malaria kwenye nyanda za juu za Kenya Magharibi: Udhibitaji wa pamoja wa njia nne za kupigana na malaria na uchunguzi wa manufaa kupitia utafiti wa safari moja moja.

| Tarehe ya leo | _ _ /_ _ / _ _ _ _ | Study/Cell/Compound/House code | V2__/ __ __ __ __/__ __ __ |
| --- | --- | --- | --- |
| Mkuu wa nyumba: Jina la kwanza ___________________________ | | Jina la katikati  _______________________ | Jina la mwisho  _____________________ |

Boma lako yako liko katika eneo la Rachuonyo lenye hupata malaria nyingi sana. Tungependa kufanya uchunguzi kwa boma lako ili kudhibitisha ikiwa mikakati dhidi ya malaria iliyotekelezwa hivi majuzi imaleta manufaa kwa kupunguza kesi za malaria. Kutia kidole kwa hi fomu kutaturuhusu kuuliza wewe na/au mtoto wako maswali, kupima joto la mwili na kutoa damu ya kidola ili kupimia malaria bomani au kwa maabara ya Kisumu, Uingeraza au Uholanzi. Mtu akipatikana na malaria, kila mmoja atapata dawa ya malaria bila malipo. Pia, kila anayekuwa na upungufu wa damu atatibiwa.

Hatutaweza kuwatibu wanawake wajaa wazito kwa huu utafiti. Wanawake wajaa wazito wenye ni wagonjwa watahitajika kwenda kwa kituo cha afya kumwona daktari. Ukiwa mwanamke aliye na umri wa miaka 13- miaka 45, tutakuuliza maswali kuhusu uwezekano wa kuwa na mimba. Ukiwa unaweza kuwa mjaa mzito na hauna uhakika, tutakuuliza ufanye uchunguzi wa kuwa na mimba hapa nyumbani ukitumia mkojo wako.

Mimi,_____________________________ nimelezewa kuhusi huu utafiti. Nimeelewa kuwa nimesomewa kamili na maswali yangu kujibiwa vilivyo. Ninaelewa kuwa ninaweza kubadilisha uamuzi wangu wakati wowote nah ii haitadhuru manufaa yanayofaa nyumba yangu. Weka alama ya **Y/N** kwa kisanduku moja:

| **NIMEKUBALI KUULIZWA MSWALI NA KUTOA DAMU KAMA ILIVYOELEZWA HAPA JUU:** |  |  |
| --- | --- | --- |

**Mtoto kama chini ya 13: Jina________________________**

Ninadhibitisha nimefuata kanuni zote za utafiti wakati ninachukuwa ruhusa hii.

**Jina la mtafiti:________________**_

**Seii ya mtafiti:__________**_

**Tarehe: _____________**

**Mzazi/Mlezi: Jina____________________________**

**AU**

**Mtu mzima/mtoto mkubwa: Jina_________________**

**Seii/alama ya kidole_____________________**

**Tarehe________________**

***Muhimu ikiwa mzazi/mlezi wa mtoto hawezi kusoma:***

Ninadhibitisha kuwa taarifa juu ya huu utafiti ulielezwa kamili na mshiriki kuelewa na kwamba ruhusa hii imetolewa kwa hiari.

**Jina la shahidi**: _________________________________________

**Seii la shahidi**: _________________________________________ **Tarehe ___________**

*** Shahidi ni mtu asiyeegemea utafiti kwa namna yoyote**

Alama ya kidole cha mtu mzima/mzazi ametajwa hapa juu kama hawezi kuandika:_____________

**MSHIRIKI/MZAZI/MLEZI ANATAKIKANA SASA KUPATIWA KOPI YA KARATASI HILI ILIYOTIWA KIDOLE ILI AWAKE**

Tungependa pia kuhifadhi sampuli ya damu yako/damu ya mtoto wako ilioyoko kwa kartatasi huko KEMRI/CDC kwa miaka mitano. Tungependa kupima ili tuone ikiwa kuna majijni fulani ambayo husaidia malaria kuenea na kuisababissha ngumu kutibu. Damu yako/damu yamtoto wako itahifadhiwa na nambari fulani ya siri inayujulikana tu kwa watafiti husika. Hatutachunguza damu yako/damu ya mtoto wako kwa magonjwa mengine. Ili kudhibitisha ubora wa uchunguzi tunaofanya, kiasi kidogo cha sampuli kitatumwa kwa uchunguzi Uingereza. Tunakuuliza ruhusa ya kipekeee ya kutuma sampuli ng’ambo.

Ikiwa wakati utafiti unapoendelea, utataka tusiendelee kuhifadhi damu yako/ya mtoto wako, tafadhali wasiliana na Dkt. Jennifer Stevenson au Chrispin Owaga kwa njia ya mawasiliano iliyo hapa juu na tutaharibu sampuli yako.ifikapo tamati ya uchunguzi, tuaondoa namna yote inaweza kukulinganisha/mtoto wako na sampuli, na hatutaweza kuitambua sampuli hiyo. Kama hutaki damu yako/ya mtoto wako kuhifadhiwa, wewe/mtoto wako bado wanaweza kuendelea kushiriki kwa utafiti. Wewe/mtoto wako bado atachunguziwa malaria na upungufu wa damu. Ukiwa/akiwa na malaria au upungufu wa damu, bado tutakutibu/tutamtibu mtoto wako.

**Uhufadhi wa sampuli na utafiti za baadaye:**

Naipatia KEMRI/CDC ruhusa ya kuhifadhi damu yangu/ya mtoto wangu iliyo kwa karatasi maalum kwa utafiti za baadaye. Ninaelewa kuwa ninaweza kubadilisha uamuzi wangu ili damu yangu/ya mtoto wangu isihifadhiwe kwa uchunguzi kwa kipindi cha miaka mitano. Ninaweza pia kuuliza kwamba damu yangu/ya mtoto wangu iliyo kwa karatasi maalum isitumike kwa aina fulani ya uchunguzi. Ili nifanye hivi, ninaweza kumjulisha Dkt. Jennifer Stevenson nia yangu na atawajulisha wahusika kule KEMRI/CDC.

Weka alama ya **X** katika kisanduku kimoja:

| **NIMEKUBALI:** |  | **Nimekubali damu yangu ihifadhiwe kwa utafiti za baadaye** |
| --- | --- | --- |

| **SIJAKUBALI:** |  | **Sijakubali damu yangu ihifadhiwe kwa utafiti za baadaye** |
| --- | --- | --- |

**Mtoto kama chini ya 13: Jina________________________**

Ninadhibitisha nimefuata kanuni zote za utafiti wakati ninachukuwa ruhusa hii.

**Jina la mtafiti:______________**____

**Seii ya mtafiti:___**_______________

**Tarehe _______________________**

**Mzazi/Mlezi: Jina____________________________**

**AU**

**Mtu mzima/mtoto mkubwa: Jina_______________________**

**Seii/alama ya kidole_____________________**

**Tarehe________________**

***Muhimu ikiwa mzazi/mlezi wa mtoto hawezi kusoma:***

Ninadhibitisha kuwa taarifa juu ya huu utafiti ulielezwa kamili na mshiriki kuelewa na kwamba ruhusa hii imetolewa kwa hiari.

**Jina la shahidi**: _________________________________________

**Seii la shahidi**: _________________________________________ **Tarehe ___________**

*** Shahidi ni mtu asiyeegemea utafiti kwa namna yoyote**

**MSHIRIKI/MZAZI/MLEZI ANATAKIKANA SASA KUPATIWA KOPI YA KARATASI HILI ILIYOTIWA KIDOLE ILI AWAKE**

**Appendix B5: Assent form: Community malaria surveys (individual level)**

**English**

**Highland MTC**- Reducing the burden of malaria by targeting hotspots of transmission and improving malaria control measures in the highlands of Western Kenya: Simultaneous rollout of four malaria control interventions and evaluation by cross-sectional surveys.

| Today’s date | _ _ /_ _ / _ _ _ _ | Study/Cell/Compound/House code | V2__/ __ __ __ __/__ __ __ |
| --- | --- | --- | --- |
| Head of House: First name ___________________________ | | Middle name  _______________________ | Last name  _____________________ |

- I am being asked to decide if I want to be in this research study.
- I am being asked if I want to be tested for malaria
- I know I will have a few drops of blood drawn from my finger and have my temperature taken
- If I have malaria I will also receive medicine for malaria
- I may be given medicine for malaria even if I am not feeling sick today.
- I asked and got answers to my questions. I know that I can ask questions about this study at any time.
- I know that I can stop being in this study at anytime without anyone being mad at me.

Mark box with **Y/N**:

| **I CONSENT TO BE INTERVIEWED AND HAVE MY BLOOD TAKEN AS DESCRIBED IN THE INFORMATION SHEET:** |  |  |
| --- | --- | --- |

I certify that I have followed all the study specific procedures described in the SOP for obtaining informed consent.

**Investigator’s name :_____________**__

**Investigator’s signature:** ____________

**Date ______**

**Child (13-17):Name**__________________

**Signature/thumbprint**: _______________**Date** __________

**Parent/guardian’s: Name_________________**

**Signature/thumbprint**: ________________**Date** ___________

***Only necessary if the parent/guardian cannot read:***

I *attest that the information concerning this research was accurately explained to and apparently understood by the subject/parent/guardian and that informed consent was freely given by the subject/parent/guardian.

**Witness’ name**: _____________________________________

**Witness’ signature:** _____________________________________ **Date** _____________

***A witness is a person who is independent from the study**

**THE SUBJECT/PARENT/GUARDIAN SHOULD NOW BE GIVEN A SIGNED COPY TO KEEP**

**Momedi B5: Oboke mar Ayie ewi nyithindo: Pimo ng’eny maleria e oganda (ng’ato owuon)**

**Dholuo**

**Highland MTC-** Duoko pek mar maleria piny ka idimbo dago ma landruok tuo ng’enyie kod jiwo yore mag mag gayo malerie e hosi mag gode man milambo mar Kenya: Riwo yore ang’wen mag gayo maleria kod nonro margi ma dichiel.

| Tarik ma kawuono | _ _ /_ _ / _ _ _ _ | Study/Cell/Compound/House code | V2__/ __ __ __ __/__ __ __ |
| --- | --- | --- | --- |
| Wuon ot: Nying mokwongo ___________________________ | | Nying madiere  _______________________ | Nying mogik  _____________________ |

- Ikwaya ni awachi ka adwaro donjo e nonro
- Ikwaya ka adwaro ni mondo opimna maleria
- Ang’eyo ni ibirokaw remo mtin ahinya e lith lweta kendo ibiropim liet mar denda
- Ka na kata ng’ato ang’ata nitie gi maleria e dalana to abiro yudo thieth mar maleria kata obedo ni aonge gi maleria
- Inyalo miya yadh maleria kata ka ok awing matuo kawuono
- Ne apenjo kendo ayudo dwoko mag penjona. Ang’eyo ni anyalo penjo ewi nonroni saa asaya
- Ang’eyo ni anyalo weyo bedo e nonroni ma onge ng’at makelo koko moro amora

Ket alama mar **Y/N** e boksi achiel

| **AYIE NE PIM KOD THIETH KAKA OLER E OBOKE:** |  |  |
| --- | --- | --- |

Apuodho ni aluwo yore duto mag nonro kaka ondiki ka akawo ayie mar bedo e nonro.

**Nying jataa nonro:_________**______

**Sei jataa nonro:___**____________

**Tarik ________________**

**Nyathi (13-17): Nying________________________**

**Sei/alama mar lwedo:_________________ Tarik____________**

**Janyuol/Jarit nyathi: Nying_______________________**

**Sei/alama mar lwedo:_________________ Tarik____________**

***Dwarore mana ka janyuol/jarit nyathi ok nyal somo:***

*Apuodho ni ler ewi nonroni ne ochiw malong’o kendo owinjore ne jagoni, ka ayie mar bedo e nonro bende ne ochiw gi jago madonjo e nonro owuon.

**Nying janeno:**  _____________________________________

**Sei mar janeno:** _____________________________________ **Tarik** _____________

***Janeno en ng’at ma ok otudore gi nonro e yo moro amora**

**JANONRO/JANYUOL/JARIT NYATHI KORO IMIYO OBKE KAKA MA MOKETIE SEI MONDO OKAN**

**Nyongeza B5: Fomu ya ruhusa kwa watoto: Ukaguzi wa Maleria (watu binafsi)**

**Kiswahili**

Kupunguza uzito wa ugonjwa wa malaria kwa kuelekeza juhudi mahali palipo ueneaji wa juu na kuimarisha juhudi za kupigana na malaria kwenye nyanda za juu za Kenya Magharibi: Udhibitaji wa pamoja wa njia nne za kupigana na malaria na uchunguzi wa manufaa kupitia utafiti wa safari moja moja.

| Tarehe ya leo | _ _ /_ _ / _ _ _ _ | Study/Cell/Compound/House code | V2__/ __ __ __ __/__ __ __ |
| --- | --- | --- | --- |
| Mkuu wa nyumba: Jina la kwanza ___________________________ | | Jina la katikati  _______________________ | Jina la mwisho  _____________________ |

- Ninaulizwa kuamua ikiwa ningependa kushiriki kwa utafiti
- Ninaulizwa ikiwa ninataka kupimiwa maleria
- Nitatolewa damu kidogo kwa kidole na kupimiwa joto la mwili
- Nikipatikana na maleria au mtu yeyote katika boma langu akipatikana na maleria nitapokea dawa za maleria hata kama sina maleria
- Ninaweza kupewa dawa za maleria hata kama sihisi mgonjwa leo hii
- Niliuliza maswali nikajibiwa. Ninajua ninaweza uliza maswali kuhusu huu utafiti wakati wowote
- Ninaju naweza kujiondoa kwa utafiti bila yeyote kuniletea maneno

Weka alama ya **Y/N** kwa kisanduku moja:

| **NIMEKUBALI KUULIZWA MSWALI NA KUTOA DAMU KAMA ILIVYOELEZWA HAPA JUU:** |  |  |
| --- | --- | --- |

Ninadhibitisha nimefuata kanuni zote za utafiti wakati ninachukuwa ruhusa hii.

**Jina la mtafiti:______________**____

**Seii ya mtafiti:___**_______________

**Tarehe _______________________**

**Mtoto (13-17): Jina**__________________________

**Seii/alama ya kidole_____________________**

**Tarehe________________**

***Muhimu ikiwa mzazi/mlezi wa mtoto hawezi kusoma:***

Ninadhibitisha kuwa taarifa juu ya huu utafiti ulielezwa kamili na mshiriki kuelewa na kwamba ruhusa hii imetolewa kwa hiari.

**Jina la shahidi**: _________________________________________

**Seii la shahidi**: _________________________________________ **Tarehe ___________**

*** Shahidi ni mtu asiyeegemea utafiti kwa namna yoyote**

**MSHIRIKI/MZAZI/MLEZI ANATAKIKANA SASA KUPATIWA KOPI YA KARATASI HILI ILIYOTIWA KIDOLE ILI AWAKE**

**Appendix C: Census list**

| Household information |  |  | |  |
| --- | --- | --- | --- | --- |
| Name of Head of compound: |  | | | |
| Village/EA |  | | | |
| Nearest Market |  | | | |
| Nearest primary school |  | | | |
| Nearest Health facility/  facility most frequented |  | |  | |
| Clan |  | | | |
| Cell – number |  | Compound number: | |  |
| Latitude: |  | Longitude: | |  |
| Elevation |  |  | |  |
| Fish pond owner Y/N |  |  | |  |

| House Number | Head of House | Number of treated nets in house | Number of untreated nets in house | Name of person | Sex | Year of birth | Daytime Location | Slept under net last night? | Net treated? |
| --- | --- | --- | --- | --- | --- | --- | --- | --- | --- |
|  |  |  |  |  |  |  |  |  |  |
|  |  |  |  |  |  |  |  |  |  |
|  |  |  |  |  |  |  |  |  |  |
|  |  |  |  |  |  |  |  |  |  |
|  |  |  |  |  |  |  |  |  |  |
|  |  |  |  |  |  |  |  |  |  |
|  |  |  |  |  |  |  |  |  |  |
|  |  |  |  |  |  |  |  |  |  |
|  |  |  |  |  |  |  |  |  |  |
|  |  |  |  |  |  |  |  |  |  |
|  |  |  |  |  |  |  |  |  |  |
|  |  |  |  |  |  |  |  |  |  |

**Appendix D. Net information and follow-up monitoring checks**

| Household information |  |  |  |
| --- | --- | --- | --- |
| Name of Head of Compound: |  | | |
| Village/EA |  | | |
| Cell – number |  | Compound number: |  |

|  | Initial distribution | | | | | | | 1 month follow up | | | | 6 month follow-up | | | |
| --- | --- | --- | --- | --- | --- | --- | --- | --- | --- | --- | --- | --- | --- | --- | --- |
| 1 | 2 | 3 | 4 | 5 | 6 | 7 | 8 | 9 | 10 | 11 | 12 | 13 | 14 | 15 | 15 |
| Date | House No | Head of House | ID  LLIN | Label Y/N | Usage  info  given  Y/N | Location  of net | Hanging  if N why | Net present (check ID ) | Net  Condition  (1-5) | Correct use | New net? Y/N if Y fill col.1-8 | Net present (check ID ) | Net  Condition  (1-5) | Correct use | New net? Y/N if Y fill col.1-8 |
|  |  |  |  |  |  |  |  |  |  |  |  |  |  |  |  |
|  |  |  |  |  |  |  |  |  |  |  |  |  |  |  |  |
|  |  |  |  |  |  |  |  |  |  |  |  |  |  |  |  |
|  |  |  |  |  |  |  |  |  |  |  |  |  |  |  |  |
|  |  |  |  |  |  |  |  |  |  |  |  |  |  |  |  |
|  |  |  |  |  |  |  |  |  |  |  |  |  |  |  |  |
|  |  |  |  |  |  |  |  |  |  |  |  |  |  |  |  |
|  |  |  |  |  |  |  |  |  |  |  |  |  |  |  |  |
|  |  |  |  |  |  |  |  |  |  |  |  |  |  |  |  |
|  |  |  |  |  |  |  |  |  |  |  |  |  |  |  |  |
|  |  |  |  |  |  |  |  |  |  |  |  |  |  |  |  |
|  |  |  |  |  |  |  |  |  |  |  |  |  |  |  |  |
|  |  |  |  |  |  |  |  |  |  |  |  |  |  |  |  |
|  |  |  |  |  |  |  |  |  |  |  |  |  |  |  |  |
|  |  |  |  |  |  |  |  |  |  |  |  |  |  |  |  |
|  |  |  |  |  |  |  |  |  |  |  |  |  |  |  |  |

**Appendix E. Questionnaire cross-sectional survey (4 parts) Transcipt from PDA**

(NB. Translations for questions in DhoLuo and Swahili (L, S respectively) are given only for those questions directed to household members and not for observational questions or for answers as all interviewers speak English)

**Part I-Household Information-Fill out once for each house**

1. Latitude: ____________________
2. Longitude___________________
3. Elevation___________________
4. District___________________
5. Location___________________
6. Area (cell) ___________________
7. House code _________________
8. Village Name___________________
9. Head of Compound___________________
10. Clan ________________________
11. Nearest Market ______________________
12. Nearest Primary School _______________________
13. Nearest Health Facility _________________________
14. What is the main occupation of the household head?

*L*: *Tich mane ma wuon odni timo*

*S: Ni kazi ipi mwenye nyumba hufanya?*

- 1. WORKS FOR PAY
  2. INCOME FROM SPOUSE OR OTHER RELATIVE
  3. UNPAID FAMILY BUSINESS
  4. WORKS ON FAMILY FARM
  5. UNEMPLOYED
  6. RETIRED

1. For the head of household, what is the highest level of education completed?

*L: Wuon odni osomo nyaka klas adi?*

*S*: *Ni kiwango kipi cha juu zaidi cha masomo ambacho mwenye nyumba ame hitimu?*

- 1. NONE
  2. PRIMARY INCOMPLETE
  3. PRIMARY COMPLETE
  4. Secondary INCOMPLETE
  5. SECONDARY COMPLETE
  6. higheR INCOMPLETE
  7. HIGHER COMPLETE
  8. UNKNOWN

1. What type of wall was used for the construction of this house?
   1. CLAY OR Mud (UNPLASTERED)
   2. SMEARED MUD
   3. MUD WITH CEMENT PLASTER
   4. IRON SHEETS
   5. brick OR STONE
   6. SOLID Cement
   7. other
2. What type of roof was used for the construction of this house?
   1. Grass thatch
   2. Iron sheet
   3. Tiles
   4. Other
3. How many windows have glass?
   1. NONE
   2. SOME
   3. ALL
   4. NO WINDOWS PRESENT
4. Are the eaves open or closed?
   1. OPEN
   2. Closed
   3. Partially Open
5. What is the main material of the floor?
   1. EARTH/SAND
   2. WOOD PLANKS
   3. CERAMIC TILES
   4. CEMENT
   5. OTHER)___________________
6. How many of the following animals are found in your compound?

*L: Jamni adi e kind magi ma un godo e dalau ka?*

*S*: *Wanyama wangapi kati ya hawa wanapatikana kwenye ua la nyumba yako?*

- 1. COWS (#): ______________
  2. GOATS (#): _____________
  3. SHEEP (#): ______________

1. What is the main type of fuel used by your family for cooking?

*L: En ang’o ma jo odni tiyogo kuom chweko e tedo mapile?*

*S: Unatumia aina gani ya nguvu au moto kupika?*

- 1. ELECTRICITY OR GAS/
  2. KEROSENE
  3. CHARCOAL
  4. FIREWOOD/STRAW
  5. DON’T KNOW
  6. OTHERS (SPECIFY) ________________

1. What is the main source of drinking water in your house?

*L: Ere kama jo odni goloe pi mar modho?*

*S: Uneyatoa wapi maki yako ya nyumbani ya kunywa?*

- 1. PIPED WATER IN RESIDENCE
  2. PUBLIC TAP/PUMP/PIPE
  3. WELL IN OWN COMPOUND
  4. PUBLIC WELL
  5. RAIN WATER
  6. RIVER/STREAM/SPRING OR OTHER SURFACE WATER
  7. OTHER _______________
  8. DON’T KNOW

1. What type of toilet do you have for your home?

*L: Ere kama jo odni tiyogo kuom losruok?*

*S: Una choo cha aina gani katika nyumba yako?*

- 1. OWN FLUSH TOILET
  2. SHARED FLUSH TOILET
  3. OWN CONCRETE PIT LATRINE
  4. SHARED CONCRETE PIT LATRINE
  5. OWN OTHER PIT LATRINE
  6. SHARED OTHER PIT LATRINE
  7. BUSH/FIELD AS LATRINE
  8. OTHER_____________
  9. DON’T KNOW

1. Observe whether there are the following in this house
   1. ELECTRICITY – YES/NO/DON’T KNOW
   2. SOLAR POWER` – YES/NO/DON’T KNOW
   3. TV – YES/NO/DON’T KNOW
   4. MOTORBIKE – YES/NO/DON’T KNOW
   5. BICYCLE – YES/NO/DON’T KNOW
   6. RADIO – YES/NO/DON’T KNOW
   7. 2 SEATER – YES/NO/DON’T KNOW
   8. CUSHIONS – YES/NO/DON’T KNOW
2. Does anyone in this house have a mobile phone?

*L: Bende nitie ngato modak e otka kod simo mar ong we yamo?*

*S: Je, kuna mtu yeyote katika nyumba hii ambaye ana rununu?*

- 1. YES
  2. NO
  3. DON’T KNOW

1. Who is the main user of the mobile phone?

*L: En nga ma hinyo tiyo kod simo ni?*

*S: Ni nani anayeitumia rununu hiyo mara kwa mara?*

- 1. MALE HEAD OF HOUSEHOLD
  2. WIFE
  3. OTHER MALE
  4. OTHER FEMALE
  5. CHILD
  6. DON’T KNOW

1. How frequently is the phone used?

*L: Simo mar ongwe yamo itiyogo marom nade?*

*S: Ni mare ngapi rununu hiyo hutumika?*

- 1. EVERY DAY
  2. EVERY WEEK
  3. FEW TIMES PER MONTH
  4. LESS FREQUENTLY
  5. DON’T KNOW

1. At any time in the past 12 months, has anyone sprayed the interior walls of your dwelling?

*L: E dweche 12 ma osekalo, bende osekir yath e kor odni gi iye?*

*S:* *Katika jumla ya miezi kumi na miwili iliyopita, kuna yeyote amenyunyizia dawa kwenye kuta za nyumba yako?*

- 1. YES
  2. NO
  3. DON’T KNOW

1. How many months ago was the house sprayed? (IF LESS THAN ONE MONTH, RECORD ‘00’ MONTHS AGO)

*L: Ma ne otimore dweche adi ma okalo?*

*S: Ni miezi mingapi imepita tangu nyumba yako inyunyiziwe dawa?*

___________________

1. Who sprayed the house?

*L: Ng’’ano mane okiro yath e odni?*

*S: Ni nani aliyeinyunyizia dawa nyumba yako?*

- 1. GOVERNMENT WORKER/PROGRAMME
  2. PRIVATE COMPANY
  3. HOUSEHOLD MEMBER
  4. OTHER (specify)___________________
  5. Don’t know

1. Have any of the following been used in your house over the last week?

*L: Bende usetiyo gi achiel kuom magi e otka e juma ma okaloni?*

*S: Kuna chochote kifuatacho ambacho kimetumiwa kwenye nyumba yako wiki iliyopita?*

- 1. MOSQUITO COILS?
  2. INSECTICIDE SPRAYS (e.g. DOOM)?
  3. REPELLENTS OINTMENTS/CREAMS
  4. BURNING LEAVES

**Please may I see where the household members sleep. This is so we can examine the places where mosquitoes may bite you at night.**

***L: Be unyalomia thuolo a ne kamajo odni ninde? Ma en nikech wadwa ng’eyo kwonde ma suna nyalo kayi gotieno.***

***S: Tafadhali naomba ruhusa nione mahala wakaazi wanyumba wanpolala? Ili tuweze kujua mahali unaweza umwa na mbu usiku.***

1. How many sleeping spaces are there? (Observation)

_________________

1. Does your household have any mosquito nets that can be used while sleeping?

*L: Bende odi ka nitiere e net ma itiyogo ka ji nindo?*

*S: Una neti ya kujifunikia wakati wa kulala ili kujizuia mbu?*

- 1. YES
  2. NO
  3. DON’T KNOW

**Part II-Net roster-Fill out questionnaire for each net in the house.**

1. How many bednets does this house have?

*L: Odni ni gi nede adi?*

*S: Nyumba hii ina neti ngapi?*

Number___________________

1. How many are currently in use?

*L: Nede adi mitiyo godo?*

*S: Neti ngapi zinatumika?*

Number ___________________

1. How many are currently not in use?

*L: Nede adi mok tigo sani?*

*S: Neti ngapi hazitumiki kwa sasa?*

Number ___________________

Net ID____________

1. Observed
   1. YES
   2. NO
2. How long ago did you obtain the net?

*L: Nedni ichako bedogo karango?*

*S: Mliipata neti hii wakati mgani?*

- 1. <6 MONTHS AGO
  2. 6-12 MONTHS AGO
  3. 1-3 YEARS AGO
  4. >3 YEARS AGO
  5. DON’T KNOW

1. Where did you get the net?

*L: Nedni ne ing’iewo kata iyudo kure?*

*S: Mliitoa wapi au mliinunua wapi neti hii?*

- 1. MARKET STALL/SHOP
  2. GOK HEALTH FACILITY
  3. PRIVATE HEALTH FACILTY
  4. MASS DISTRIBUTION CAMPAIGN (FREE NETS)
  5. NGO
  6. GIFT
  7. OTHER________________
  8. DON’T KNOW

1. How much did you pay for the net?

*L.Nedni ne ing’iewo pesa adi?*

*S. Neti ulinunua pesa ngapi?*

- 1. FREE
  2. NOT FREE; HOW MUCH?____________
  3. DON’T KNOW

1. Observe the brand of net:
   1. PERMANET
   2. OLYSET
   3. FREE NET PERMETHRIN
   4. SUPANET EXTRA PERMETHRIN
   5. SUPANET EXTRA DELTAMETHRIN
   6. NET PROTECT
   7. INTERCEPTOR
   8. SUPANET
   9. OTHER, SPECIFY_________________
   10. NO LABEL, DESCRIBE___________________
2. When you got the net was it already treated with an insecticide to kill insects or mosquitoes or did you have to treat it?

*L: E seche mane iyudo nedni bende ne oselwoke kod yath mageng’o suna?*

*S: Mlipoininua, ilikuwa imetibiwa na dawa ya kuwauwa au kuwafukuza mbu au wadudu?*

- 1. CAME TREATED
  2. NOT TREATED, CAME WITH KIT FOR TREATING
  3. NOT TREATED, NO TREATMENT KIT WITH IT
  4. DON’T KNOW

1. Since you got the mosquito net, was it ever soaked or dipped in a liquid to repel mosquitoes or bugs? (Not soap).

*L: Nyaka ichak bedo gi nedni, bende oselwoke kod yath mageng’o suna?*

*S: Tangu ulipoinunua neti hii, ume wahi kuiweka kwenye maji yaliyo na dawa ya kuuwa au kuwafukuza mbu na wadudu?*

- 1. YES
  2. NO
  3. DON’T KNOW

1. How long ago was the net last soaked or dipped? (If less than 1 month, enter ‘00’)

*L: Olwoke gi yadh suna ndalo adi ma osekalo?*

*S: Ni wakati mgani umepita tangu uiweke neti kwenye maji yaliyo na dawa*

- 1. LESS THAN 1 YEAR AGO, GIVE MONTHS___________
  2. MORE THAN 1 YEAR AGO
  3. DON’T KNOW

1. Was this net hanging last night?

*L: Bende ne nedni oyar e otieno mapiny orugo kwauono?*

*S: Neti hii ilitumika jana usiku kujikinga na mbu?*

- 1. YES
  2. NO: WHY NOT
  3. STILL PACKAGED
  4. KEPT FOR VISITORS
  5. OLD
  6. BEING WASHED
  7. OTHER SPECIFY_____________
  8. DON’T KNOW

**Part III-Household Listing-Fill out questionnaire for each person who stayed in house previous night. For children, pose the questions to the primary caretaker**

1. Name of person___________________
2. Is (NAME) male or female?
   1. MALE
   2. FEMALE
3. Is (NAME) available for interview?
   1. YES
   2. NO
4. Will (NAME) be available for interview at another time?
   1. YES, WHEN___________
   2. NO
5. Does (NAME) usually stay here?

*L: Bende [NAME] odak ga ka?*

*S: Mtu huyu [NAME] anaishi hapa?*

- 1. YES
  2. NO

1. Did (NAME) sleep here last night?

*L: Bende [NAME] ne onindo ka otieno mokalo?*

*S: [NAME] Amelala hapa jana usiku?*

- 1. YES
  2. NO

1. What is [NAME]'s date of birth?

*L: [NAME] nonyuol e higa ane?*

*S: Tarehe ya [NAME ] ya kuzaliwa?*

YEAR____________

MONTH__________

1. Is (NAME) attending school?

*L: Bende [NAME] dhi ga sikul?*

*S: Je [NAME] anaenda shule?*

- 1. YES
  2. NO
  3. DON’T KNOW

1. Which school is (NAME) attending? (include whether primary or secondary)

*L: Sikul mane ma [NAME] some?*

*S: Ni shule gani [NAME] anasoma?*

_______________________

1. Which class is (NAME) in?

*L: To [NAME] nie kilass adi?*

*S: [NAME] yuko darasa la ngapi*

______________________

1. Has (NAME) been ill with a fever at any time in the last 2 weeks?

*L: Bende [NAME] osebedo gi del ma ore e jumbe ariyo ma osekalo?*

*S: Katika wiki mbili zilizopita, [NAME] amekuwa mgonjwa na kusikia maumivu na joto mwilini?*

- 1. YES
  2. NO (SKIP TO 13)
  3. DON’T KNOW

1. Did [NAME] seek advice or treatment for the fever from any source?

*L: Bende ne omanyo ng’’ado rieko, kata thieth kuom del maore kamor amora?*

*S: [NAME] alitafuta huduma ya afya au matibabu popote?*

- 1. YES
  2. NO
  3. DON’T KNOW

1. Where did you seek advice or treatment? (Check all that apply)

*L: Ng’’ado rieko kata thieth ne omanyo kanye?*

*S: Alienda kutafuta wapi huduma au matibabu hayo?*

1. GOVT. HOSPITAL
2. GOVT. HEALTH CENTER
3. GOVT. HEALTH POST
4. MOBILE CLINIC
5. FIELD WORKER
6. OTHER PUBLIC
7. PVT. HOSPITAL/CLINIC
8. PHARMACY
9. PRIVATE DOCTOR
10. MOBILE CLINIC
11. FIELD WORKER
12. OTHER PVT. MEDICAL)__________________
13. SHOP
14. TRAD. PRACTITIONER
15. OTHER)___________________
16. Has [NAME] had a fever in the last 24 hours?

*L: Bende [NAME] osebedo gi del maore nyoro kata kawuono?*

*S: [NAME] amekuwa na maumivu na mwili wake kuwa na joto masaa ishirini na manne yaliyopita?*

1. YES
2. NO
3. DON’T KNOW
4. Has (NAME) taken any drugs in the last 2 weeks? (Check all that apply)

*L: Bende [NAME] osemwonyoe yath e jumbe ariyo ma osekalo?*

*S: [NAME] ametumia dawa yoyote wiki mbili zilizopita?*

- 1. SP/FANSIDAR
  2. CHLOROQUINE
  3. AMODIAQUINE
  4. QUININE
  5. COARTEM
  6. OTHER ANTIMALARIAL (SPECIFY)___________________
  7. ASPIRIN
  8. ACETAMINOPHEN/PARACETAMOL
  9. IBUPROFEN
  10. OTHER (SPECIFY)___________________
  11. DON’T KNOW

1. Did this person sleep under a net last night?

*L: Be ng’ani noninde e bwo net gotieno manyoro?*

*S: Je huyu alila chini ya neti jana usiku?*

- 1. YES
  2. NO
  3. DON’T KNOW

1. Select which bednet in the house (NAME) slept under last night.
2. If NO, why not? (Check all that apply)

*L: Ka da, nang’o?*

*S: Kama la kwanini?*

- 1. IT IS TOO HOT UNDER THE NET
  2. THERE IS NOT ENOUGH SPACE UNDER THE NET/I FEEL TOO CLOSED IN
  3. IT DOES NOT PROTECT AGAINST MOSQUITOES/INSECTS
  4. NO MOSQUITOES AROUND
  5. IT IS FOR ONLY CHILDREN/PREGNANT WOMEN
  6. BEDNET USED BY PARENTS
  7. BEDNET USED BY SIBLINGS
  8. BEDNET BEING WASHED
  9. BEDNET OLD
  10. BEDNET KEPT FOR VISITORS
  11. IT IS TOO EXPENSIVE/CANNOT AFFORD ENOUGH NETS FOR EVERYONE
  12. IT IS NOT THE RAINY/MALARIA SEASON
  13. CANNOT HANG IT OVER MY SLEEPING PLACE/SLEEPING OUTSIDE
  14. CHANGE MY SLEEPING PLACE TOO OFTEN
  15. DO NOT KNOW
  16. OTHER

**We are now going to ask some questions about where [X] has lived in the past, and where he has travelled to in recent months. The reason we are asking these questions is to find out whether [X] might have been at risk of getting malaria in other places.'**

*L: Koro adwaro penjo kuom kama [X] osebedo ka odakie e thuolo ma okalo, to gi kama osedhie wuoth e dweche matin mokalo. Penjagi konyowa ng’eyo ka onyalo yudo tuo mar malaria Kuonde moko opogore gi ka.*

*S: Tutakuuliza maswali kuhusu pale [X] ameishi tena pasipo hapa na kule ametembelea miezi iliyopita ya karibuni. Tunataka kuweza kujua kama [X] angeweza kupata malaria pahali pengine*

1. How long has (NAME) lived in “EA Name”?

*L: [NAME] osedak kae (Y) maromo nade?*

*S: Kwa muda gani [NAME] amekuwa akiishi hapa?*

1. < 6MONTHS
2. 6 – 12 MONTHS
3. 1-5 YEARS
4. >5 YEARS
5. DON’T KNOW
6. Apart from “EA Name” has (NAME) lived in any other EA for more than 6 months?

*L: Bende [NAME] osedak e EA moro ma opogore gi (Y) e thuolo mohingo dweche auchiel?*

*S: Tofauti na hii “EA Name” [NAME] amewahi kuishi kwa EA ingine kwa muda wa inazidi miezi 6?*

- 1. YES
  2. NO
  3. DON’T KNOW

1. Did (NAME) live outside “district name”?

*L: [NAME] ne odak oko mar district ni (“district name”)?*

*S: [NAME] amewai kuishi nje ya walaya “district name”?*

1. YES
2. NO
3. DON’T KNOW
4. In which district did (NAME) live?

*L:[NAME] ne odak e district mane?*

*S: Ni wilaya gani [NAME] amekuwa akiishi?*

District (drop down, including Don’t Know)

1. In which EA did (NAME) live, within Rachuonyo?

*L: [NAME] ne odak e EA mane ei Rachuonyo?*

*S: Kwa wilaya gain katika Rachuonyo?*

EA _________/Don’t know

1. For how long did (NAME) live in this other EA?

*L: [NAME] ne odak e gweng moroni maromo nade?*

*S: Kwa muda gani [NAME] amekuwa akiishi katika kijiji zinginne?*

1. LESS THAN 1 YEAR
2. BETWEEN 1 AND 3 YEARS
3. MORE 3 YEARS
4. DON’T KNOW

1. Apart from these two EAs has (NAME) lived in any other EA for more than 6 months?

*L: Mopogore gi EA (gweng) ariyogi Bende [NAME]osedak e EA (gweng) moro kendo kuom thuolo mohingo dweche auchiel?*

*S: isopokuwa vijiji viwili hivi [NAME] amewahi kuishi kwa kijiji kingine kwa muda inayozidi miezi 6?*

1. YES
2. NO
3. DON’T KNOW
4. Did (NAME) live outside “district name”?

*L: [NAME] ne odak oko mar “nying district”?*

*S: [NAME] emeishi nje ya wilaya?*

1. YES
2. NO
3. DON’T KNOW

1. Which district did (NAME) live in?

*L: [NAME] ne odak e district mane?*

*S: Ni wilaya gani [NAME] ameishi?*

District (drop down, including Don’t Know)

1. Which EA did (NAME) live in, within “district name”

*L: [NAME] ne odak e EA mane ei “nying district”*

*S: Ni EA gani [NAME] ameishi katika wilaya “jina la wilaya”*

EA _________/Don’t know

1. For how long did (NAME) live in this other EA?

*L: [NAME] ne odak e EA moroni maromo nade?*

*S: Ameishi huko siku ngapi?*

1. LESS THAN 1 YEAR
2. BETWEEN 1 AND 3 YEARS
3. MORE 3 YEARS
4. DON’T KNOW
5. Has (NAME) made any overnight trips outside of “EA Name” in the last 3 months?

*L: Be [NAME] osedhi e gweng moro e dweche adek mokalo?*

*S: [NAME] ametembelea kijiji kingine kwa kipidi cha miezi 3 zilizopita?*

1. YES
2. NO
3. DON’T KNOW
4. How many overnight trips has (NAME) made outside “EA” in the last 3 months?

*L: Bende (X) osedhi wouth ma onindo oko mar “nying EA” e thuolo mar dweche 3 mokalo?*

*S: [X] amekuwa na safari ngapi za usiku nje ya “EA” kwa kipindi cha miezi 3 zilizopita?*

Number________

(Don’t know=-1)

1. When did (NAME) come back from his/her most recent overnight trip?

*L: E wuodhe mogik [NAME] nodwogo kara ng’o?*

*S: [NAME] Alirudi lini kutoka safari yake ya mwisho*

1. STILL AWAY
2. <2 WEEKS
3. 2-4 WEEKS
4. >4 WEEKS
5. DON’T KNOW
6. How many nights did (NAME) spend away during this most recent trip?

*L: [NAME] ne oduogo ka oa e woudhe manonindoe oko mogik karang’o*

*S: Ni usiku ngapi [NAME] amechukua akiwa kwa safari yake ya mwisho*

Number________

(Don’t know=-1)

1. Was the most recent overnight trip outside of “Rachuonyo”?

*L: Otieno adi mane [NAME] onindoe oko e woudhe mogik?*

*S: Safari yako ya mwisho ilikuwa nje ya “Rachuonyo”?*

1. YES
2. NO
3. DON’T KNOW
4. In which district/s did (NAME) spend the most time during this most recent overnight trip?

*L: E distrikt mane ma [NAME] ne okawo thuolo mang’eny ewuoth mane onondo oko mogikni?*

*S: Ni wilaya ipi aliweza kukaa sana katika safari yake?*

District (drop down, including Don’t know) (skip to 24)

1. In which EA within Rachuonyo did (NAME) spend the most time during this most recent overnight trip?

*L: En EA mane ei Rachuonyo ma [NAME] yandeonindoe ahinya e woudhe maoko mogik?*

*S: Katika wilaya “EA” ipi [NAME] amechua muda mrefu katika safari yake ya mwisho katika wilaya ya Rachuonyo?*

EA _________/Don’t know

1. What was the main reason for making this most recent overnight trip?

*L: Ang’o momiyo ne odhi e wuodhe mogik ni?*

*S: Kwa nini alienda kwenye safari hii?*

1. WORK/LOOKING FOR WORK
2. BUYING/SELLING AT MARKET
3. ATTENDING SCHOOL OR UNIVERSITY
4. VISITING RELATIVES OR FRIENDS
5. FUNERAL
6. OTHER (SPECIFY) __________________
7. Did (NAME) make any other trips outside “EA name” in the last3 months?

*L: Bende [NAME] ne odhi wuoth moko oko mar “nying EA” e dweche 3 mokalo?*

*S: [NAME] amekuwa na safari nje ya nje “ Jina la EA” kwa muda wa miezi 3 zilisopita?*

1. YES
2. NO
3. DON’T KNOW
4. When did (NAME) come back from his/her most recent overnight trip?

*L: E wuodhe mogik [NAME] nodwogo kara ng’o?*

*S: [NAME] Alirudi lini kutoka safari yake ya mwisho*

1. STILL AWAY
2. <2 WEEKS
3. 2-4 WEEKS
4. >4 WEEKS
5. DON’T KNOW
6. How many nights did (NAME) spend away during this most recent trip?

*L: [NAME] ne oduogo ka oa e woudhe manonindoe oko mogik karang’o*

*S: Ni usiku ngapi [NAME] amechukua akiwa kwa safari yake ya mwisho*

Number________

(Don’t know=-1)

1. Was the most recent overnight trip outside of “Rachuonyo?

*L: Otieno adi mane [NAME] onindoe oko e woudhe mogik?*

*S: Safari yako ya mwisho ilikuwa nje ya “Rachuonyo?*

1. YES
2. NO
3. DON’T KNOW
4. In which district/s did (NAME) spend the most time during this most recent overnight trip?

*L: E distrikt mane ma [NAME] ne okawo thuolo mang’eny ewuoth mane onondo oko mogikni?*

*S: Ni wilaya ipi aliweza kukaa sana katika safari yake?*

District (drop down, including Don’t know) (skip to 24)

1. In which EA within “Rachuonyo did (NAME) spend the most time during this most recent overnight trip?

*L: En EA mane ei “Rachuonyo ma [NAME] yandeonindoe ahinya e woudhe maoko mogik?*

*S: Katika wilaya “EA” ipi [NAME] amechua muda mrefu katika safari yake ya mwisho katika wilaya ya“Rachuonyo*

EA _________/Don’t know

1. What was the main reason for making this most recent overnight trip?

*L: Ang’o momiyo ne odhi e wuodhe mogik ni?*

*S: Kwa nini alienda kwenye safari hii?*

1. WORK/LOOKING FOR WORK
2. BUYING/SELLING AT MARKET
3. ATTENDING SCHOOL OR UNIVERSITY
4. VISITING RELATIVES OR FRIENDS
5. FUNERAL
6. OTHER (SPECIFY) __________________
7. Are there any other persons who live in this house that we have not listed?

*L: Bendo nitiere ji mamoko manindo e odini mapodi ok andiko nying gi?*

*S: Kuna watu wengine wanaoishi kwenye nyumba hii ambao hatuna majina yao?*

1. YES (Go back to QUESTION 1 and fill out this form for that person)
2. NO
3. Are there any other people who may not be members of your family, such as domestic servants, lodgers or friends who usually live here?

*L: Bende nitiere ji mamoko ma ok jodala ka mapile, kaka jotich, wede, kata osiepe ma mane onindo ka otieno manyoro?*

*S: Kuna watu wengine wanaoishi kwenye nyumba hii kama marafiki, wafanyi kazi au watu wanaolipia malazi yao ambao hatuna majina yao?*

1. YES (Go back to person questionnaire start)
2. NO (Go to results page).

**Part IV-Malaria/Anaemia-Record the RDT result and anaemia result for each person**

**Person ID: _________________________**

1. Temperature:______________
2. Blood sample taken?
   1. YES
   2. NO, REASON NOT TAKEN
      1. REFUSED
      2. NOT PRESENT
      3. OTHER___________
3. RDT Result
   1. POSITIVE
   2. NEGATIVE
   3. INVALID
   4. NOT DONE , SPECIFY REASON____________

**If RDT positive, only offer treatment if the person is not pregnant* ***OR*** *has not been diagnosed with malaria and taken antimalarials in the past 2 weeks*

1. Hemocue reading___________________

**If HB is less than or equal to 110 treat with iron, if below 60 refer for immediate medical care*

1. Blood smear prepared?
   1. YES
   2. NO, REASON_____________

Record barcode number and date on blood slide

1. Filter paper blood spots prepared?
   1. YES
   2. NO, REASON_____________

Record barcode number and date on filter paper

1. Whole blood collected?
   1. YES
   2. NO, REASON_____________
2. Record Barcode number and date on microcontainer

Barcode Number: ________________________**Appendix F: Side effects monitoring form form insecticides**

Ask the head of house whether anyone in the house has suffered from the following symptoms since we sprayed the house/issued bednets

Area (Cell)_____________

Compound/house code_______________

| **Questions** | | | **Response (Y/N/DK)** |
| --- | --- | --- | --- |
| **ENGLISH** (Since we carried out IRS/gave bednets have you had…….. | **LUO** (Nyaka wa kir yath/chiw nede, be isebdo gi….. | **KISWAHILI** (Tangu tunyunyize dawa/gawa neti, je umehisi…… |  |
| **1.Headache** | Wich bar | Kuumwa na kichwa |  |
| **2. Itching/or rash** | Guonyruok/guonyo | Kuwashwa ama harara |  |
| **3. Difficulty in breathing** | Pek mar yueyo | Ugumu wa kupumua |  |
| **4.Dizziness** | Kawirawira | Kusinziasinzia |  |
| **5. Vomiting/nausea** | Ng’ok/chuny lep | Kutapika ama kuchafukwa na moyo |  |
| **6. Stomachache** | Ich kach | Kuumwa na tumbo |  |
| **7.Others/specify** | Mamoko/wachi | Mengineyo/fafanua |  |

If Y is answered to any of the above ask the head of the house which person/s suffered these symptoms and then call up each participant and complete the adverse reporting form. If serious also complete the serious adverse event form.

**Appendix G: Adverse reporting monitoring form for FSAT**

Call up each participant treated, **one by one**.

Area (Cell)_____________

Compound/house code_______________

Participant’s ID,.

Partcipant Name______________

Year of birth_________________

Sex _________M/F

Ask the participant about how they took AL:

- - 1. What time did you take your Coartem last night?

*S: Je ulimeza (dawa ya malaria) coartem saa ngapi jana usiku?*

*L: Ni mwonyo coartem (yadh malaria) saa adi otieno manyoro?*

________________

- - 1. How did you take the drugs?

*S: Ulimezaje hizi madawa?*

*L: Nimwonyo yadhgi nade?*

________________

- - 1. Did you take the drugs with any food/fluids?

*S: Je ulimeaza madawa na chakula au kinyuaji chochote?*

*L: Be nimwonyo yath gi chiemo kata math moramora?*

________________

Then ask the participant the following questions e.g. “In the last 24 hours have you had a Headache?” Write down the answer in the relevant column, like this: put a (**Y**) for “Yes”, a (**N**) for “No” and a “**DK**“ for “Don’t know”. Continue with the next problem and record the answers until all have been asked about. For an adverse event (i.e. where Y is recorded)., document each event on the following adverse reporting form. If any event is termed as severe/life threatening complete the second serious adverse event reporting form as well.

Below are the questions translated into Luo, and Kiswahili.

| **Questions** | | | **(Y/N/DK)** |
| --- | --- | --- | --- |
| **ENGLISH** (In the last 24hrs have you had…….. | **LUO** (Kuom seche piero ariyo gi ang’wen mokalo, be isebdo gi….. | **KISWAHILI** (Kwa masaa ishirini na manne yaliyopita,je umehisi…… |  |
| **1.Headache** | Wich bar | Kuumwa na kichwa |  |
| **2. Itching/or rash** | Guonyruok/guonyo | Kuwashwa ama harara |  |
| **3. Difficulty in breathing** | Pek mar yueyo | Ugumu wa kupumua |  |
| **4.Fatigue/body weakness** | Jony mar del | Uchovu ama unyonge wa mwili |  |
| **5.Dizziness** | Kawirawira | Kusinziasinzia |  |
| **6. Muscular pain** | Rem mar leche | Maumivu ya misuli |  |
| **7. Joint pains** | Rem mar fuonde | Maumivu ya viungo |  |
| **8. Vomiting/nausea** | Ng’ok/chuny lep | Kutapika ama kuchafukwa na moyo |  |
| **9. Fever** | Liet mar del | Joto jingi |  |
| **10. Stomachache** | Ich kach | Kuumwa na tumbo |  |
| **11.Others/specify** | Mamoko/wachi | Mengineyo/fafanua |  |

**Appendix H: Adverse event forms**

**H1: Adverse event reporting form**

| ADVERSE EVENT REPORTING FORM | | | | | | | CIRCLE AS APPROPRIATE:FSAT/ IRS/LLIN | | | | | | |
| --- | --- | --- | --- | --- | --- | --- | --- | --- | --- | --- | --- | --- | --- |
| **Person ID:**  ___________ | **Cell:** | | | | **Compound/house code:** | | | | **Date:**  \|___\|___\| / \|___\|___\| / \|___\|___\| | | | | |
|  | ***Complete on day first reported*** | | | | ***Complete on day first reported and update as needed*** | | | | | | | ***Complete on final day*** | |
| **Event** | **Date event onset (d)** | **Date event report (e)** | **Name person reporting** | **Max severity* (f)** | | **Max relationship† (g)** | | **Serious? ‡ *(Y/N)*(h)** | | **Episodic?**  ***(Y/N)*(i)** | **Outcome †† (j)** | | **Date event resolved‡‡ (k)** |
|  |  |  |  |  | |  | |  | |  |  | |  |
|  |  |  |  |  | |  | |  | |  |  | |  |
|  |  |  |  |  | |  | |  | |  |  | |  |
|  |  |  |  |  | |  | |  | |  |  | |  |
|  |  |  |  |  | |  | |  | |  |  | |  |

*** Severity:** Rank on scale of 1-4: mild = 1; moderate = 2; severe = 3, life-threatening = 4 **† Relationship:** Rank on scale of 0-4: none = 0; unlikely = 1; possible = 2; probable = 3; definite = 4

**‡ Serious:** Criteria for SAE: fatal, life-threatening, results in or prolongs hospitalization, results in significant or persistent disability or capacity requires medical / surgical intervention to prevent serious outcome.

If serious, report to core facility staff immediately.

**†† Outcome:** Rank on scale of 1-5: resolved without sequelae = 1; resolved with sequelae = 2; AE still present at study end/discontinuation, but improving = 3; subject died = 4; unknown = 5

**‡‡ Date event resolved:** Complete on Day 42 – If AE still ongoing at end of follow-up, indicate in question.

| SERIOUS adverse event form – initial report | | **Date of SAE Report:**  \|____\|____\|/\|____\|____\|/\|____\|____\|  *day month year* | | | |
| --- | --- | --- | --- | --- | --- |
| **Person ID:**  \|_____\|_____\|_____\|_____\|_____\|_____\|_____\| | | **Date enrolled:**  \|____\|____\|/\|____\|____\|/\|____\|____\|  *day month year* | | | |
| **Person name:** | | **Age:** \|_____\|_____\| years | | **Gender:**  Male Female | |
| **Parent name:** | |  | | | |
| **Event description**:__________________________________________________________________________________________________  *(symptom, sign, or laboratory abnormality)* | | | | | |
| **Indicate reason for serious AE**  *(tick all that apply):*  Death  Life-threatening  Resulted in or prolonged hospitalization  Required medical / surgical intervention to prevent serious outcome  Resulted in significant / persistent disability or incapacity Other:____________________ | **Date of event onset:**  \|____\|____\|/\|____\|____\|/\|____\|____\|  *day month year* | | | | **Maximum event severity:**  Mild  Moderate  Severe  Life-threatening |
|  | **Date of site awareness:**  \|____\|____\|/\|____\|____\|/\|____\|____\|  *day month year* | | | |  |
| **EVENT SUMMARY** *(include details of event, associated signs and symptoms, possible alternative etiologies, relevant past medical history, and medical management):*  *____________________________________________________________________________________________________________*  *____________________________________________________________________________________________________________*  *____________________________________________________________________________________________________________*  *____________________________________________________________________________________________________________*  *____________________________________________________________________________________________________________*  *____________________________________________________________________________________________________________* | | | | | |
| **Study product name:** Artemether-lumefantrine | | | **Route of study product:** Oral | | |
| **Dosing schedule at SAE onset:** | | | | | |
| **Date study product first started** *(Day 0)***:**  \|___\|___\|/\|___\|___\|/\|___\|___\|  *day month year* | | | **Date study product last taken prior to onset of SAE:**  \|___\|___\|/\|___\|___\|/\|___\|___\|  *day month year* | | |
| **Relationship to study product:**  None  Unlikely  Possible  Probable  Definite | | | **If not associated, is event related to:**  Study procedure  Other condition or illness  Other medication  Other____________________________ | | |

**H12: Serious adverse event form–initial report**

**H3: Serious adverse event form – management and outcome report**

| SERIOUS ADVERSE EVENT FORM – MANAGEMENT REPORT | |
| --- | --- |
| **Date of SAE Report:**  \|____\|____\|/\|____\|____\|/\|____\|____\|  *day month year* | **Study**  **Number:** \|_____\|_____\|_____\|_____\|_____\| |

| **Study product status:** *(tick all that apply)*  No change in dose  Study treatment held  Study treatment discontinued permanently  Other____________________________________ | | | | **Patient management:** *(tick all that apply)*  Patient hospitalized  Blood transfusion given  Intravenous fluids given  Parenteral quinine given  Other____________________________________  Other____________________________________  Other____________________________________ | | | | | | |
| --- | --- | --- | --- | --- | --- | --- | --- | --- | --- | --- |
| **CONCOMITANT MEDICATIONS** *(List relevant concomitant medications the subject was taking up to 1 month prior to SAE onset.)* | | | | | | | | | | |
| **Medication** | **Start date**  *(dd/mm/yy)* | **Stop date**  *(dd/mm/yy)* | | | **Total daily dose** | | **Indication** | | **Suspect**  **for SAE** | |
|  | \|___\|___\|/\|___\|___\|/\|___\|___\| | \|___\|___\|/\|___\|___\|/\|___\|___\| | | |  | |  | | □ Yes  □ No | |
|  | \|___\|___\|/\|___\|___\|/\|___\|___\| | \|___\|___\|/\|___\|___\|/\|___\|___\| | | |  | |  | | □ Yes  □ No | |
|  | \|___\|___\|/\|___\|___\|/\|___\|___\| | \|___\|___\|/\|___\|___\|/\|___\|___\| | | |  | |  | | □ Yes  □ No | |
|  | \|___\|___\|/\|___\|___\|/\|___\|___\| | \|___\|___\|/\|___\|___\|/\|___\|___\| | | |  | |  | | □ Yes  □ No | |
| **RELEVANT LABORATORY TESTS** | | | | | | | | | | |
| **Test** | **Collection date**  *(dd/mm/yy)* | **Result** | **Site normal range** | | | **Most recent value prior to SAE** | | **Collection date**  *(dd/mm/yy)* | | |
|  | \|___\|___\|/\|___\|___\|/\|___\|___\| |  |  | | |  | | \|___\|___\|/\|___\|___\|/\|___\|___\| | | |
|  | \|___\|___\|/\|___\|___\|/\|___\|___\| |  |  | | |  | | \|___\|___\|/\|___\|___\|/\|___\|___\| | | |
|  | \|___\|___\|/\|___\|___\|/\|___\|___\| |  |  | | |  | | \|___\|___\|/\|___\|___\|/\|___\|___\| | | |
|  | \|___\|___\|/\|___\|___\|/\|___\|___\| |  |  | | |  | | \|___\|___\|/\|___\|___\|/\|___\|___\| | | |
| **RELEVANT DIAGNOSTIC TESTS** | | | | | | | | | | |
| **Test** | **Collection date**  *(dd/mm/yy)* | **Results/Comments** | | | | | | | | |
|  | \|___\|___\|/\|___\|___\|/\|___\|___\| |  | | | | | | | | |
|  | \|___\|___\|/\|___\|___\|/\|___\|___\| |  | | | | | | | | |
|  | \|___\|___\|/\|___\|___\|/\|___\|___\| |  | | | | | | | | |
| **Outcome of event:**  Ongoing: [SAE Follow Up Report to be completed and sent at later date]  Resolved without sequelae  Resolved with sequelae _____________________________  Death | | | | | | **If resolved or died, indicate date:**  \|___\|___\|/\|___\|___\|/\|___\|___\|  *day month year* | | | | |
| **Completed by:**  **Name printed: ____________________________ Signature:** ___________________________ **Date:** \|___\|___\|/\|___\|___\|/\|___\|___\|  *day month year*  **Investigator’s**  **Name printed: ____________________________ Signature:** ___________________________ **Date:** \|___\|___\|/\|___\|___\|/\|___\|___\|  *day month year* | | | | | | | | | |  |

# H4: Guidelines for Grading Patient Symptoms, signs and laboratory findings

## Table A. Guidelines for grading patient symptoms

|  | **Grade 1**  **MILD** | **Grade 2**  **MODERATE** | **Grade 3**  **SEVERE** | **Grade 4**  **LIFE THREATENING** |
| --- | --- | --- | --- | --- |
| **Subjective fever in the past 24 h** | N/A | Present (Yes) | N/A | N/A |
| ***Weakness*** | Mild decrease in activity; For children – weak, but still playing | Moderate decrease in activity; For children – weak, and playing limited | Not participating in usual activities; For children – not playing | Prostration |
| **Muscle and/or joint aches*** | Mild and/or localized complaints | Diffuse complaints | Objective weakness; function limited | N/A |
| **Headache*** | Mild, no treatment required | Transient, moderate; treatment required | Severe, constant; requires narcotic therapy | Intractable; requires repeated narcotic therapy |
| **Anorexia** | Decreased appetite, but still taking solid food | Decreased appetite, avoiding solid food but taking liquids | Appetite very decreased; Refusing to breast feed, no solids or liquids taken (< 2 years < 12 hr; > 2 years < 24 hr) | Appetite very decreased; Refusing to breast feed, no solids or liquids taken (< 2 years > 12 hr; > 2 years > 24 hr) |
| **Nausea*** | Mild, transient feeling of impending vomiting; maintains reasonable intake | Moderate and/or constant feeling of impending vomiting; intake decreased | Severe, constant feeling of impending emesis; intake decreased significantly | N/A |
| **Vomiting** | 1 episode per day | 2-3 episodes per day | Orthostatic hypotension or IV fluids required | Hypotensive shock or hospitalisation required for IV fluid therapy |
| **Abdominal pain*** | Mild (1-3 on a scale of 1 to 10) | Moderate (4-6 on a scale of 1 to 10) | Moderate to severe (> 7 on a scale of 1 to 10) | Severe – hospitalilsation for treatment |
| **Diarrhea** | Transient 3-4 loose stools/day | 5-7 loose stools/day | Orthostatic hypotension or > 7 loose stools/day or IV fluids required | Hypotensive shock or hospitalilsation for IV fluid therapy required |
| **Cough** | Transient / intermittent | Persistent / constant | Uncontrolled | Cyanosis, stridor, severe shortness of breath |
| **Pruritis** | Transient pruritis | Pruritis that disturbs sleep | Severe, constant pruritis, sleep disturbed | N/A |
| **Tinnitus*** | Mild, transient ringing or roaring sound | Moderate, persistent ringing or roaring sound | Severe ringing or roaring sound with associated hearing loss | N/A |
| **Behavioural changes** | Mild difficulty concentrating; mild confusion or agitation; activities of daily living unaffected; no treatment | Moderate confusion or agitation; some limitation of activities of daily living; minimal treatment | Severe confusion or agitation; Needs assistance for activities of daily living; therapy required | Toxic psychosis; hospitalilsation required |
| **“Flu”**  **(viral URI)** | Mild nasal congestion, mild rhinorrhea | Moderate nasal congestion, moderate rhinorrhea | N/A | N/A |
| **Allergic reaction** | N/A | N/A | Urticaria | Severe urticaria  anaphylaxis, angioedema |
| **Convulsion** | N/A | N/A | Localized or generalized seizure | Status epilepticus |
| *** Assess only in children > 3 years of age. Answer N/A for younger children and those unable to answer.** | | | | |

Reference – Based on WHO Toxicity Grading Scale for Determining the Severity of Adverse Events
